# Choosing how to choose

**DOI:** 10.1007/s11238-025-10088-2

**Published:** 2025-10-08

**Authors:** Richard Pettigrew, Catrin Campbell-Moore, Jason Konek

**Affiliations:** Department of Philosophy, University of Bristol, Bristol, UK

**Keywords:** Decision-making, Uncertainty, Risk-sensitivity, Expected Utility Theory, Exploitability, Theories of rational choice

## Abstract

Decision theories give guidance about what to do when you face a particular decision. But they also give higher-level advice—depending on how likely you think it is that you’ll face various decision problems, decision theories give advice about the best strategy for picking what to do. For some ways of being uncertain over possible decisions, decision theories that accommodate risk undermine themselves. They simultaneously provide specific advice about what to pick whilst also deeming that very picking strategy to be impermissible. Savage-style expected utility theory does not have this flaw; it always deems its own picking strategies to be the rational ones. Popular decision rules for imprecise probabilities never straightforwardly undermine themselves but they often require adopting strategies that pick in accordance with expected utility theory, thereby coordinating how to pick across different potential decision problems. Some strategies that always pick an option that the decision theory does not reject are deemed impermissible. In particular, randomising amongst non-rejected options is often impermissible. This raises questions about how to use the guidance of an imprecise decision theory when it leaves different options non-rejected.

**Supplementary Information:**

The online version contains supplementary material available at https://doi.org/10.1007/s11238-025-10088-2.

It is our lot to face decisions when it is uncertain which act from among those available to us will lead to the best outcome. Uncertain about the day’s weather, you must choose whether or not to take an umbrella when you leave your house; uncertain about what it would help them most to hear, you must choose what to say to a friend who is going through a bad time; and uncertain what effect different approaches will have, a parent must choose how to raise their child. How are we to make such choices? It is the task of decision theory to provide an answer. And philosophers, economists, and psychologists have met this remit by developing a slew of rival theories of rational decision. Expected utility theory is the most well known and widely used, but there are many alternatives available, and we will meet a good few in the course of this paper.

There are various ways to argue for your preferred decision theory. You might note that it agrees with your intuitive verdict about a specific decision problem that you describe, while its rivals don’t. For instance, you might intuitively judge the Allais or Ellsberg preferences rationally permissible, and note that certain risk- or ambiguity-sensitive decision theories permit them, while expected utility theory does not. Or you might note that your favoured theory has a formal feature that you find intuitively desirable, while its rivals lack that feature. For instance, you might intuitively judge the Independence Axiom or the Sure Thing Principle a requirement of rationality, and note that expected utility theory satisfies both, while its risk-sensitive rivals don’t.

But there is another approach, and it has the advantage that it avoids such appeals to our intuitive judgments and the stalemates in which they often result. It begins with the observation that a decision theory is an account of rational means-ends reasoning: agnostic about whether your ends are good or bad, desirable or undesirable, benevolent or malign, it purports to tell you the rational way to pick between different possible means to the ends you in fact have. Granted this, it seems natural to assess a decision theory by asking how well it performs in the role of getting you those ends. The only problem with this approach is that, in order to assess a decision theory or anything else as a means to your ends, we need an account of which means to your ends it is rational to use. And without a decision theory, we don’t have that.

Yet all is not lost, for this line of thinking nonetheless furnishes us with a test we can conduct on a theory of decision-making, and while it might not tell in favour of the theory if it passes, it seems to tell against it if it fails. We can ask of the theory: If I were to use you not only to make my normal day-to-day decisions, but also to make the higher-order decision about which decision theory to use, would you recommend yourself? If it would, we call it *self-recommending*; if it wouldn’t, we call it *self-undermining*. We claim that no self-undermining decision theory can be correct. This is not to say that a self-recommending theory is thereby adequate—for instance, the theory that says that any available act is rationally permissible is self-recommending, but it is not correct. Nonetheless, we can use this test to winnow the list of candidate decision theories, removing those that fail it.

**Our forthcoming results**: In this paper, we show that this idea gives rise to a challenge for a host of theories that diverge from expected utility theory. The strength of our results varies over these different theories, and so we provide a summary here.

Decision theories that accommodate risk, by rationalising the Allais preferences, lead to ways of being uncertain over which decision problem you’ll face such that they deem their own recommendations impermissible. Such theories therefore undermine themselves in a particularly strong sense. However, this initial result is limited to particular ways of being uncertain about which decision problem you’ll face. We strengthen the result a little by showing the same follows for some natural ways of being uncertain over a much wider range of decisions, but we do not have a general result that holds for a whole host of ways of being uncertain (Sect. 1). It is thus still open to a defender of this theory to argue that they are not uncertain over which decision they will face in one of these seemingly natural ways; or at least they should not be.

Decision theories that accommodate ambiguity or imprecision also differ from expected utility theory. *Γ *-Maximin is such a decision theory, and we show that it is self-undermining in the same strong sense just described, at least for certain ways of being uncertain about which decision problem you’ll face (Sect. 3.1).

Two other prominent decision theories for imprecision are E-Admissibility and Maximality. These theories deem some actions impermissible, but there are often many actions they don’t rule out. There are thus typically many different ways of acting that are compatible with the recommendations of the theory. As before, we wish to use a decision theory to judge which decision theory to use by looking at the utility of what the decision theory to be judged recommends you pick. But these imprecise decision theories do not offer univocal recommendations concerning what to pick. Initially, then, we simply consider the theory’s judgements about what we call picking strategies, which specify, for each possible decision we might face, a specific act to pick from those that are available. We say that a picking strategy is almost surely an expected utility strategy if there is a probability function over the states of the world such that we are certain the strategy selects an act that maximizes expected utility relative to that probability function.

E-Admissibility deems a picking strategy impermissible just in case it is not almost surely an expected utility strategy (Sect. 3.2). Since picking in accordance with expected utility theory is also picking in accordance with E-Admissibility, this decision theory is not self-undermining in the strong sense we’ve considered so far: it does not rule out all ways of picking compatible with itself. However, it does rule some out. It is compatible with E-Admissibility to pick in accordance with different probability functions from the credal set when faced with different decision problems. Such picking strategies, however, are often deemed impermissible according to the theory. This theory therefore demands that a decision-maker coordinates across different possible decision problems to ensure there is a single probability function such that she almost surely chooses what maximizes expected utility from the point of view of that probability function whichever decision problem she faces.

The theory also deems it impermissible to pick in accordance with some randomisation procedure over those actions that are not ruled out, as again this does not amount to picking almost surely in accordance with expected utility theory applied to a specific probability function. We might then ask the defender of one of these theories how our decision-maker should use the non-committal recommendations of their theory? As we’ve seen, she should not randomise amongst the options that aren’t ruled out. Instead, again, she needs to select some probability function from her credal set and use that to determine her plan for what to pick, whatever decision she faces. It is an interesting question how this then differs from a permissivist approach to expected utility theory.

The same formal result also shows that E-Admissibility is self-undermining in the same sense if, rather than knowing you’ll randomize over the actions the theory hasn’t ruled out, you simply don’t know which you’ll pick, and you have precise credences over the possibilities that gives positive credence to each.

Next, we ask what happens if, instead of having precise credences concerning which actions you’ll pick, you have imprecise credences. We consider how to extend E-Admissibility to judge such imprecise ways of picking. We note that each probability function concerning how you’ll pick considers the imprecise picking strategy sub-optimal; it prefers instead to adopt a precise expected utility picking strategy. Thus, in the spirit of E-Admissibility, we suggest that the set of probability functions that represents your imprecise credences concerning how you’ll pick deems this imprecise picking strategy impermissible too; it again prefers that you select a probability function from the credal set and run with it.

Thus, whilst E-Admissibility is not strictly undermining in the way that *Γ *-Maximin and the risk-sensitive decision theories are, our results do force its defenders to think more carefully about what it means to adopt such a theory and how to make use of its advice. It is again an open question to what extent the version of E-Admissibility that renders it self-recommending is like a permissivist version of expected utility theory.

Maximality is an alternative prominent decision theory for imprecise probabilities. It is less committal than E-Admissibility (Sect. 3.2). Thus picking in accordance with expected utility theory according to one of the probabilities in the credal set is not rejected by the decision theory. We can again show that it deems impermissible any way of picking that does not amount to expected utility theory, at least when one’s uncertainty over which decisions you’ll face has some particular features. When your uncertainty over which decision you’ll face is precise and spread over a sufficiently broad range of decisions, the only ways to pick that are not impermissible are those that pick almost surely in accordance with expected utility theory. Again we see that this rules out picking by randomising over the acts that Maximality does not rule out, and it requires modal coordination in how one plans to use the recommendations of the theory.

What we see, therefore, is that considerations of whether the decision theory undermines itself lead to challenges for theories that diverge from expected utility theory by accommodating risk or imprecision. In the cases of imprecision, whilst not in a strong sense undermining, as only some ways of picking are deemed impermissible, such considerations force the defenders of these theories to think more carefully discuss how to use the theory.

## Risk-sensitive decision theories

Expected utility theory rules out as irrational certain natural ways of taking risk into account in decision-making. In particular, as noted above, it rules out the so-called *Allais preferences* (Allais, 1953). In response, decision theorists have presented a range of alternatives that permit those preferences (Kahneman & Tversky, 1979; Machina, 1982; Quiggin, 1982; Buchak, 2013). Let’s begin by showing a straightforward way in which any such decision theory is self-undermining.

Here are the four options over which the Allais preferences are defined. The payout of each depends on the outcome of a lottery with 100 tickets.

$$\begin{aligned} \begin{array}{r|ccc} D^{\textrm{Allais}}_1 & \text {ticket 1} & \text {ticket 2-11}& \text {ticket 12-100}\\ \hline p & {1}/{100} & {10}/{100} & {89}/{100} \\ \hline \hline \text {1A} & \text {\pounds1m} & \text {\pounds1m} & \text {\pounds1m} \\ \text {1B} & \text {\pounds0m} & \text {\pounds5m} & \text {\pounds1m} \end{array} \end{aligned}$$

$$\begin{aligned} \begin{array}{r|ccc} D^{\textrm{Allais}}_2 & \text {ticket 1} & \text {ticket 2-11}& \text {ticket 12-100}\\ \hline p & {1}/{100} & {10}/{100} & {89}/{100} \\ \hline \hline \text {2A} & \text {\pounds1m} & \text {\pounds1m} & \text {\pounds0m} \\ \text {2B} & \text {\pounds0m} & \text {\pounds5m} & \text {\pounds0m} \end{array} \end{aligned}$$

As Allais notes, many people prefer 1A to 1B in $D^{\textrm{Allais}}_1$ and 2B to 2A in $D^{\textrm{Allais}}_2$. Let’s suppose that our agent has a decision theory which endorses these preferences, given parameters such as her credences and utilities, and possibly also some representation of her attitudes to risk.

Now, consider instead a related decision problem, which we will call $D^{\textrm{Allais}}_{\textrm{local}}$. There are two actions available to the agent—Safe and Risky—whose outcomes depend on whether a particular company goes bust or not; for instance, they might be different stocks in which she can invest. Let’s suppose the agent thinks the odds of this company going bust are 1 : 10.

$$\begin{aligned} \begin{array}{r|cc} \text {Decision problem } D^{\textrm{Allais}}_{\textrm{local}} & \text {goes bust} & \text {not bust} \\ \hline p & {1}/{11} & {10}/{11}\\ \hline \hline \text {Safe} & \text {\pounds1m}& \text {\pounds1m} \\ \text {Risky} & \text {\pounds0m}& \text {\pounds5m} \end{array} \end{aligned}$$

Perhaps her decision theory prefers Risky to Safe.

We will now turn to a higher-order decision the agent might face. She is unsure which decision problem she will face, and she must choose a strategy for which option to pick in each possible decision problem. Perhaps she is leaving instructions for what her stockbroker should do, unsure what will be on offer. Or perhaps she has to choose a proxy to act on her behalf, knowing what they’ll pick in each decision problem, but unsure which decision problem they’ll face.

The options in this higher-order decision are what we call *picking strategies*. A picking strategy is a function, ${\mathsf {s}}$, which selects one of the available (first-order) options from each possible (first-order) decision problem. For example, a picking function will specify either Safe or Risky as its pick in $D^{\textrm{Allais}}_{\textrm{local}}$.

The choice between picking strategies is another kind of decision problem; this time, a higher-order decision problem. Our agent can make use of her decision theory to determine which picking strategy to choose, provided (i) she has credences over which decision problem she’ll face, and (ii) for each picking strategy, each decision problem, and each possible state of the world, she assigns a utility to adopting a picking strategy should she face that decision problem at that state of the world. To specify the latter, we evaluate a picking strategy by its fruits; that is, its utility when faced with a decision problem at a possible state of the world is the utility, at that state of the world, of the option it selects when faced with that decision problem.

Suppose our agent knows she’ll either face $D^{\textrm{Allais}}_{\textrm{local}}$ or she’ll be given $\text {\pounds1m}$ for sure. And suppose her credence that she’ll face $D^{\textrm{Allais}}_{\textrm{local}}$ is *11%*. She is deciding amongst various picking strategies. Here, the only relevant consideration in choosing amongst picking strategies is what they will pick when facing $D^{\textrm{Allais}}_{\textrm{local}}$. There are thus two options: ${\mathsf {s}}_{\textrm{Safe}}$ or ${\mathsf {s}}_{\textrm{Risky}}$, which pick Safe or Risky, respectively, in $D^{\textrm{Allais}}_{\textrm{local}}$. The decision among picking strategies thus amounts to this decision:

**Table:** 

Version 1	Choice offered		Choice not offered
	Goes bust	Not bust	
*p*	${1}/{11} \times {11}/{100}$	${10}/{11}\times {11}/{100}$	89/100
	= 1/100	= 10/100	= 89/100
${\mathsf {s}}_{\textrm{Safe}}$	$\text {\pounds1m}$	$\text {\pounds1m}$	$\text {\pounds1m}$
${\mathsf {s}}_{\textrm{Risky}}$	$\text {\pounds0m}$	$\text {\pounds5m}$	$\text {\pounds1m}$

But of course, this exactly mirrors the choice of 1A vs 1B in $D^{\textrm{Allais}}_1$. And so, assuming her decision theory is structural, in the sense that its recommendations don’t depend on the content of the outcomes but only on the list of credences of various utility outcomes, then she should also judge ${\mathsf {s}}_{\textrm{Safe}}$ to be preferable to ${\mathsf {s}}_{\textrm{Risky}}$, even though, when faced with $D^{\textrm{Allais}}_{\textrm{local}}$ herself, she prefers Risky.

Given the choice of what to write as instructions for her stockbroker, she prefers to instruct them to act differently from how she would act were she facing the decision herself. Given the choice over which proxy to nominate, she thinks it is better to nominate one she knows won’t choose in the way she would were she in the situation herself. Given the choice whether she should tie herself to the mast and pre-commit to the particular picking strategy she prefers before she knows which decision problem she’ll face, rather than leaving herself to choose after she comes to know, she reaches for that rope. Her adopted decision theory requires her to pick a picking strategy that is not compatible with its own recommendations. Despite the fact that her decision theory recommends picking Risky, it thinks it would be better to use a decision theory which recommends picking Safe.

This is a *prima facie* bad feature of our agent’s decision theory. Her decision theory makes recommendations both about what actions to perform when faced with different decision problems and which picking strategy is best. But these recommendations pull her in different directions. The decision theory itself tells you to do one thing when faced with the decision, but recommends using a picking strategy that does something else. This is a conflict in the theory’s recommendations. The oddity of the situation is akin to one that David Lewis (1971, 56) identified in a different context:

It is as if Consumer Bulletin were to advise you that Consumer Reports was a best buy whereas Consumer Bulletin itself was not acceptable; you could not possibly trust Consumer Bulletin completely thereafter.

The decision theory is self-undermining in the sense that there is some particular precise probability distribution over the possible decision problems she might face—it’s *11%* likely she’ll face $D^{\textrm{Allais}}_{\textrm{local}}$, and *89%* likely she’ll get $\text {\pounds1m}$ for sure—where, if she applies her decision theory to the question of which picking strategy to use, it rules out as impermissible its own recommended course of action.

This was all based on the assumption not only that her decision theory endorses the Allais preferences, but also that it recommends Risky over Safe in $D^{\textrm{Allais}}_{\textrm{local}}$. If it instead recommends Safe over Risky in $D^{\textrm{Allais}}_{\textrm{local}}$, we can consider a different decision problem: Suppose that, whilst she still thinks that her credence that she’ll be offered $D^{\textrm{Allais}}_{\textrm{local}}$ is *11%*, she thinks that if she is not offered it, then she is given nothing. Now, she is facing the following decision problem over which picking strategy to select.

**Table:** 

Version 2	Choice offered		Choice not offered
	Goes bust	Not bust	
p	1/100	10/100	89/100
${\mathsf {s}}_{\textrm{Safe}}$	$\text {\pounds1m}$	$\text {\pounds1m}$	$\text {\pounds0m}$
${\mathsf {s}}_{\textrm{Risky}}$	$\text {\pounds0m}$	$\text {\pounds5m}$	$\text {\pounds0m}$

This exactly mirrors the choice of 2A vs 2B in $D^{\textrm{Allais}}_2$, and we have supposed that she holds the Allais preferences of 2B over 2A. In this scenario, then, assuming again that her decision theory is structural in the sense described above, she will evaluate the picking strategy ${\mathsf {s}}_{\textrm{Risky}}$ to be preferable to ${\mathsf {s}}_{\textrm{Safe}}$, even though, when faced with $D^{\textrm{Allais}}_{\textrm{local}}$ herself, she prefers Safe.

Thus, if her decision theory endorses the Allais preferences and is opinionated over what to choose in $D^{\textrm{Allais}}_{\textrm{local}}$ then it is undermining in the sense that there is some uncertainty over which decision problem she’ll face where the decision theory rules its own way of picking as impermissible.

But what if her decision theory has neither a strict preference for Safe over Risky nor a strict preference for Risky over Safe in $D^{\textrm{Allais}}_{\textrm{local}}$? Then both ways of picking are compatible with her decision theory. Whilst which picking strategy she deems optimal changes depending on the amount she’ll receive when not facing $D^{\textrm{Allais}}_{\textrm{local}}$, in neither case does she rule out all the picking strategies that are compatible with her recommendations—she just rules out one of them. Whilst her decision theory would then be undermining in some sense, it is much weaker than what we had previously, where the decision theory rules out as impermissible *all* picking strategies compatible with its recommendations (there is only one).

However, with some additional assumptions we can modify the case to again show that the decision theory is self-undermining in the stronger sense. The decision theories that endorse the Allais preferences are usually motivated by avoidance of risk rather than considerations of ambiguity or imprecision. So they typically say that cases in which an agent doesn’t have a strict preference either way between two options are those in which she is indifferent between them; and, in those cases, any slight sweetening of one option is sufficient to make her strictly prefer that. So, in this decision, she will prefer $\hbox {Risky}^+$ to Safe:

$$\begin{aligned} \begin{array}{r|cc} D^{\textrm{Allais}}_{\textrm{local}^+} & \text {goes bust} & \text {not bust} \\ \hline p & {1}/{11} & {10}/{11}\\ \hline \hline \text {Safe} & \text {\pounds1m}& \text {\pounds1m} \\ \hbox {Risky}^+ & \text {\pounds1}& \text {\pounds5m}+ \mathrm{\pounds1} \end{array} \end{aligned}$$

What’s more, for some small enough sweetening, it is plausible that she will still prefer 1A to 1$$\hbox {B}^+$$^1^:

$$\begin{aligned} \begin{array}{r|cccc} & \text {ticket 1} & \text {ticket 2-11}& \text {ticket 12-100}\\ \hline p & {1}/{100} & {10}/{100} & {89}/{100} \\ \hline \hline \text {1A} & \text {\pounds1m} & \text {\pounds1m} & \text {\pounds1m} \\ 1\hbox {B}^+ & \text {\pounds1} & \text {\pounds5m}+ \mathrm{\pounds1} & \text {\pounds1m} \end{array} \end{aligned}$$

And so again, the decision theory deems it impermissible to pick in accordance with its own recommendations; instead it recommends using a different decision theory.

Any failure of the Marschak’s (1950) Independence Principle will generate a case where the decision theory is self-undermining. This principle says that, if the decision theory deems action $a_1$ preferable to action $a_2$, then it deems a probabilistic mixture $\alpha a_1+(1-\alpha )b$ preferable to $\alpha a_2+(1-\alpha )b$. Such a mixture is often interpreted as the act of using a randomization device, such as the toss of a biased coin, to determine which action to perform. But it can also be interpreted as evaluating picking strategies if you’re uncertain over which decision problem you’ll face. With probability *α *, you’ll face the decision between action $a_1$ and action $a_2$; with probability *1-α *, you’ll face the decision with just one option, namely, action *b*. The picking strategies are determined by their pick of either $a_1$ or $a_2$, and they exactly mirror the mixed acts. So, if a decision theory gives rise to a failure of the Independence Principle in which it prefers $a_1$ over $a_2$, but $\alpha a_2+(1-\alpha )b$ over $\alpha a_2+(1-\alpha )b$, then it will prefer strategy ${\mathsf {s}}_{a_2}$, which picks $a_2$ over $a_1$, over strategy ${\mathsf {s}}_{a_1}$, which picks $a_1$ over $a_2$, and yet ${\mathsf {s}}_{a_1}$ is the strategy that does what the decision theory demands. So, it is self-undermining.^2^

These cases are formally related to cases of sequential incoherence for such decision theories (Hammond, 1988; Machina, 1989), but the formal motivation for our investigation was instead Levinstein (2017) who, following Schervish et al. (2009), also considers uncertainty over which decision problem we face, and offers evaluations on that basis. The aim of their investigation is to judge and compare credences rather than decision theories. They hold fixed expected utility theory as the decision theory and instead use this uncertainty over which decision you’ll face to evaluate credences by their guidance value, assuming what they guide you to do is in accordance with expected utility theory.

### Is it a problem to be self-undermining?

There are at least two ways to argue that being self-undermining tells against a decision theory. We’ll describe them and then consider some responses.

First: Suppose a self-undermining decision theory is correct. That is, it deems impermissible exactly those options that are indeed impermissible. But, since it is self-undermining, it deems impermissible the option of picking in accordance with itself. And so it is indeed impermissible to pick in accordance with it, since it is correct. But surely it cannot be impermissible to pick in accordance with the true theory of rational choice. Therefore, the decision theory cannot be correct, which gives a reductio.

You might think that it could be impermissible to pick in accordance with the correct theory of rational choice, if for instance it is very costly to do so and there is a low cost alternative available that reasonably approximates the true theory, or if your attempt to pick in this way is likely to misfire, or if it is simply infeasible and so impossible for you to do it. But, when a theory is self-undermining, it is not for any of those reasons that it deems itself impermissible: it says it is impermissible even if it is cost-free to use it, and your attempt to use it always goes perfectly.

Second: Relatedly, for someone who uses the decision theory, it gives contradictory advice at different points. Recall the case above in which you were initially uncertain whether you’d face $D^\textrm{Allais}_\textrm{local}$ or get $\text {\pounds1m}$ for sure, and your decision theory would choose Risky over Safe when faced with $D^\textrm{Allais}_\textrm{local}$ but would choose the picking strategy ${\mathsf {s}}_\textrm{Safe}$ over ${\mathsf {s}}_\textrm{Risky}$. Standing facing the decision problem $D^\textrm{Allais}_\textrm{local}$, you might ask yourself: my decision theory tells me to choose Safe and not to choose Risky, but it also tells me that, if I could have chosen how I would choose, it would have told me to choose Risky and not to choose Safe—which should I do?

If we again suppose that the self-undermining decision theory is correct, it leads to a rational dilemma, where one is rationally required to adopt a picking strategy which picks in an rationally impermissible way.

These self-undermining decision theories would prefer to bind themselves to do something other than what they recommend when you actually face that decision. Of course, we’re used to that in cases of temptation—Ulysses should pay his sailors to bind him to the mast as their ship passes the Sirens—but that’s because we think your utilities will change under the pressure of temptation or your probabilities will change in an irrational way or you won’t choose rationally on the basis of your utilities and probabilities. Nothing like that is going on here.

We’re also used to that in cases of act-state dependence. Causal decision theorists say that in Newcomb’s problem you should take both boxes, but if you can pay to take a pill to turn yourself into a one-boxer before the prediction is made, then you should. But that’s because choosing how to choose, in this case, causes you to face a better decision problem down the line. Nothing like that is going on here. And perhaps we think that, at least when choosing a picking strategy has no causal or evidential impact on which decision problems you will face or the value of the options in those problems, the correct theory of rational choice should not give rise to such dilemmas.

### Responses

#### Limit the decision theory’s scope

Perhaps the defender of a self-undermining decision theory will say it was never their intention that their theory should be used for these higher-order decisions. They might say they are offering a theory of first-order rational choice; not a fully general theory that covers any sort of decision, including these higher-order decisions between picking strategies.

But that can’t be right, for these theories are presented by their proponents as universal decision theories—they are intended to cover any sort of decision, providing we can determine credences and utilities and any other parameters that must be fixed. In their descriptions, it is not specified that they are to be applied only to a certain sort of decision, such as decisions between first-order actions. They are intended to be what above we called structural: that is, their recommendations don’t depend on the content of the outcomes, but only on the list of credences of various utility outcomes. And, as we saw in our treatment of the Allais decisions above, for any higher-order decision between picking strategies under uncertainty about the decision problem you’ll face, there is a decision between ordinary first-order actions that exactly mirrors it. If the defender of a self-undermining decision theory were to respond in the way outlined, they’d have to deny that their decision theory is structural in this sense and they’d have to say why that is the case.

#### Change the way of updating credences

There is another way the defender of these theories could argue against the charge of self-underminingness. They can note first that, by assuming decisions are probabilistically independent of states of the world, and assuming our credences in states of the world don’t change when we learn which decision we face, we have essentially assumed Bayesian conditionalization. And then they can argue against conditionalization. As pointed out in Campbell-Moore and Salow (2020, 16), several standard arguments for conditionalization assume that rational agents maximise expected utility.

The picking strategy that the decision theory deems optimal is compatible with that very decision theory if instead we allow our agent’s credences to change when she learns which decision problem she faces, even though they are probabilistically independent. Instead of holding fixed her credences (assuming conditionalization), the defender of the decision theory might instead argue for an alternative credal update strategy: the one that results in the optimal picking strategy when coupled with risk-weighted expected utility theory (see also Campbell-Moore & Salow, 2022; Brown, 1976).

Whilst we acknowledge this is a possible way out of the criticism, it is a significant bullet to bite.

#### Uncertainty about decisions

In our argument that Allais-permitting decision theories are self-undermining, we used the preferences they permit over the Allais gambles to construct a *particular* way that you might be uncertain about what decisions you’ll face and showed that, *in that case*, you judge some alternative picking strategy to be preferable.

Moreover, the particular way of being uncertain over decisions you’ll be faced with was a rather odd one. We specified that the credence you’ll face $D^{\textrm{Allais}}_{\textrm{local}}$ is 1/11 and your credence that you’ll get the constant amount is 89/100, and we chose the constant specifically to demonstrate the undermining feature.

Of course this might be the situation you’re facing, where you happen to have these credences over the possible decisions. It might be just unfortunate coincidence, or it could be specifically constructed this way by a nefarious opponent who has set you up: they’re going to toss a biased coin to determine what to offer you at the specified probabilities, having chosen these numbers specifically to demonstrate the inconsistency in your judgements.

However, typically one’s uncertainty over which decision you’ll face is spread over a wide class of possible decision problems—how many decision problems are you fully certain you won’t face? If your particular uncertainty is not one that generates underminingness, and so, relative to that uncertainty, your decision theory judges its own picking strategies to be optimal, is it really so problematic that, were your uncertainty different in some specific way, your decision theory would be undermining?

This is analogous to a certain standard response to the Dutch book arguments: perhaps you just think it’s unlikely you’ll ever face such a nefarious bookie. A common response is to argue that the mere existence of a Dutch book already shows you are irrational because it shows your preferences are inconsistent in a particular way—they judge the same decision differently when it is presented in different way, perhaps.^3^ The set-up in which an opposing Gambler approaches you, buys and sells bets that you deem fair, and thereby saddles you with a sure loss merely dramatizes this inconsistency. The argument doesn’t assume you’ll ever actually meet such a person. We might argue similarly that the mere existence of self-underminingness for some way of being uncertain about what decision you’ll face is already a challenge to the decision theory. Perhaps it shows that it is inconsistent in the same way the Dutch book argument shows non-probabilistic credences are inconsistent.

In the case of the Dutch book arguments, Pettigrew (2020, Sec 6.2) argues that the mere existence of a set of bets you’ll accept individually that, taken together, lead to sure loss isn’t sufficient to show you are irrational. Instead, he asks what happens if you are uncertain about which decisions you’ll face. Drawing on the results from Mark Schervish (1989) and Ben Levinstein (2017) that we mentioned above, he shows that, for very many natural ways of being uncertain about the decisions you’ll face, if you have non-probabilistic credences and face whatever decision you’ll face with those credences, there are alternative probabilistic credences you might have had instead that guide your choices better.

We might similarly try to bolster our objection here by showing a more general result, which would apply to a whole host of natural ways of being uncertain over which decision problem you’ll face. Of course, we would have a stronger objection if we could show that for *any* way of being uncertain over the decision problems you face, the theory is self-undermining. In fact, we’ll never get something as general as that: after all, if your probability distribution places all of its probability on a single decision problem, then it will think of itself as permissible—and indeed, it will think of anything that disagrees with it as impermissible. But we might hope to be able to show that it is self-undermining for a much broader range of distributions over the possible decision theories than we currently have, thus arguing that, for any ‘plausible’ way of being uncertain over possible decisions, the theory is still undermining.

We don’t have any general results in this area, but we do have some suggestive particular cases for a particular Allais-permitting theory. This is John Quiggin’s (1982; 1993) rank-dependent utility theory, which is formulated for exogenous, objective probabilities, and Lara Buchak’s (2013) risk-weighted expected utility theory, which is a formally equivalent theory formulated for endogenous, subjective probabilities.

To give your *risk-weighted expected utility* for an act *a*, defined over possible states of the world in *Ω *, we need a probability function *p* over *Ω *, and a risk function *r : [0, 1] → [0, 1]*, which takes a probability and skews it—we assume *r* is continuous, strictly increasing, and *r(0)= 0* and *r(1) = 1*. Now, suppose *a* is an act, and let ${\mathfrak {U}}(a)$ be the random variable that gives the utility of *a* at a given state. If *Ω * is finite, the risk-weighted expected utility of *a* can be written as follows:^4^

$$\begin{aligned} \mathrm{R}\mathsf{Exp}_{p, r}[{\mathfrak {U}}(a)] = \sum _{x \in \lbrace {{\mathfrak {U}}(a)(\omega )\vert \omega \in \Omega } \rbrace } (r(p({\mathfrak {U}}(a) \geqslant x)) - r(p({\mathfrak {U}}(a) > x)))\times x \end{aligned}$$

And, more generally, if the utilities are bounded below by *l* and above by *h*,

$$\begin{aligned} \mathrm{R}\mathsf{Exp}_p[{\mathfrak {U}}(a)] = \int ^h_{l} r(p({\mathfrak {U}}(a) > x))\, dx \end{aligned}$$

Then risk-weighted expected utility theory tells you to maximize risk-weighted expected utility.

Now, suppose:

(i) there are just two first-order possibilities $\omega _1$ and $\omega _2$,
(ii) your credence function is *p*, with $p(\omega _1) = {0.1},  {0.2}, \ldots , {0.8}$, or 0.9 and $p(\omega _2) = 1-p(\omega _1)$;
(iii) your risk function is a power function $r_k(x) = x^k$, with $k = {0.5},  {0.6},\ldots, {0.8},  {0.9},  {1.1},  {1.2}, \ldots , {1.9},$ or 2;
(iv) you know you’ll face a choice between just two acts, but you don’t know which two acts, and you place a measure *μ * over the possible decision problems that takes the utilities of the two acts at the two possibilities to be independent of one another and all distributed according to a beta distribution $\textrm{Beta}(\alpha , \beta )$ with *α = 1, 2, 3, 4*, or 5 and *β = 1, 2, 3, 4*, or 5.

Then, let ${\mathsf {s}}$ be any picking strategy compatible with REU when coupled with *p* and $r_k$. Then there is an alternative picking strategy ${\mathsf {s}}^*$ such that REU with credences given by *p* and *μ * and risk function $r_k$ strictly prefers ${\mathsf {s}}^*$ to ${\mathsf {s}}$. What’s more, ${\mathsf {s}}^*$ is not compatible with REU with *p* and $r_k$. And indeed, it’s possible to find ${\mathsf {s}}^*$ so that it is compatible with REU with *p* and $r_{k^*}$ for some $k^* \ne k$. That is, ${\mathsf {s}}^*$ is a picking strategy compatible with REU coupled with a different risk-averse risk function. So, uncertain which decision they’ll face, someone using REU with this risk function would prefer that, when the uncertainty is resolved and they face a particular decision, they use REU with a slightly different risk function.^5^

## Expected utility theory

In this section, we reassure ourselves that Savage-style expected utility theory is self-recommending; that is, if we assume act-state independence, expected utility theory endorses itself. We will need to be more detailed about the framework in order to present our result.

*States* *Ω * is the set of possible states of the world. We’ll assume there are only finitely many.

*Uncertainty * The agent’s uncertain beliefs about the world are represented by a single probability function *p* over *Ω *. The set of all such probabilities is ${\mathcal {P}}$.

*Acts* ${\mathcal {A}}$ is a non-empty set. It is the set of all possible acts.

*Utilities* ${\mathfrak {U}}$ is the agent’s utility function. It takes each act *a* in ${\mathcal {A}}$ and state *ω * in *Ω * and returns a utility value ${\mathfrak {U}}(a)(\omega )\in {\mathbb {R}}$. We will assume that utilities are bounded above and below. That is, there are $l,h\in {\mathbb {R}}$ such that for all acts, $a\in {\mathcal {A}}$, $l\leqslant {\mathfrak {U}}(a)(\omega )\leqslant h$.^6^

*Decision problems * A decision problem *D* specifies a non-empty finite set of acts: the acts that are available in that decision problem. The set of all relevant decision problems is ${\mathcal {D}}$.

(In fact, we could relax the assumption that *D* specifies a finite set of acts and instead assume that the set of acts it specifies is compact relative to the utility function, that is, $\{{\mathfrak {U}}(a)\vert a \in D\}$ is a compact subset of $[l,h]^\Omega$. But we will continue to assume that decision problems are finite for ease of presentation.)

*Choice function * A choice function, ${\mathsf {c}}$, specifies a non-empty subset of each decision problem, so that $\varnothing \ne {\mathsf {c}}(D) \subseteq D$, for each *D* in ${\mathcal {D}}$. If *a* is *not* in ${\mathsf {c}}(D)$ then ${\mathsf {c}}$ deems *a* impermissible in *D*. Some authors go further and say that any *a* in ${\mathsf {c}}(D)$ is rationally *permissible* (e.g., Moss, 2015). But others do not. They instead say that, if *a* is in ${\mathsf {c}}(D)$, then *a* is not deemed impermissible, but unless *a* is the only act in ${\mathsf {c}}(D)$, it does not follow that *a* is permissible or positively evaluated in any way (e.g., De Bock, 2019).

*Picking strategy * As above, a picking strategy, ${\mathsf {s}}$, specifies an act from each decision problem, so that ${\mathsf {s}}(D)\in D$, for each *D* in ${\mathcal {D}}$. The set of all picking strategies is ${\mathcal {S}}$.^7^

A picking strategy picks for a choice function ${\mathsf {c}}$ if it never picks an option that ${\mathsf {c}}$ deems impermissible. That is:

### Definition 2.1

A picking strategy ${\mathsf {s}}$ *picks for a choice function* ${\mathsf {c}}$, if for all decision problems, *D*, ${\mathsf {s}}(D) \in {\mathsf {c}}(D)$.

Given a probability function *p* defined over *Ω *, the expected utility of an action *a*, by the lights of *p*, is given by

$$\begin{aligned} \mathsf{Exp}_p [{\mathfrak {U}}(a)]:=\sum _{\omega \in \Omega }p(\omega ){\mathfrak {U}}(a)(\omega ) \end{aligned}$$

If one has (probabilistic) credences given by *p*, expected utility theory says that one should choose an act in *D* that maximises $\mathsf{Exp}_p[{\mathfrak {U}}(a)]$.^8^ That is, we define the choice function to which expected utility theory gives rise when coupled with probability function *p* as follows:

### Definition 2.2

(Expected Utility Theory ($\textrm{EU}_p$))

$$\begin{aligned}\textrm{EU}_p(D):=\left\{ a\in D\vert \text { for all } a'\in D, \mathsf{Exp}_p[{\mathfrak {U}}(a)]\geqslant \mathsf{Exp}_p[{\mathfrak {U}}(a')]\right\} \end{aligned}$$

So a picking strategy ${\mathsf {s}}$ picks for $\textrm{EU}_p$ iff ${\mathsf {s}}(D)$ maximizes expected utility by the lights of *p*. Since we have assumed that each *D* is finite, or compact, $\mathsf{Exp}_p[{\mathfrak {U}}(a)]$ obtains its maximum in *D*; so $\textrm{EU}_p(D)\ne \varnothing$.

Now we want to judge the expected utility of a picking strategy itself—we want to ask whether the picking strategies that always pick an act that maximizes expected utility from any decision problem themselves maximize expected utility when you’re uncertain which decision problem you’ll face. This requires us to fix not only *p*, which gives your credences over *Ω *, but also your credences over the decision problems you might face, given by some probability measure *μ * over ${\mathcal {D}}$. We will assume these are independent. So your credences over the joint space, $\Omega \times {\mathcal {D}}$, are given by the product measure $p\times \mu$. That is, your credence you are in world *ω * and will face a decision problem in the (measurable) set of decision problems *E* is given by $(p\times \mu )(\omega ,E)=p(\omega )\times \mu (E)$. We will moreover always assume that *μ * is countably additive.

We can now simply apply our notion of expected utility with the credences over $\Omega \times {\mathcal {D}}$ given by $p\times \mu$. We judge a picking strategy by the utility of the acts it picks, and so we define ${\mathfrak {U}}({\mathsf {s}})(\omega ,D):={\mathfrak {U}}({\mathsf {s}}(D))(\omega )$. We can then apply our definition of expected utility and get that any picking strategy that picks for $\textrm{EU}_p$ maximizes expected utility by the lights of $p\times \mu$.

### Proposition 2.3

For any *p*, *μ *, if ${\mathsf {s}}$ picks for $\textrm{EU}_p$, then, for any picking strategy ${\mathsf {s}}'$ in ${\mathcal {S}}$,

$$\begin{aligned} \mathsf{Exp}_{p\times \mu }[{\mathfrak {U}}({\mathsf{s}})]\geqslant \mathsf{Exp}_{p\times \mu }[{\mathfrak {U}}({\mathsf{s}}')]. \end{aligned}$$

That is, if ${\mathsf {s}}$ picks for $\textrm{EU}_p$, then ${\mathsf {s}}\in \textrm{EU}_{p\times \mu }({\mathcal {S}})$.

(Recall: ${\mathcal {S}}$ is the set of picking strategies. $\textrm{EU}_{p\times \mu }({\mathcal {S}})$ is the set of those picking strategies that maximize expected utility by the lights of $p\times \mu$, as in Definition 2.2.) This is proved in Appendix C.1.

This shows that expected utility theory is not self-undermining in the way the Allais-permitting decision theories considered in the previous section are self-undermining. Expected utility picking strategies are themselves maximisers of expected utility.

What’s more, they are the only picking strategies which maximise expected utility. Or at least, the picking strategies which maximise expected utility are those that look like an $\textrm{EU}_p$ strategy from *μ *’s perspective.

### Definition 2.4

If ${\mathsf {c}}$ is a choice function and ${\mathsf {s}}$ is a picking strategy, then ${\mathsf {s}}$ *μ *-*surely picks for* ${\mathsf {c}}$ iff $\mu \{D \in {\mathcal {D}}\vert {\mathsf {s}}(D)\in {\mathsf {c}}(D)\}=1$

That is, ${\mathsf {s}}$ *μ *-surely picks for ${\mathsf {c}}$ just in case *μ * is certain you’ll face a decision problem where what ${\mathsf {s}}$ picks is compatible with ${\mathsf {c}}$, i.e., ${\mathsf {s}}(D)\in {\mathsf {c}}(D)$. That is, *μ * is sure that ${\mathsf {s}}$ does not pick an option that ${\mathsf {c}}$ rejects.

### Proposition 2.5

For any *p* and *μ *, if ${\mathsf {s}}$ *μ *-surely picks for $\textrm{EU}_p$, while ${\mathsf {s}}'$ does not, then

$$\begin{aligned} \mathsf{Exp}_{p\times \mu }[{\mathfrak {U}}({\mathsf {s}})] > \mathsf{Exp}_{p\times \mu }[{\mathfrak {U}}({\mathsf {s}}')]. \end{aligned}$$

So, if ${\mathsf {s}}$ does not *μ *-surely pick for $\textrm{EU}_p$, then ${\mathsf {s}}\notin \textrm{EU}_{p\times \mu }({\mathcal {S}})$.

This is proved in Appendix C.1. We thus have that ${\mathsf {s}}\in \textrm{EU}_{p\times \mu }({\mathcal {S}})$ iff ${\mathsf {s}}$ *μ *-surely picks for $\textrm{EU}_p$. It is worth noting that the reasoning that delivers these results only holds when we have assumed that the state of the world is independent of the act chosen.

### Decision-state dependence

As well as assuming act-state independence, we’ve also assumed decision-state independence: that is, we’ve assumed that, from the point of view of your credences over $\Omega \times {\mathcal {D}}$, the decision you face and the state of the world are independent of one another, given by $b=p\times \mu$. But, in fact, analogues of Propositions 2.3 and 2.5 hold even if we don’t assume this.

In any decision problem, we must bring one’s probability *b* up to speed on the problem that you face (by conditionalizing on that information), and then use expected utility theory with this updated credence function to determine what to select.^9^

#### Definition 2.6

If *b* is a probability over $\Omega \times {\mathcal {D}}$, we specify a choice function $\textrm{EU}_{b(\cdot |-)}$ given by $\textrm{EU}_{b(\cdot |-)}(D)=\textrm{EU}_{b(\cdot |D)}(D)$, when this is well-defined. That is,

$$\begin{aligned} \textrm{EU}_{b(\cdot |-)}(D):=\left\{ a\in D\vert \text {for all }a'\in D, \mathsf{Exp}_{b(\cdot |D)}[{\mathfrak {U}}(a)]\geqslant \mathsf{Exp}_{b(\cdot |D)}[{\mathfrak {U}}(a')]\right\} . \end{aligned}$$

$b_{\mathcal {D}}$ is the marginal probability measure on ${\mathcal {D}}$ generated by *b*, that is $b_{\mathcal {D}}(E)=b(\Omega \times E)$ for measurable $E\subseteq {\mathcal {D}}$.

We say that a picking strategy, ${\mathsf {s}}$, $b_{\mathcal {D}}$-*surely picks for* $\textrm{EU}_{b(\cdot |-)}$ iff

$$\begin{aligned}b_{\mathcal {D}}\{D\vert {\mathsf {s}}(D)\in \mathrm{EU}_{b(\cdot |-)}(D)\}=1 \end{aligned}$$

We assume throughout that *b* is countably additive. When *b* has the form $p\times \mu$, then $b_{\mathcal {D}}$ is just *μ * and, for every *D*, *b(· |D)* is just *p*. These are thus generalisations of our original notions of the choice function $\textrm{EU}_p$ and a strategy ${\mathsf {s}}$ *μ *-surely picking for $\textrm{EU}_p$.

We can then show the more general version of Propositions 2.3 and 2.5:

#### Proposition 2.7

${\mathsf {s}}\in \textrm{EU}_b({\mathcal {S}})$ iff ${\mathsf {s}}$ $b_{\mathcal {D}}$-surely picks for $\textrm{EU}_{b(\cdot |-)}$.

This is proved in Appendix C.1.

## Decision theories for imprecise credences

There is another range of decision theories that diverge from expected utility theories: those theories that accommodate ambiguity and imprecision. In the decision theories considered so far, we represent an individual as assigning precise credences to the various states of the world. But some think we do better to model individuals as having imprecise credences instead (Walley, 1991; Bradley, 2016). There are many ways to do this, but one of the most well-known represents an individual’s doxastic state not by a single credence function, which assigns to each state of the world a single numerical measure of their confidence in that state, but by a set of such functions. We call this set your *credal set*. It is a set ${\mathbb {P}}$ of probability measures over the states of the world, *Ω *.^10^

Many decision theories have been proposed for an agent whose uncertain beliefs are represented in this way. We discuss three prominent ones: *Γ *-Maximin, E-Admissibility and Maximality.

### *Γ *-maximin

To illustrate *Γ *-Maximin, consider an example that is often used to motivate it, namely, the Ellsberg paradox (Ellsberg, 1961):

An urn contains 90 balls. You know that 30 of them are red, and the remaining 60 are black and yellow, but you don’t know how many are black and how many are yellow. I am about to draw a ball from the urn.

If the states of the world are *Red* (I draw a red ball), *Black* (I draw black), and *Yellow* (I draw yellow), you might naturally take your credal set to be

$$ \begin{aligned} {\mathbb {P}}= \lbrace {p \vert p(Red ) = {1}/{3}\ \& \ p(Black )+p(Yellow ) = {2}/{3}} \rbrace . \end{aligned}$$

Now consider the following two possible decision problems, $D^{\textrm{Ellsberg}}_1$ and $D^{\textrm{Ellsberg}}_2$:

$$\begin{aligned} \begin{array}{r|ccc} D^{\textrm{Ellsberg}}_1 & Red & Black & Yellow \\ \hline {\mathbb {P}}& {1}/{3} & x& {2}/{3}-x \\ \hline \hline \text {1E} & \text {\pounds10} & \text {\pounds0} & \text {\pounds0} \\ \text {1F} & \text {\pounds1} & \text {\pounds11} & \text {\pounds1} \end{array} \hspace{10mm} \begin{array}{r|ccc} D^{\textrm{Ellsberg}}_2 & Red & Black & Yellow \\ \hline {\mathbb {P}}& {1}/{3} & x& {2}/{3}-x \\ \hline \hline \text {2E} & \text {\pounds11} & \text {\pounds1} & \text {\pounds11} \\ \text {2F} & \text {\pounds0} & \text {\pounds10} & \text {\pounds10} \end{array} \end{aligned}$$

Faced with these decisions, people often report the Ellsberg preferences: they will choose 1E from $D^{\textrm{Ellsberg}}_1$, and 2F from $D^{\textrm{Ellsberg}}_2$.^11^ And indeed that is exactly what *Γ *-Maximin demands. It says that, faced with a particular decision problem, you should pick one of the acts whose minimum expected utility by the lights of the probability functions in ${\mathbb {P}}$ is maximal: in $D^{\textrm{Ellsberg}}_1$, 1E uniquely maximizes minimum expected utility; and in $D^{\textrm{Ellsberg}}_2$, 2F does that.

#### Definition 3.1

(*Γ *-$$\hbox {Maximin}_{\mathbb {P}}$$ ($$\Gamma _{\mathbb {P}}$$))

$$\begin{aligned} \Gamma _{\mathbb {P}}(D) = \left\{ a \in D \mid (\forall a' \in D)\left[ \min _{p \in {\mathbb {P}}} \mathsf{Exp}_p[{\mathfrak {U}}(a')] \leqslant \min _{p \in {\mathbb {P}}} \mathsf{Exp}_p[{\mathfrak {U}}(a)] \right] \right\} \end{aligned}$$

(This should only be applied when these minima exist, e.g., when ${\mathbb {P}}$ is a closed set.)

So ${\mathsf {s}}$ picks for $\Gamma _{\mathbb {P}}$ iff, for every $D\in {\mathcal {D}}$, ${\mathsf {s}}(D)\in \Gamma _{\mathbb {P}}(D)$. In this case, the only picking strategy that picks for $\Gamma _{\mathbb {P}}$ picks 1E from $D^{\textrm{Ellsberg}}_1$ and 2F from $D^{\textrm{Ellsberg}}_2$. We call this strategy ${\mathsf {s}}_{\textrm{Ellsberg}}$; the strategy corresponding to the Ellsberg preferences. Such a strategy is incompatible with expected utility theory: it does not pick for $\textrm{EU}_p$ for any probability *p*.^12^ Indeed, this fact accounts for Ellsberg’s use of the case: like Allais, he wished to provide an example of intuitively rational preferences that could not be captured by expected utility theory.

Now we will use the theory itself to judge picking strategies. To do this, we need to describe the agent’s uncertainty not only over what the world is like, but also over which decision problem she’ll face. Suppose you have precise probabilities over what decision you’ll face, and you think it’s *50%* likely you’ll face $D^{\textrm{Ellsberg}}_1$ and *50%* likely you’ll face $D^{\textrm{Ellsberg}}_2$. So, we represent your uncertainty as a set, ${\mathbb {B}}$, of (higher-order) probabilities over both *Ω * and ${\mathcal {D}}$, each of which makes the state of the world independent of the decision you’ll face. That is, your credal set is ${\mathbb {B}}=\{p\times \mu ^* \vert p\in {\mathbb {P}}\}$, where $\mu ^*$ is this probability over ${\mathcal {D}}$, and ${\mathbb {P}}$ is the credal set as described in the Ellsberg case.

Observe, then, that (i) $\mathsf{Exp}_{\mu ^*}[{\mathfrak {U}}({\mathsf{s}}_{\text {1E,2F}})(\omega )]=5$ for each *ω * in $\lbrace {Red , Black , Yellow } \rbrace$, but (ii) $\mathsf{Exp}_{\mu ^*}[{\mathfrak {U}}({\mathsf {s}}_{\text {1F,2E}})(\omega )]=6$ for each *ω * in $\lbrace {Red , Black , Yellow } \rbrace$. So, for any *p* with $p\times \mu ^*\in {\mathbb {B}}$, $\textrm{EU}_{p\times \mu ^*}{\mathfrak {U}}({\mathsf {s}}_{\text {1E,2F}})=5$ and $\textrm{EU}_{p\times \mu ^*}{\mathfrak {U}}({\mathsf {s}}_{\text {1F,2E}})=6$. And so ${\mathsf {s}}_{\textrm{Ellsberg}}={\mathsf {s}}_{\text {1E,2F}}\notin \Gamma _{{\mathbb {B}}}({\mathcal {S}})$.

This example is closely related to another phenomenon: Dutch book type challenges or paradoxes of sequential choice, which can be constructed against agents on the basis of such examples (Seidenfeld, 2004; Elga, 2010). For instance, we might consider how the agent will choose in $D^{\textrm{Ellsberg}}_1$ and $D^{\textrm{Ellsberg}}_2$, individually, and then combine these choices and observe that the result is dominated—1F-and-2E dominates 1E-and-2F. One response is simply to reject the package principle. However, there is another version of the examples in which they are presented diachronically: first evaluate $D^{\textrm{Ellsberg}}_1$, then $D^{\textrm{Ellsberg}}_2$. And again we can note that 1F-then-2E dominates 1E-then-2F. And we can note that *Γ *-Maximin would still have you choose 2E when faced with $D^{\textrm{Ellsberg}}_2$, even if you know you’ve already chosen 1F when faced with $D^{\textrm{Ellsberg}}_1$. But some will deny that sure loss as a result of decisions at different times indicates irrationality. What this example highlights is the close connection between the analysis of this paper and existing challenges and discussions for these theories. Any such Dutch book or sequential choice challenge can be seen as a particular instance where one is unsure which decision problem you’ll face, taking them each as equally likely, and evaluating strategies. However, it is a slightly different philosophical question.

Moreover, there is a further question of particular interest in our analysis which goes beyond showing the existence of cases of uncertainty for which the decision theory is undermining, as the Ellsberg case does, or using any instance of the sequential choice or Dutch Book challenges. As discussed in Section 1.2.3, we want a general result that says, for a wide class of ways of being uncertain about what decision you’ll face, *Γ *-Maximin is undermining. We provide such a result in Section 4.1.3.

### E-Admissibility and Maximality

Two alternative decision theories are E-Admissibility and Maximality. When coupled with a credal set ${\mathbb {P}}$, E-Admissibility rejects an act *a* from a decision problem *D* when, for any *p* in ${\mathbb {P}}$, there is some *a'* in *D* that *p* expects to do better than *a*. In that case, each *p* in ${\mathbb {P}}$ considers *some* other option to be better than *a*, even though there may be no *single* option they all agree to be better. In constrast, Maximality rejects an act *a* from *D* when there is some *a'* in *D* that every *p* in ${\mathbb {P}}$ considers better than *a*, i.e., when all *p* in ${\mathbb {P}}$ agree on a *single* option that they expect to be better than *a*. If an act is rejected according to Maximality, then it is also rejected according to E-Admissibility, but not vice versa.

#### **Definition 3.2**

$$\begin{aligned} \textrm{EAd}_{\mathbb {P}}(D) = \lbrace {a \in D \vert (\exists p \in {\mathbb {P}})(\forall a' \in D)(\mathsf{Exp}_p[{\mathfrak {U}}(a)]\geqslant \mathsf{Exp}_p[{\mathfrak {U}}(a')])} \rbrace \end{aligned}$$

${\mathsf {s}}$ picks for $\textrm{EAd}_{\mathbb {P}}$ iff for every $D\in {\mathcal {D}}$, ${\mathsf {s}}(D)\in \textrm{EAd}_{\mathbb {P}}(D).$

Note that $a\in \textrm{EAd}_{\mathbb {P}}(D)$ iff there is some $p\in {\mathbb {P}}$ such that $a\in \textrm{EU}_p(D)$. Thus, any picking strategy ${\mathsf {s}}$ that picks for $\textrm{EU}_p$, for some $p\in {\mathbb {P}}$, also picks for $\textrm{EAd}_{\mathbb {P}}$.

#### **Definition 3.3**

$$\begin{aligned} \textrm{Max}_{\mathbb {P}}(D) = \lbrace {a \in D \vert (\forall a'\in D)(\exists p\in {\mathbb {P}})(\mathsf{Exp}_p[{\mathfrak {U}}(a)] \geqslant \mathsf{Exp}_p[{\mathfrak {U}}(a')])} \rbrace \end{aligned}$$

${\mathsf {s}}$ picks for $\textrm{Max}_{\mathbb {P}}$ iff for every $D\in {\mathcal {D}}$, ${\mathsf {s}}(D)\in \textrm{Max}_{\mathbb {P}}(D)$.

We treat E-Admissibility first.

#### E-Admissibility

We want to consider how E-Admissibility judges picking strategies. This depends on your uncertainty over which decision problem you’ll face as well as your uncertainty about the state of the world. We represent your uncertainty over $\Omega \times {\mathcal {D}}$ with a credal set, ${\mathbb {B}}$, given by a set of probability functions, *b*, over $\Omega \times {\mathcal {D}}$.

We can simply apply our notion of E-Admissibility with the credal set ${\mathbb {B}}$ to determine which picking strategies are E-Admissible.

$$\begin{aligned} \textrm{EAd}_{\mathbb {B}}({\mathcal {S}})=\lbrace {{\mathsf {s}}\in {\mathcal {S}}\vert (\exists b \in {\mathbb {B}})(\forall {\mathsf {s}}' \in {\mathcal {S}})(\mathsf{Exp}_b[{\mathfrak {U}}({\mathsf {s}})]\geqslant \mathsf{Exp}_b[{\mathfrak {U}}({\mathsf {s}}')])} \rbrace \end{aligned}$$

That is, ${\mathsf {s}}\in \textrm{EAd}_{\mathbb {B}}({\mathcal {S}})$ iff there is some $b\in {\mathbb {B}}$ such that ${\mathsf {s}}\in \textrm{EU}_b({\mathcal {S}})$.

By Proposition 2.3, for any $b\in {\mathbb {B}}$ that has the form $p\times \mu$, if ${\mathsf {s}}$ picks for $\textrm{EU}_p$ then it is in $\textrm{EU}_{b}({\mathcal {S}})$, and thus, is in $\textrm{EAd}_{\mathbb {B}}({\mathcal {S}})$. Also, when $p\in {\mathbb {P}}$, any ${\mathsf {s}}$ that picks for $\textrm{EU}_p$ also picks for $\textrm{EAd}_{\mathbb {P}}$. We thus typically have some strategy which both picks for $\textrm{EAd}_{\mathbb {P}}$ and is in $\textrm{EAd}_{\mathbb {B}}$, unlike for *Γ *-Maximin.

##### Proposition 3.4

If $p\in {\mathbb {P}}$ and $p\times \mu \in {\mathbb {B}}$, then any picking strategy ${\mathsf {s}}$ that picks for $\textrm{EU}_p$ both picks for $\textrm{EAd}_{\mathbb {P}}$ and is in $\textrm{EAd}_{\mathbb {B}}({\mathcal {S}})$.

If there exists some *p* and *μ * with $p\in {\mathbb {P}}$ and $p\times \mu \in {\mathbb {B}}$, then there are picking strategies that pick for $\textrm{EAd}_{\mathbb {P}}$ and are in $\textrm{EAd}_{\mathbb {B}}({\mathcal {S}})$.

If, for every $p\in {\mathbb {P}}$, there is some *μ * such that $p\times \mu \in {\mathbb {B}}$, then for every $D\in {\mathcal {D}}$ and $a\in \textrm{EAd}_{\mathbb {P}}(D)$, there is some ${\mathsf {s}}$ such that ${\mathsf {s}}(D)=a$ and ${\mathsf {s}}\in \textrm{EAd}_{\mathbb {B}}({\mathcal {S}})$.

This is proved in Appendix C.2.

There are a number of conditions that guarantee the existence of some $p\times \mu \in {\mathbb {B}}$ for any $p\in {\mathbb {P}}$, and thus ensure that every E-Admissible action in a decision problem is part of a picking strategy that is E-Admissible. For example, suppose you have no views whatsoever about which decisions you will face, nor about the evidential value of information about which decisions you will face. In that case, your credal set ${\mathbb {B}}$ over $\Omega \times {\mathcal {D}}$ is given by the *natural extension* of ${\mathbb {P}}$ to this space, which is the largest (least informative) set of probabilities that extend the probabilities in ${\mathbb {P}}$ to $\Omega \times {\mathcal {D}}$. This is sufficient to guarantee that for every $p\in {\mathbb {P}}$ there is some *μ * on ${\mathcal {D}}$ such that $p\times \mu \in {\mathbb {B}}$.

Alternatively, suppose you have a bit of information both about the world and about which decision problem you will face. Your uncertainty about the world is given by the credal set ${\mathbb {P}}$ over *Ω *. Your uncertainty about the which decision you will face is given by the credal set ${\mathbb {M}}$ over ${\mathcal {D}}$. Suppose also that you treat information about which decisions you will face as *irrelevant* to which state of the world you are in.

In the precise setting, irrelevance is a univocal, symmetric notion: for any joint distribution *b* over $\Omega \times {\mathcal {D}}$, ${\mathcal {D}}$ is *stochastically independent* of (and hence irrelevant to) *Ω * according to *b* just in case $b(\omega \in A\vert D\in E)=b(\omega \in A)$ whenever *b(D∈ E)>0*.^13^ But in the imprecise setting, irrelevance fractures into a variety of distinct, not necessarily symmetric notions.^14^

Consider a case where ${\mathbb {P}}$ and ${\mathbb {M}}$ are closed and convex and you treat ${\mathcal {D}}$ as *epistemically irrelevant* to *Ω *, in the sense of Walley (1991). This means roughly that learning information about which decision problem you face does not change your maximum buy price for any “worldly” gamble, i.e., any gamble whose payout depends only on *Ω *. Suppose that ${\mathbb {P}}$, ${\mathbb {M}}$ and this judgment of epistemic irrelevance jointly capture the totality of your views. In that case, your credal set ${\mathbb {B}}$ over $\Omega \times {\mathcal {D}}$ is given by the *irrelevant natural extension* (see de Cooman & Miranda, 2012a, Thm13). This is the largest (least informative) set ${\mathbb {B}}$ of probabilities *b* over $\Omega \times {\mathcal {D}}$ that marginalize to ${\mathbb {P}}$ and ${\mathbb {M}}$ and satisfy the following inequality constraints: for any gamble $g:\Omega \rightarrow {\mathbb {R}}$ and any $B\subseteq {\mathcal {D}}$ with *b(D∈ B)>0*

$$\begin{aligned} \inf \left\{ \mathsf {Exp}_p[g]\vert p\in {\mathbb {P}}\right\} \leqslant \mathsf {Exp}_b[g^+]\leqslant \sup \left\{ \mathsf {Exp}_p[g]\vert p\in {\mathbb {P}}\right\} \end{aligned}$$

and

$$\begin{aligned} \inf \left\{ \mathsf {Exp}_p[g]\vert p\in {\mathbb {P}}\right\} \leqslant \mathsf {Exp}_b[g^+\vert D\in B]\leqslant \sup \left\{ \mathsf {Exp}_p[g]\vert p\in {\mathbb {P}}\right\} , \end{aligned}$$

where $g^+:\Omega \times {\mathcal {D}}\rightarrow {\mathbb {R}}$ is the “cylindrical extension” of *g* defined by $g^+(\omega ,D)=g(\omega )$ for all $\omega \in \Omega$ and $D\in {\mathcal {D}}$. As many authors have noted, individual probabilities *b* in the irrelevant natural extension ${\mathbb {B}}$ will not in general treat ${\mathcal {D}}$ as irrelevant to *Ω * (*cf.* (De Bock, 2019, pp. 96-7)). Nonetheless, ${\mathbb {B}}$ itself will do so, in the sense described above. Moreover, ${\mathbb {B}}$ will contain any *b* that treats ${\mathcal {D}}$ as stochastically independent of *Ω *. This is sufficient to guarantee that the condition of Proposition 3.4 holds.

Rather than treating ${\mathcal {D}}$ as epistemically irrelevant to *Ω *, you might treat ${\mathcal {D}}$ and *Ω * as *completely independent*, in the sense of Seidenfeld (2007); Cozman (2012), i.e., ${\mathcal {D}}$ and *Ω * are stochastically independent according to every $b\in {\mathbb {B}}$. This is a more stringent notion of irrelevance than epistemic irrelevance (and is also symmetric). If ${\mathbb {P}}$ and ${\mathbb {M}}$ capture your opinions about *Ω * and ${\mathcal {D}}$, respectively, you judge ${\mathcal {D}}$ and *Ω * as completely independent, and nothing more (this captures the totality of your views), then your credal set ${\mathbb {B}}$ over $\Omega \times {\mathcal {D}}$ is the largest (least informative) set ${\mathbb {B}}$ of probabilities *b* over $\Omega \times {\mathcal {D}}$ that marginalize to ${\mathbb {P}}$ and ${\mathbb {M}}$ and satisfies complete independence, i.e., ${\mathbb {B}}=\left\{ p\times \mu \vert p\in {\mathbb {P}}, \mu \in {\mathbb {M}}\right\}$. This is also sufficient to guarantee that the condition of Proposition 3.4 holds.

The upshot is that E-Admissibility is not self-undermining in the same way that *Γ *-Maximin is self-undermining. So long as ${\mathbb {P}}$ and ${\mathbb {B}}$ are appropriately related, then there are strategies that pick for it that it does not deem impermissible.

Do we obtain a converse to Proposition 3.4? Are these the only E-Admissible strategies? A strategy is E-Admissible iff there is some $b\in {\mathbb {B}}$ which expects it to be optimal. We might hope to be able to apply Proposition 2.5 to get that it is only these strategies that are E-Admissible. For this, we need to assume that every *b* in ${\mathbb {B}}$ has the form $p\times \mu$, i.e., that you treat ${\mathcal {D}}$ as completely irrelevant to *Ω *:

##### Proposition 3.5

Suppose ${\mathbb {B}}$ makes *Ω * and ${\mathcal {D}}$ completely independent (so every $b\in {\mathbb {B}}$ has the form $p\times \mu$.)

Then, ${\mathsf {s}}\in \textrm{EAd}_{\mathbb {B}}({\mathcal {S}})$ iff there is some $p\times \mu \in {\mathbb {B}}$ such that ${\mathsf {s}}$ *μ *-surely picks for $\textrm{EU}_p$.

This is proved in Appendix C.2.

So the rather strong judgment of complete independence has rather strong implications for your views about picking strategies. The only strategies that are permissible by the lights of E-Admissibility in this case are ones that pick for expected utility theory, i.e., always pick options that maximize *p*-expected utility, for some $p\times \mu \in {\mathbb {B}}$.

For example, in the Ellsberg case (Section 3.1), $\textrm{EAd}_{\mathbb {P}}(D^{\textrm{Ellsberg}}_1)=\;{\text {1E,1F}}$ and $\textrm{EAd}_{\mathbb {P}}(D^{\textrm{Ellsberg}}_2)=\;{\text {2E,2F}}$; so every strategy picks for $\textrm{EAd}_{\mathbb {P}}$. However, the ${\mathsf {s}}_{\text {1E,2F}}$ strategy, which is the empirically observed strategy, does not pick for any $\textrm{EU}_p$: it is not rationalisable by expected utility theory.^15^ If every $b\in {\mathbb {B}}$ has the form $p\times \mu$ with each *μ * giving positive probability to facing both of the decisions in the Ellsberg case, then ${\mathsf {s}}_{\text {1E,2F}}$ also does not *μ *-surely pick for $\textrm{EU}_p$ for any $p\times \mu \in {\mathbb {B}}$; and thus, it is not in $\textrm{EAd}_{\mathbb {B}}({\mathcal {S}})$, despite picking for $\textrm{EAd}_{\mathbb {P}}$. However, there are some strategies which pick for $\textrm{EAd}_{\mathbb {P}}$ and are in $\textrm{EAd}_{\mathbb {B}}({\mathcal {S}})$, namely any strategy for which there is some $p\times \mu \in {\mathbb {B}}$ which *μ *-surely picks for $\textrm{EU}_p$, for example ${\mathsf {s}}_{\text {1E,2E}}$.

There are often picking strategies ${\mathsf {s}}$ that pick for $\textrm{EAd}_{\mathbb {P}}$—for each decision problem *D*, they pick an action from $\textrm{EAd}_{\mathbb {P}}(D)$—but which are rejected by $\textrm{EAd}_{\mathbb {B}}$—that is, they do not lie in $\textrm{EAd}_{\mathbb {B}}({\mathcal {S}})$. This occurs when, for each decision problem *D*, there is some probability function $p\in {\mathbb {P}}$ such that ${\mathsf {s}}(D)\in \textrm{EU}_p(D)$, but where different probability functions rationalise ${\mathsf {s}}$ in different decision problems, and there is no $(p,\mu )\in {\mathbb {B}}$ such that ${\mathsf {s}}$ *μ *-surely picks for $\textrm{EU}_p$.

This will entail that, for every $(p,\mu )\in {\mathbb {B}}$, $\textrm{EAd}_{\mathbb {P}}$ is not *μ *-surely a restriction of $\textrm{EU}_p$, i.e., $\mu ({D\vert \textrm{EAd}_{\mathbb {P}}(D){\setminus }\textrm{EU}_p(D)\ne \varnothing })>0$. Once we move to *probabilistic* picking strategies below, it will turn out that this condition is also sufficient to ensure that no regular probabilistic picking strategy *μ *-surely picks for $\textrm{EU}_p$. However, it is not sufficient to show the existence of the deterministic picking strategies here. To do that, we require that each *μ * recognises the requirement to coordinate.

We can give some sufficient conditions for the existence of strategies that pick for $\textrm{EAd}_{\mathbb {P}}$ but are rejected by $\textrm{EAd}_{\mathbb {B}}$:

##### Proposition 3.6

Suppose ${\mathbb {B}}$ makes *Ω * and ${\mathcal {D}}$ completely independent (so every $b\in {\mathbb {B}}$ has the form $p\times \mu$.)

Suppose there is a selection of pairwise disjoint events $E_q\subseteq {\mathcal {D}}$, one for each $q\in {\mathbb {P}}$ (some of which may be empty), such that for all $p\times \mu \in {\mathbb {B}}$,

$$\begin{aligned} \mu \left( \bigcup _{q\in {\mathbb {P}}}\{D\in E_q\vert \textrm{EU}_p(D)\cap \textrm{EU}_q(D)=\varnothing \}\right) >0 \end{aligned}$$

Then there is ${\mathsf {s}}$ which picks for $\textrm{EAd}_{\mathbb {P}}$ but which is not in $\textrm{EAd}_{\mathbb {B}}({\mathcal {S}})$.

This is proved in Appendix C.4.

In certain cases, such as the Ellsberg ones, we can straightforwardly verify that this sufficient condition holds. We can also show it holds under some plenitude conditions: if each *μ * assigns strictly positive measure to every collection of take-it-or-leave-it decisions generated by a non-empty open subset of ${\mathcal {A}}$, that is, $\mu ({\{a,0\}\vert a\in V})>0$, for *V* a non-empty open subset of ${\mathcal {A}}$; or if each *μ * assigns strictly positive measure to every non-empty open set of decisions. In such cases, we can select two disjoint $E_1,E_2$ such that for any *p≠ q*, and for *i = 1, 2*, $\mu ({D\in E_i \mid \textrm{EU}_p(D)\cap \textrm{EU}_q(D)=\varnothing })>0$. Then by selecting any distinct $q^*_1,q^*_2$ from ${\mathbb {P}}$, we can see that the conditions of Proposition 3.4 hold. We discuss these in Appendix C.4.

Does this make E-Admissibility self-undermining? Not exactly. But it does mean that, by her own lights, an E-Admissibility decision-maker must pick from her choice set *as if* her credal set represented some true, precise probability which she is simply not in a position to identify. This is close to what Levi (1999) referred to as *imprecise* rather than *indeterminate* probabilities. And it may not sit well with contemporary proponents of E-Admissibility.

On the other hand, one might not see this as a concern for E-Admissibility. It just shows that E-Admissibility sees value in coordinating how you resolve incomparability. Take a simple example.

$$\begin{aligned} \begin{array}{r|cc} D^{\textrm{coord}}_1 & X & \lnot X \\ \hline p & x & 1-x \\ \hline \hline B & \text {\pounds10} & \text {\pounds10} \\ 1C & \text {\pounds0} & \text {\pounds20} \end{array}\hspace{20mm} \begin{array}{r|cc} D^{\textrm{coord}}_2 & X & \lnot X \\ \hline p & x & 1-x \\ \hline \hline B & \text {\pounds10} & \text {\pounds10} \\ 2C & \text {\pounds20} & \text {\pounds0} \end{array} \end{aligned}$$

Any $\textrm{EU}$-maximizer will coordinate their choices in $D^{\textrm{coord}}_1$ and $D^{\textrm{coord}}_2$ in the following sense: assuming their utility is linear in $\mathrm{\pounds}$s, they will choose *B* (reject 1*C*) in $D^{\textrm{coord}}_1$ just in case they choose 2*C* (reject *B*) in $D^{\textrm{coord}}_2$; likewise, they will choose 1*C* (reject *B*) in $D^{\textrm{coord}}_1$ just in case they choose *B* (reject 2*C*) in $D^{\textrm{coord}}_2$.          

Suppose ${\mathbb {P}}= \{p_1, p_2\}$, where $p_1$ expects *B* to be strictly better than 1*C*, while $p_2$ expects *B* to be strictly worse than 2*C*. In that case, $\textrm{EAd}_{\mathbb {P}}(D^{\textrm{coord}}_1)=\;{B,1C}$ and $\textrm{EAd}_{\mathbb {P}}(D^{\textrm{coord}}_2)=\;{B,2C}$. You find both options in both options *incomparable*, i.e., not rejected but also not indifferent, or equally good. Just as each of $p_1$ and $p_2$ coordinates their choices in $D^{\textrm{coord}}_1$ and $D^{\textrm{coord}}_2$, so too does E-Admissibility, advising you to coordinate how you resolve incomparability in a picking strategy. You ought to pick *B* in $D^{\textrm{coord}}_1$ just in case you pick 2*C* in $D^{\textrm{coord}}_2$. Likewise, you ought to pick 1*C* in $D^{\textrm{coord}}_1$ just in case you pick *B* in $D^{\textrm{coord}}_2$.

Now, you might doubt that there is *really* any value in this sort of “modal coordination.” (Recall, you will actually only face one of $D^{\textrm{coord}}_1$ or $D^{\textrm{coord}}_2$. You are not coordinating across time.) But the fact that E-Admissibility *sees* value in coordinating how you resolve incomparability does not render it self-undermining.

Moreover, while E-Admissibility’s lust for coordination does require decision-makers to *pick as if* their probabilities were imprecise rather than indeterminate, in Levi’s sense, this does not mean that they *actually are* imprecise rather than indeterminate. Their credal set need not *actually* represent some true, precise probability which they are unable to identify. This is reflected in their rejection judgments. They often find options genuinely incomparable—not rejected, but not indifferent. No agent with precise probabilities would do so. They are committed to *picking as if* they have some true, precise probability because they value coordination in resolving incomparability. As we said above, it is then an interesting question how far this view then lies from the precise permissivist's view that you must have precise probabilities, and which you have is constrained by your evidence, but typically many are left open to you.

The utility of an action, ${\mathfrak {U}}(a)$, is a gamble on *Ω *. The utility of a picking strategy, in contrast, ${\mathfrak {U}}({\mathsf {s}})$, is a gamble on $\Omega \times {\mathcal {D}}$—a larger, refined sample space. Reasons for rejection that apply to one scale, or level of resolution, might not apply at others. E-Admissibility identifies some reasons for rejection at the scale of picking strategies—reasons grounded in the (putative) value of coordination—that are not reasons for rejection at the scale of actions or options.

#### Maximality

We can also apply the notion of Maximality to determine which picking strategies are Maximal:

$$\begin{aligned} \textrm{Max}_{\mathbb {B}}({\mathcal {S}})=\lbrace {{\mathsf {s}}\in {\mathcal {S}}\vert (\forall {\mathsf {s}}'\in {\mathcal {S}})(\exists b\in {\mathbb {B}})(\mathsf{Exp}_b[{\mathfrak {U}}({\mathsf {s}})]\geqslant \mathsf{Exp}_b[{\mathfrak {U}}({\mathsf {s}}')])} \rbrace \end{aligned}$$

Since Maximality is a more permissive decision theory than E-Admissibility, Proposition 3.4 entails:

##### Proposition 3.7

If $p\in {\mathbb {P}}$ and $p\times \mu \in {\mathbb {B}}$ then any ${\mathsf {s}}$ which picks for $\textrm{EU}_p$ both picks for $\textrm{Max}_{\mathbb {P}}$ and is in $\textrm{Max}_{\mathbb {B}}({\mathcal {S}})$.

If there exists some *p* and *μ * with $p\in {\mathbb {P}}$ and $p\times \mu \in {\mathbb {B}}$, then there are some strategies which pick for $\textrm{Max}_{\mathbb {P}}$ and are in $\textrm{Max}_{\mathbb {B}}({\mathcal {S}})$.

And so, like E-Admissibility, Maximality is not self-undermining in the way that *Γ *-Maximin is self-undermining. There are always strategies that pick for it that it does not deem impermissible, at least as long as such $p\in {\mathbb {P}}$ and $p\times \mu \in {\mathbb {B}}$ exist.

Unlike for E-Admissibility, we do not get the converse result (even under the assumption of complete independence). There can sometimes be some strategies which are not ruled out by Maximality but which nonetheless do not pick for any $\textrm{EU}_p$. This is because a strategy is only ruled out as impermissible if there’s a *single* alternative that is preferable according to *every* $b\in {\mathbb {B}}$. This happens, for example, in the Ellsberg case if one’s probability over which decision problem you think you’ll face is sufficiently imprecise or if it’s precise and pretty confident about which one you will face.

If, however, you think it’s precise and equally likely that you’ll face each of $D^{\textrm{Ellsberg}}_1$ and $D^{\textrm{Ellsberg}}_2$, so your credal set is given by ${\mathbb {B}}=\{p\times \mu ^*\vert p\in {\mathbb {P}}\}$, where $\mu ^*(D^{\textrm{Ellsberg}}_1)=\mu ^*(D^{\textrm{Ellsberg}}_2)=0.5$, then as we observed in Section  3.1, $\mathsf {Exp}_{\mu ^*}[{\mathfrak {U}}({\mathsf {s}}_{\text {1E,2F}})(\omega )]=5$ for each *ω * and $\mathsf{Exp}_{\mu ^*}[{\mathfrak {U}}({\mathsf {s}}_{\text {2E,1F}})(\omega )]=6$; so then for every probability *p*, $\mathsf{Exp}_{p\times \mu ^*}[{\mathfrak {U}}({\mathsf {s}}_{\text {1E,2F}})]=5$ and $\mathsf{Exp}_{p\times \mu ^*}[{\mathfrak {U}}({\mathsf {s}}_{\text {2E,1F}})]=6$, so ${\mathsf {s}}_{\text {1E,2F}}\notin \textrm{Max}_{\mathbb {B}}({\mathcal {S}})$.    

We will be able to show that, if your credences over which decision problem you’ll face are precise, and also have a further property—they “require almost everywhere decisiveness”^16^—then the only strategies that Maximality does not rule as impermissible are the expected utility strategies. We will discuss this and give the details in Section 4.1.3, but we first note that our results so far hold in a more general setting, one where we allow for decision-state dependence.

#### Decision-state dependence

To avoid the various independence assumptions we employed in Section 3.2, we now generalize some of our earlier results to cover the case in which we don’t assume the decision problem you face is independent of the state of the world you’re in, as we did in the precise setting in Section 2.1. To do this, in any decision problem, we must bring ${\mathbb {B}}$ up to speed on the problem that you face (by updating on that information via pointwise conditionalization), and then use the updated credal set to determine which options to reject.

##### **Definition 3.8**

$$\begin{aligned} \textrm{EAd}_{{\mathbb {B}}(\cdot |-)}(D) = \lbrace {a \in D \vert (\exists b \in {\mathbb {B}})(\forall a' \in D)(\mathsf{Exp}_{b(\cdot |D)}[{\mathfrak {U}}(a)]\geqslant \mathsf{Exp}_{b(\cdot |D)}[{\mathfrak {U}}(a')])} \rbrace \end{aligned}$$

${\mathsf {s}}$ picks for $\textrm{EAd}_{{\mathbb {B}}(\cdot |-)}$ iff, for all $D\in {\mathcal {D}}$, ${\mathsf {s}}(D)\in \textrm{EAd}_{{\mathbb {B}}(\cdot |-)}(D)$.

That is, $a\in \textrm{EAd}_{{\mathbb {B}}(\cdot |-)}(D)$ iff there is some $b\in {\mathbb {B}}$ such that $a\in \textrm{EU}_{b(\cdot |-)}(D)$.

Also ${\mathsf {s}}\in \textrm{EAd}_{\mathbb {B}}({\mathcal {S}})$ iff there is some $b\in {\mathbb {B}}$ such that ${\mathsf {s}}\in \textrm{EU}_b({\mathcal {S}})$. We thus have, as a consequence of Proposition 2.7:

##### Proposition 3.9

${\mathsf {s}}\in \textrm{EAd}_{\mathbb {B}}({\mathcal {S}})$ iff for some *b* in ${\mathbb {B}}$, ${\mathsf {s}}$ $b_{\mathcal {D}}$-surely picks for $\textrm{EU}_{b(\cdot |-)}$.

And so:

##### Proposition 3.10

If $b\in {\mathbb {B}}$ and ${\mathsf {s}}$ picks for $\textrm{EU}_{b(\cdot |-)}$, then ${\mathsf {s}}$ both picks for $\textrm{EAd}_{{\mathbb {B}}(\cdot |-)}$ and is in $\textrm{EAd}_{{\mathbb {B}}(\cdot |-)}$.

There thus always exists some strategies which pick for $\textrm{EAd}_{\mathbb {P}}$ and are in $\textrm{EAd}_{{\mathbb {B}}(\cdot |-)}$.

For every $D\in {\mathcal {D}}$ and $a\in \textrm{EAd}_{{\mathbb {B}}(\cdot |-)}(D)$, there is some ${\mathsf {s}}$ such that ${\mathsf {s}}(D)=a$ and ${\mathsf {s}}\in \textrm{EAd}_{\mathbb {B}}({\mathcal {S}})$.

These are both proved in Appendix C.2.

While some of its picking strategies are not ruled out, often some will be ruled out.

##### Proposition 3.11

Suppose there is a selection of pairwise disjoint events $E_{b'}\subseteq {\mathcal {D}}$, one for each $b'\in {\mathbb {B}}$ (some of which may be empty), such that for all $b\in {\mathbb {B}}$,

$$\begin{aligned} b_{\mathcal {D}}\left( \bigcup _{b'\in {\mathbb {B}}}\{D\in E_{b'}\vert \textrm{EU}_{b(\cdot |-)}(D)\cap \textrm{EU}_{b'(\cdot |-)}(D)=\varnothing \}\right) >0. \end{aligned}$$

Then there is ${\mathsf {s}}$ that picks for $\textrm{EAd}_{{\mathbb {B}}(\cdot |-)}$ but which is not in $\textrm{EAd}_{{\mathbb {B}}}({\mathcal {S}})$.

This is proved in Appendix C.4.

Proposition 3.11 provides one example of a “richness condition” on the class of $b_{\mathcal {D}}$ that guarantees that they assign positive probability to a “sufficiently inclusive” set of decision problems that we can find a strategy ${\mathsf {s}}$ that picks for $\textrm{EAd}_{{\mathbb {B}}(\cdot |-)}$ but which is not in $\textrm{EAd}_{{\mathbb {B}}}({\mathcal {S}})$. But it is by no means the only one. For example, let ${\mathsf {c}}$ be any choice function (e.g., $\textrm{EAd}_{{\mathbb {B}}(\cdot |-)}, \textrm{Max}_{{\mathbb {B}}(\cdot |-)}$, etc.). Suppose that for any $b\in {\mathbb {B}}$, $b_{\mathcal {D}}$ assigns positive probability to the set of decisions where ${\mathsf {c}}$ leaves open options that *b* rejects, i.e. $b_{\mathcal {D}}(U_b)>0$ where $U_b:=\bigl \{D\in {\mathcal {D}}\vert {\mathsf {c}}(D)\not \subseteq \textrm{EU}_{b(\cdot |-)}(D)\bigr \}$. Suppose further that there is some measurable statistic $r:{\mathcal {D}}\rightarrow [0,1)$ (think: a gradable property of a decision problem), and that for any $b\in {\mathbb {B}}$ and any open interval *I⊂ [0,1)*, $b_{\mathcal {D}}\bigl (\,D\in U_b\vert r({\mathsf {c}}(D))\in I \bigr )>0$ (roughly: $b_{\mathcal {D}}$ sees a “sufficiently inclusive” subset of $U_b$ that it thinks $r({\mathsf {c}}(D))$ spans [0, 1) on $U_b$). This “richness condition” also guarantees that we can find a strategy ${\mathsf {s}}$ that picks for ${\mathsf {c}}$ but does not $b_{\mathcal {D}}$-surely pick for $\textrm{EU}_{b(\cdot |-)}$ for any $b\in {\mathbb {B}}$. By Proposition 3.9, then, ${\mathsf {s}}$ is not in $\textrm{EAd}_{\mathbb {B}}({\mathcal {S}})$.

##### Proposition 3.12

Suppose that for any $b\in {\mathbb {B}}$, $b_{\mathcal {D}}(U_b)>0$ where

$$\begin{aligned} U_b:=\bigl \{D\in {\mathcal {D}}\vert {\mathsf {c}}(D)\not \subseteq \textrm{EU}_{b(\cdot |-)}(D)\bigr \}. \end{aligned}$$

Suppose further that there is some measurable statistic $r:{\mathcal {D}}\rightarrow [0,1)$ such that for any $b\in {\mathbb {B}}$ and any open interval *I⊂ [0,1)*,

$$\begin{aligned} b_{\mathcal {D}}\bigl (\,D\in U_b\vert r({\mathsf {c}}(D))\in I \bigr )>0. \end{aligned}$$

Then there is ${\mathsf {s}}$ which picks for ${\mathsf {c}}$ but for no $b\in {\mathbb {B}}$ does it $b_{\mathcal {D}}$-surely pick for $\textrm{EU}_{b(\cdot |-)}$.

This is proved in Appendix C.4.

As an immediate corollary of Proposition 3.9 and Proposition 3.12 we have:

##### Corollary 3.13

Suppose that for any $b\in {\mathbb {B}}$, $b_{\mathcal {D}}(U_b)>0$ where

$$\begin{aligned} U_b:=\bigl \{D\in {\mathcal {D}}\vert \textrm{EAd}_{{\mathbb {B}}(\cdot |-)}(D)\not \subseteq \textrm{EU}_{b(\cdot |-)}(D)\bigr \}. \end{aligned}$$

Suppose further that there is some measurable statistic $r:{\mathcal {D}}\rightarrow [0,1)$ such that for any $b\in {\mathbb {B}}$ and any open interval *I⊂ [0,1)*,

$$\begin{aligned} b_{\mathcal {D}}\bigl (\,D\in U_b\vert r(\textrm{EAd}_{{\mathbb {B}}(\cdot |-)}(D))\in I \bigr )>0. \end{aligned}$$

Then there is ${\mathsf {s}}$ which picks for $\textrm{EAd}_{{\mathbb {B}}(\cdot |-)}$ but which is not in $\textrm{EAd}_{{\mathbb {B}}}({\mathcal {S}})$.

For Maximality, we can define:

##### **Definition 3.14**

$$\begin{aligned} \textrm{Max}_{{\mathbb {B}}(\cdot |-)}(D) = \lbrace {a \in D \vert (\forall a' \in D)(\exists p\in {\mathbb {P}})(\mathsf{Exp}_{b(\cdot |D)}[{\mathfrak {U}}(a)]\geqslant \mathsf{Exp}_{b(\cdot |D)}[{\mathfrak {U}}(a')])} \rbrace \end{aligned}$$

Since ${\mathsf {s}}\in \textrm{EAd}_{\mathbb {B}}({\mathcal {S}})$ implies ${\mathsf {s}}\in \textrm{Max}_{\mathbb {B}}({\mathcal {S}})$, we obtain, as a consequence of Proposition 3.9:

##### Proposition 3.15

If for some *b* in ${\mathbb {B}}$, ${\mathsf {s}}$ $b_{\mathcal {D}}$-surely picks for $\textrm{EU}_{b(\cdot |-)}$, then ${\mathsf {s}}\in \textrm{Max}_{\mathbb {B}}({\mathcal {S}})$.

This is proved in Appendix C.3. And thus, there is always some ${\mathsf {s}}$ which picks for $\textrm{Max}_{{\mathbb {B}}(\cdot |-)}$ which is itself not rejected according to $\textrm{Max}_{\mathbb {B}}({\mathcal {S}})$. However, again, we do not have an analogue of Proposition 3.9 unless we impose additional particular restrictions on ${\mathbb {B}}$ (Sect. 4.1.3).

## The utility of using a decision theory

Up to this point, we have asked how a decision theory evaluates the picking strategies that pick for the choice function to which that decision theory gives rise. This is one way to answer the question whether the decision theory undermines its own recommendations, and we’ve seen that Allais-permitting decision theories fare poorly, as does *Γ *-Maximin both of which rule out as impermissible the strategy that they require; while E-Admissibility and Maximality fare better, as some compatible strategies are evaluated as acceptable (although not all).

However, other approaches are available too. We are interested in judging a decision theory as a means to your ends, and we have been using the proposed decision theory itself to do the judging, for it is, after all, a theory of which means to your ends are rational. Judging picking strategies that pick for the choice function that a decision theory produces furnishes us with a straightforward approach to this question, because they determine what the outcomes are: given a decision problem and a state of the world, the utility of a picking strategy is the utility, at that state of the world, of the act it picks from the decision problem. Since a decision theory doesn’t always give definitive guidance on which act to pick when faced with a decision, we considered various strategies compatible with its recommendations; in our terminology, the strategies that pick for it. How else might we evaluate what a decision theory will lead you to do when there are various strategies it leaves open?

We propose that you might have a precise probability over the acts the decision theory deems permissible—what we’ll call a probabilistic picking strategy—and you might take the utility of this probabilistic picking strategy at a state of the world to be its expected utility at that world. There are two reasons you might think this is the right way to evaluate a decision theory:

Firstly, you might think that, once your decision theory gives you its choice set, you will pick by applying some randomisation method, such as tossing a coin or rolling a die. Perhaps you think we are freely selecting amongst various randomisation methods as well as the choice functions to which your decision theory gives rise, or perhaps you think that, when selecting a choice function, it simply comes with a specified randomisation method.

Secondly, you might think that, once your decision theory gives its choice set, you don’t know what happens next, except that, in the end, you do in fact pick a particular act from that set. We then want to represent your uncertainty about how you’ll end up picking when you’ve adopted a particular decision theory whose choice set is not a singleton. And it might just be that your uncertainty over how you’ll pick is best represented by a precise probability. (In Section 4.2.1, we will extend this to the case where your uncertainty over how you’ll pick is imprecise.)

Before discussing some alternatives, we will now show that under either of these ways of thinking about judging the outcomes of adopting a decision theory, all our previous claims carry over, and in fact in some cases even get worse since the strategies that align with expected utility theory arise from extremal picking strategies which we might want to rule out under this way of thinking.

### Probabilistic picking strategies

We begin by extending our definitions:

#### Definition 4.1

- A *probabilistic picking strategy* ${\mathsf {n}}$, specifies, for each decision problem, $D\in {\mathcal {D}}$, a probability function ${\mathsf {n}}_D$ over *D*, i.e., over the acts available in the decision problem *D*.
- For a choice function ${\mathsf {c}}$, ${\mathsf {n}}$ *picks for* ${\mathsf {c}}$ iff for all $D\in {\mathcal {D}}$, ${\mathsf {n}}_D({\mathsf {c}}(D))=1$, i.e., it is certain that what it picks will be compatible with ${\mathsf {c}}$’s recommendations.
- For a choice function ${\mathsf {c}}$, ${\mathsf {n}}$ *μ *-*surely picks for* ${\mathsf {c}}$, if $\mu \{D\vert {\mathsf {n}}_D({\mathsf {c}}(D))=1\}=1$.

Observe that in the special case where ${\mathsf {n}}$ is extremal—that is, when for every *D* it assigns all its probabilistic weight to an individual member of *D*—then we recover our original notion of a picking strategy. We will call these the *deterministic* picking strategies.

We add a further definition:

#### Definition 4.2

For a choice function ${\mathsf {c}}$, ${\mathsf {n}}$ *is regular for* ${\mathsf {c}}$, if it picks for ${\mathsf {c}}$ and for every $D\in {\mathcal {D}}$ and $a\in {\mathsf {c}}(D)$, ${\mathsf {n}}_D(a)>0$.

For a deterministic picking strategy, ${\mathsf {s}}$, we simply took its utility to be the utility of the act it requires you to pick: given a state of the world *ω * and a decision problem *D*, ${\mathfrak {U}}({\mathsf {s}})(\omega ,D):={\mathfrak {U}}({\mathsf {s}}(D))(\omega )$, the utility of the act ${\mathsf {s}}(D)$ at *ω *. For ${\mathsf {n}}$, we take its utility to be the *expected* utility of the act it lead you to pick: given a state of the world *ω * and a decision problem *D*,

$$\begin{aligned} {\mathfrak {U}}({\mathsf {n}})(\omega ,D):=\sum _{a \in D} {\mathsf {n}}_D(a){\mathfrak {U}}(a)(\omega ). \end{aligned}$$

We have thus far been assuming that decision problems *D* are non-empty finite sets of acts. If we were to allow *D* to be infinite (although compact), then we should have ${\mathfrak {U}}({\mathsf {n}})(\omega ,D):=\int _{D} {\mathfrak {U}}(a)(\omega )\; {\mathsf {n}}_D(\!\textrm{d}a)$.

In the next few sections, we note how our earlier results concerning deterministic picking strategies generalize to probabilistic picking strategies.

We will judge whether a given decision theory considers a probabilistic picking strategy ${\mathsf {n}}$ to be impermissible. This will depend on the range of alternatives available. That is, we will be judging whether ${\mathsf {n}}$ is an impermissible picking strategy from a set of picking strategies, ${\mathcal {N}}$. There are various natural proposals for what ${\mathcal {N}}$ contains, depending on one’s interpretation and applications of our results. When ${\mathcal {N}}$ consists just of extremal picking strategies, it is equivalent to the set of deterministic picking strategies, ${\mathcal {S}}$, which we considered in the first half of the paper. If you think you get to pick by randomisation, and can select any randomisation process, then ${\mathcal {N}}$ will be the collection of all probabilistic picking strategies. If you think we are just evaluating choice functions, and each one just comes along with a single randomisation process (for example, a uniform distribution over its choice set), then ${\mathcal {N}}$ will have a particular ${\mathsf {n}}^{\mathsf {c}}$ for each ${\mathsf {c}}$, where ${\mathsf {n}}^{\mathsf {c}}$ picks for ${\mathsf {c}}$. If instead you are just uncertain over how you’ll pick when using a choice function ${\mathsf {c}}$, and assume that this is a matter governed by a precise probability, then again we’ll have a ${\mathsf {n}}^{\mathsf {c}}$ representing your probabilistic uncertainty over how you’ll pick once you’ve selected a choice function ${\mathsf {c}}$ and are faced with a decision *D*.

#### Expected Utility Theory

Our results all transpose to the probabilistic setting for any choice of ${\mathcal {N}}$ with a particular feature: it contains some ${\mathsf {n}}^{\mathsf {c}}$, for each relevant choice function $\textrm{EU}_p$ or $\textrm{EU}_{b(\cdot |-)}$.

##### Definition 4.3

A set of probabilistic picking strategies, ${\mathcal {N}}$:

- ${\mathcal {N}}$ is $\textrm{EU}$-*complete* if, for every probability *p* over *Ω *, there is some ${\mathsf {n}}$ in ${\mathcal {N}}$ such that ${\mathsf {n}}$ picks for $\textrm{EU}_p$.
- ${\mathcal {N}}$ is *conditional*-$\textrm{EU}$-*complete* if, for every probability *b* over $\Omega \times {\mathcal {D}}$, there is some ${\mathsf {n}}$ in ${\mathcal {N}}$ such that ${\mathsf {n}}$ picks for $\textrm{EU}_{b(\cdot |-)}$.
- ${\mathcal {N}}$ is *deterministically full* if ${\mathcal {N}}\supseteq {\mathcal {S}}$, that is, if ${\mathcal {N}}$ contains all the deterministic picking strategies.

If it contains some ${\mathsf {n}}^{\mathsf {c}}$ for every possible choice function then ${\mathcal {N}}$ is deterministically full.

##### Proposition 4.4

${\mathcal {N}}$ is deterministically full *ımplies* ${\mathcal {N}}$ is conditional-$\textrm{EU}$-complete *ımplies* ${\mathcal {N}}$ is $\textrm{EU}$-complete.

These conditions would fail if, for example, our agents were bounded in a particular way that would render them unable to act according to a specified choice function; but we are assuming that isn’t our case.

In fact, our results only need ${\mathcal {N}}$ is $\textrm{EU}$-complete for ${\mathbb {B}}$ which we can define as: for every $b\in {\mathbb {B}}$, there is some ${\mathsf {n}}$ in ${\mathcal {N}}$ such that ${\mathsf {n}}$ $b_{\mathcal {D}}$-surely picks for $\textrm{EU}_{b(\cdot |-)}$, but in the main body of the paper we will state the results with the more general restrictions on ${\mathcal {N}}$.

Propositions 2.3 and 2.5 extend to this setting. Suppose *p* is a probability over *Ω * and *μ * is a probability measure over ${\mathcal {D}}$.

##### Proposition 4.5

If ${\mathcal {N}}$ is $\textrm{EU}$-complete, we have:

$$\begin{aligned} {\mathsf {n}}\in \textrm{EU}_{p\times \mu }({\mathcal {N}}) \Leftrightarrow {\mathsf {n}}\; \mu {-}\text {surely \;picks\; for }\;\textrm{EU}_p. \end{aligned}$$

If ${\mathcal {N}}$ is conditional-$\textrm{EU}$-complete, then

$$\begin{aligned} {\mathsf {n}}\in \textrm{EU}_{b}({\mathcal {N}}) \Leftrightarrow {\mathsf {n}}\;b_\mathcal{D}{-}\text{surely \;picks \;for}\; {\mathrm{EU}}_{b(\cdot | - )}. \end{aligned}$$

This is proved in Appendix C.1.

#### E-Admissibility

Propositions 3.4, 3.6, 3.9 and 3.10 also generalise to the probabilistic picking strategy setting.

Since a probabilistic picking strategy is in $\textrm{EAd}_{\mathbb {B}}$ iff, for some *b* in ${\mathbb {B}}$, it is in $\textrm{EU}_b$, we get as an immediate consequence of Proposition 4.5:

##### Proposition 4.6

If for some $p\times \mu \in {\mathbb {B}}$, ${\mathsf {n}}$ *μ *-surely picks for $\textrm{EU}_p$, then ${\mathsf {n}}\in \textrm{EAd}_{\mathbb {B}}({\mathcal {N}})$. More generally, if for some $b\in {\mathbb {B}}$, ${\mathsf {n}}$ $b_{\mathcal {D}}$-surely picks for $\textrm{EU}_{b(\cdot |-)}$, then ${\mathsf {n}}\in \textrm{EAd}_{\mathbb {B}}({\mathcal {N}})$.

Moreover, these are the only members of $\textrm{EAd}_{\mathbb {B}}$, at least assuming that ${\mathcal {N}}$ is conditional-$\textrm{EU}$-complete:

$$\begin{aligned} {\mathsf {n}}\in \textrm{EAd}_{\mathbb {B}}({\mathcal {N}}) \Leftrightarrow {\text {for \;some }} \;b \;{\text { in }}\;{\mathbb {B}}, {\mathsf {n}}\ b_{\mathcal {D}}{\text {-surely\; picks\; for }}\;\textrm{EU}_{b(\cdot |-)}. \end{aligned}$$

This is proved in Appendix C.2.

##### Proposition 4.7

Suppose that ${\mathcal {N}}$ is conditional-$\textrm{EU}$-complete.

Suppose that for every $b\in {\mathbb {B}}$, $b_{\mathcal {D}}\{D\vert \textrm{EAd}_{{\mathbb {B}}(\cdot \vert-)}(D)\subseteq \textrm{EU}_{b(\cdot \vert-)}\}<1$.

That is, for all $b\in {\mathbb {B}}$, $b_{\mathcal {D}}\left\{D\vert \right.$ there is $\; b'\;\in {\mathbb {B}}$ with $\;\textrm{EU}_{b'(\cdot \vert-)}(D)\not \subseteq \textrm{EU}_{b(\cdot \vert-)}(D)\}>0.$

Then, if ${\mathsf {n}}$ is a regular picking strategy for $\mathrm{EAd}_{{\mathbb {B}}(\cdot \vert-)}$, then ${\mathsf {n}}\notin \textrm{EAd}_{\mathbb {B}}({\mathcal {N}})$.

This is proved in Appendix C.4.

Let’s see this at work. Consider again the Ellsberg setup from Section 3.1. Recall that $\mathrm{EAd}_{\mathbb {P}}(D^{\mathrm{Ellsberg}}_1)= {\text {1E,1F}}$ and $\mathrm{EAd}_{\mathbb {P}}(D^{\mathrm{Ellsberg}}_2)= {\text {2E,2F}}$. Every probability in ${\mathbb {P}}$ rules out at least one of the E-Admissible options as impermissible. For example, any $p{\text{(Black )}}>7{\slash}30$ rules 1E as excluded, i.e., not in $\mathrm{EU}_p$, but there’s some positive chance that ${\mathsf {n}}_{D^{\textrm{Ellsberg}}_1}$ picks 1E, by the assumption that it is regular for $\mathrm{EAd}_{\mathbb {P}}$. Thus, ${\mathsf {n}}$ does not pick for $\mathrm{EU}_p$. It also does not even *μ *-surely pick for $\mathrm{EU}_p$ if we assume that each *μ * assigns positive probability to both $D^{\mathrm{Ellsberg}}_1$ and $D^{\mathrm{Ellsberg}}_2$. It is thus not E-Admissible.

In fact, if ${\mathbb {P}}$ is allowed to be non-convex, we get cases where ${\mathsf {n}}$ will be judged impermissible even when you know what decision problem you’ll face.^17^ Suppose you’re certain you’ll face a decision problem $D = {a_1, a_2}$. So *μ (D) = 1*. And suppose ${\mathbb {P}}= {p_1, p_2}$, where $p_1$ expects $a_1$ to be strictly better than $a_2$, while $p_2$ expects $a_2$ to be strictly better than $a_1$. So E-Admissibility with ${\mathbb {P}}$ says that neither $a_1$ or $a_2$ are rejected. Then for any regular picking strategy for $\textrm{EAd}_{\mathbb {P}}$, ${\mathsf {n}}_D$ will give positive probability to both $a_1$ and $a_2$. $p_1$ doesn’t expect it to be best, and nor does $p_2$. So, by E-Admissibility, ${\mathsf {n}}$ is not rationally permissible.

One motivation for introducing probabilistic picking strategies, and in particular regular probabilistic picking strategies, was to judge an agent’s decision theory as a means to her ends. We wished to give a particular judgement of how good it would be to adopt a given decision theory, rather than simply leaving open a whole range of picking strategies, which represent a range of different ways of implementing that theory. If this is how we are trying to judge E-Admissibility, then E-Admissibility is self-undermining. For example, if one picks amongst the non-rejected options by randomisation, with a regular randomisation device, then E-Admissibility deems it impermissible. The defender of E-Admissibility will argue that one shouldn’t select by randomisation, and also shouldn’t have uncertainty over how one picks in a way which amounts to randomisation. Instead, the defender of E-Admissibility will highlight that it sees value in coordinating how you resolve incomparability. Randomisation, or anything that amounts to that, just won’t do.

The point is a general one. If you try to give any way of scoring, or measuring the utility of choice functions or decision rules at each world and at each decision problem, then you can only avoid being ruled out as impermissible by the lights of E-Admissibility if your rule is equivalent to expected utility theory.

#### Maximality

As in Section 3.2, since Maximality is more permissive than E-Admissibility, all $\textrm{EU}_p$ strategies are evaluated as acceptable according to Maximality. We thus have, as a corollary to Proposition 4.5, and extending Proposition 3.7:

##### Proposition 4.8

If for some $p\times \mu \in {\mathbb {B}}$, ${\mathsf {n}}$ *μ *-surely picks for $\textrm{EU}_{p}$, then ${\mathsf {n}}\in \textrm{Max}_{\mathbb {B}}({\mathcal {N}})$.

More generally, if for some $b\in {\mathbb {B}}$, ${\mathsf {n}}$ $b_{\mathcal {D}}$-surely picks for $\textrm{EU}_{b(\cdot |-)}$, then ${\mathsf {n}}\in \textrm{Max}_{\mathbb {B}}({\mathcal {N}})$.

This is proved in Appendix C.3.

For E-Admissibility, we were able to show that it is only these strategies that are judged acceptable by the decision theory. This result does not immediately apply to Maximality in a similar way because, for a strategy to be deemed impermissible, the various probabilities $b\in {\mathbb {B}}$ have to agree on a particular alternative as better.

However, if we impose an additional restriction on ${\mathbb {B}}$ we can obtain an analogous result: suppose your credence over which decision you’ll face is given by a single, *precise* probability, $\mu ^*$, and that ${\mathbb {B}}$ has the form $\{p\times \mu ^*\vert p\in {\mathbb {P}}\}$. All our results equally apply when ${\mathbb {B}}$ is the convex hull of this, since taking a convex hull doesn’t affect which acts are rejected by Maximality. But, if ${\mathbb {P}}$ is convex, then $\{p\times \mu ^*\vert p\in {\mathbb {P}}\}$ is also convex (since $\mu ^*$ is fixed), and so we do not bother presenting this strengthening.

We will also place a further condition on $\mu ^*$:

##### Definition 4.9

$\mu ^*$ *requires almost everywhere decisiveness* iff for all probabilities *p*,

$$\begin{aligned} \mu ^*\lbrace {D\vert \textrm{EU}_p(D)\text { is a singleton}} \rbrace =1. \end{aligned}$$

That is, for each probability function *p*, $\mu ^*$ is certain you’ll face a decision problem in which only one act maximizes expected utility. That is, the set of decision problems in which there are ties for expected utility has measure 0. For example, fix a proposition and suppose you will face the choice between paying $\mathrm{\pounds} t$ for a bet that pays out $\mathrm{\pounds}1$ if the proposition is true and $\mathrm{\pounds} 0$ if it is false, for unknown *t* in [0, 1]. Then, if $\mu ^*$ is a measure that assigns strictly positive weight to every non-degenerate interval *[x,y] ⊆ [0, 1]*, then $\mu ^*$ requires almost everywhere decisiveness. Whatever your probability in the fixed proposition, the set of decisions of this form in which buying the bet and rejecting the bet have equal expected utility has measure 0 by the lights of $\mu ^*$.    

Then we have the following result.^18^

##### Proposition 4.10

Suppose ${\mathcal {N}}$ is $\textrm{EU}$-complete. Suppose that ${\mathbb {B}}$ has the form $\{p\times \mu ^*\vert p\in {\mathbb {P}}\}$ and $\mu ^*$ requires almost everywhere decisiveness. Then if ${\mathsf {n}}\in \textrm{Max}_{\mathbb {B}}({\mathcal {N}})$, then there is some probability $p\in {\mathcal {P}}$ such that ${\mathsf {n}}$ $\mu ^*$-surely picks for $\textrm{EU}_p$.

This is proved in Appendix D. It follows from a version of Wald’s Complete Class Theorem. Using that, we show that, if ${\mathsf {n}}$ is not in $\textrm{EU}_{p\times \mu ^*}$ for any *p*, then there is some alternative ${\mathsf {n}}'$—in fact, an alternative ${\mathsf {n}}'$ that picks for some $\textrm{EU}_p$—such that $\mathsf{Exp}_{\mu ^*}[{\mathfrak {U}}({\mathsf {n}}')(\omega )]>\mathsf{Exp}_{\mu ^*}[{\mathfrak {U}}({\mathsf {n}})(\omega )]$ for all *ω *, and thus for all probabilistic *p*, $\mathsf{Exp}_p[\mathsf{Exp}_{\mu ^*}[{\mathfrak {U}}({\mathsf {n}}')]]\, > \mathsf{Exp}_p[\mathsf {Exp}_{\mu ^*}[{\mathfrak {U}}({\mathsf {n}})]]$, i.e., $\mathsf{Exp}_{p\times \mu ^*}[{\mathfrak {U}}({\mathsf {n}}')]>\mathsf{Exp}_{p\times \mu ^*}[{\mathfrak {U}}({\mathsf {n}})]$. It follows that ${\mathsf {n}}\notin \textrm{Max}_{\mathbb {B}}({\mathcal {N}})$.

The set ${\mathcal {S}}$ of all deterministic picking strategies discussed in the earlier part of the paper is EU-complete, and so we obtain the result that we hinted at in Sect. 3.2, namely, that if we have ${\mathbb {B}}$ that satisfies the conditions of Proposition 4.10, then it is only $\textrm{EU}_p$ strategies that are in $\textrm{Max}_{\mathbb {B}}({\mathcal {S}})$. Now consider the following corollary of Proposition 3.6:

##### Corollary 4.11

Suppose that ${\mathbb {B}}$ has the form $\{p\times \mu ^*\vert p\in {\mathbb {P}}\}$.

 Suppose there is a selection of pairwise disjoint events $E_q\subseteq {\mathcal {D}}$, one for each $q\in {\mathbb {P}}$ (some of which may be empty), such that for all $p\in {\mathcal {P}}$,

$$\begin{aligned} \mu ^*\left( \bigcup _{q\in {\mathbb {P}}}\{D\in E_q\vert \textrm{EU}_p(D)\cap \textrm{EU}_q(D)=\varnothing \}\right) >0 \end{aligned}$$

Then there is ${\mathsf {s}}$ which picks for $\textrm{EAd}_{\mathbb {P}}$ but which does not $\mu ^*$-surely pick for $\textrm{EU}_p$ for any $p\in {\mathcal {P}}$.

If we have ${\mathbb {B}}$ that satisfies the conditions of Proposition 4.10, we can appeal to this corollary and Proposition 4.10, together with the fact that E-Admissibility is at least as permissive as Maximality, to give sufficient conditions under which there is a deterministic strategy ${\mathsf {s}}$ that picks for $\textrm{Max}_{{\mathbb {P}}}$ but that does not belong to $\textrm{Max}_{\mathbb {B}}({\mathcal {S}})$.

##### Proposition 4.12

Suppose ${\mathcal {N}}$ is $\textrm{EU}$-complete. Suppose that ${\mathbb {B}}$ has the form $\{p\times \mu ^*\vert p\in {\mathbb {P}}\}$ and $\mu ^*$ requires almost everywhere decisiveness.

 Suppose there is a selection of pairwise disjoint events $E_q\subseteq {\mathcal {D}}$, one for each $q\in {\mathbb {P}}$ (some of which may be empty), such that for all $p\in {\mathcal {P}}$,

$$\begin{aligned} \mu ^*\left( \bigcup _{q\in {\mathbb {P}}}\{D\in E_q\vert \textrm{EU}_p(D)\cap \textrm{EU}_q(D)=\varnothing \}\right) >0 \end{aligned}$$

Then there is ${\mathsf {s}}$ which picks for $\textrm{Max}_{\mathbb {P}}$ but which is not in $\textrm{Max}_{\mathbb {B}}({\mathcal {S}})$.

We can also apply the same trick to Proposition 3.12. Consider the following corollary:

##### Corollary 4.13

Suppose that for any $p\in {\mathcal {P}}$, $\mu ^*(U_p)>0$ where

$$\begin{aligned} U_p:=\bigl \{D\in {\mathcal {D}}\vert {\mathsf {c}}(D)\not \subseteq \textrm{EU}_p(D)\bigr \}. \end{aligned}$$

Suppose further that there is some measurable statistic $r:{\mathcal {D}}\rightarrow [0,1)$ such that for any $p\in {\mathcal {P}}$ and any open interval *I⊂ [0,1)*,

$$\begin{aligned} \mu ^*\bigl (\,D\in U_p\vert r({\mathsf {c}}(D))\in I \bigr )>0. \end{aligned}$$

Then there is ${\mathsf {s}}$ which picks for ${\mathsf {c}}$ but which does not $\mu ^*$-surely pick for $\textrm{EU}_p$ for any $p\in {\mathcal {P}}$.

This corollary and Proposition 4.10 give us:

##### Proposition 4.14

Suppose ${\mathcal {N}}$ is $\textrm{EU}$-complete. Suppose that ${\mathbb {B}}$ has the form $\{p\times \mu ^*\vert p\in {\mathbb {P}}\}$ and $\mu ^*$ requires almost everywhere decisiveness.

 Suppose that for any $p\in {\mathcal {P}}$, $\mu ^*(U_p)>0$ where

$$\begin{aligned} U_p:=\bigl \{D\in {\mathcal {D}}\vert \textrm{Max}_{\mathbb {P}}(D)\not \subseteq \textrm{EU}_p(D)\bigr \}. \end{aligned}$$

Suppose further that there is some measurable statistic $r:{\mathcal {D}}\rightarrow [0,1)$ such that for any $p\in {\mathcal {P}}$ and any open interval *I⊂ [0,1)*,

$$\begin{aligned} \mu ^*\bigl (\,D\in U_p\vert r(\textrm{Max}_{\mathbb {P}}(D))\in I \bigr )>0. \end{aligned}$$

Then there is ${\mathsf {s}}$ which picks for $\textrm{Max}_{\mathbb {P}}$ but which is not in $\textrm{Max}_{\mathbb {B}}({\mathcal {S}})$.

There are various other natural richness conditions we might consider, e.g.,

##### Proposition 4.15

For any choice function ${\mathsf {c}}$, if $\mu ^*$ is atomless and for all $p\in {\mathcal {P}}$, $\mu ^*\{D\vert {\mathsf {c}}(D)\subseteq \textrm{EU}_p(D)\}<1$, then there is ${\mathsf {s}}$ which picks for ${\mathsf {c}}$ but for no $p\in {\mathcal {P}}$ does it $\mu ^*$-surely pick for $\textrm{EU}_p$.

This is proved in Appendix D.2.

As an immediate corollary of Proposition 4.10 and Proposition 4.15 we have:

##### Corollary 4.16

Suppose ${\mathcal {N}}$ is $\textrm{EU}$-complete. Suppose that ${\mathbb {B}}$ has the form $\{p\times \mu ^*\vert p\in {\mathbb {P}}\}$ and $\mu ^*$ requires almost everywhere decisiveness.

 If $\mu ^*$ is atomless and for all $p\in {\mathcal {P}}$, $\mu ^*\{D\vert \textrm{Max}_{\mathbb {P}}(D)\subseteq \textrm{EU}_p(D)\}<1$, then there is ${\mathsf {s}}$ which picks for $\textrm{Max}_{\mathbb {P}}$ but which is not in $\textrm{Max}_{\mathbb {B}}({\mathcal {S}})$.

Just as we got a more challenging result for E-Admissibility when we restricted attention to *regular* picking strategies, as these won’t look like $\textrm{EU}_p$ strategies, similarly we get a more challenging result for Maximality when we restrict to regular picking strategies because such regular picking strategies will not $\mu ^*$-surely pick for any $\textrm{EU}_p$, unless Maximality just collapses to $\textrm{EU}_p$ for some *p*, or at least $\mu ^*$-surely does so.

##### Proposition 4.17

Suppose ${\mathcal {N}}$ is $\textrm{EU}$-complete. Suppose that ${\mathbb {B}}$ has the form $\{p\times \mu ^*\vert p\in {\mathbb {P}}\}$ and $\mu ^*$ requires almost everywhere decisiveness. Suppose that for every probability *p*, $\mu ^*\{D\vert \textrm{Max}_{\mathbb {P}}(D)\subseteq \textrm{EU}_p(D)\}< 1$.^19^ Then, if ${\mathsf {n}}$ is a regular picking strategy for $\textrm{Max}_{\mathbb {P}}$ then ${\mathsf {n}}\notin \textrm{Max}_{\mathbb {B}}({\mathcal {N}})$.

This is proved in Appendix D.

The lesson from this result is that you should not pick amongst the options that are not ruled out by applying a (regular) randomisation device; nor should you be uncertain over how you’ll pick in a way which amounts to randomisation; at least when your opinions over which decision problem you’ll face are precise and require almost everywhere decisiveness. Just as with E-Admissibility, the defender of Maximality will argue that this is the right answer. In cases like this, you should coordinate how you resolve incomparability. There are reasons for rejection at the scale of picking strategies—reasons grounded in the (putative) value of coordination—that are not reasons for rejection at the scale of actions or options.

#### *Γ *-Maximin

Since *Γ *-Maximin is a more restrictive theory than Maximality, for a strategy to be *Γ *-Maximin there must be some probability function $p\in {\mathcal {P}}$ for which the strategy $\mu ^*$ surely picks for $\textrm{EU}_p$. As a result we have:

##### Proposition 4.18

Suppose ${\mathcal {N}}$ is $\textrm{EU}$-complete. Suppose that ${\mathbb {B}}$ has the form $\{p\times \mu ^*\vert p\in {\mathbb {P}}\}$ and $\mu ^*$ requires almost everywhere decisiveness. Then if ${\mathsf {n}}\in \Gamma _{\mathbb {B}}({\mathcal {N}})$, there is some probability $p\in {\mathcal {P}}$ where ${\mathsf {n}}$ $\mu ^*$-surely picks for $\textrm{EU}_p$.

 Suppose further that for all $p\in {\mathcal {P}}$, $\mu ^*\bigl \{D\in {\mathcal {D}}\vert \Gamma _{\mathbb {P}}(D)\not \subseteq \textrm{EU}_{p}(D)\bigr \}>0$. Then if ${\mathsf {n}}$ is regular for $\Gamma _{\mathbb {P}}$ then ${\mathsf {n}}\notin \Gamma _{\mathbb {B}}({\mathcal {N}})$.

This is proved in Appendix D.2.

We can also use Corollary 4.13 and Proposition 4.15 to show that there are deterministic strategies ${\mathsf {s}}$ that pick for $\Gamma _{{\mathbb {P}}}$ but do not belong to $\Gamma _{\mathbb {B}}({\mathcal {S}})$.

##### Corollary 4.19

Suppose ${\mathcal {N}}$ is $\textrm{EU}$-complete. Suppose that ${\mathbb {B}}$ has the form $\{p\times \mu ^*\vert p\in {\mathbb {P}}\}$ and $\mu ^*$ requires almost everywhere decisiveness.

Suppose that for any $p\in {\mathcal {P}}$, $\mu ^*(U_p)>0$ where

$$\begin{aligned} U_p:=\bigl \{D\in {\mathcal {D}}\vert \Gamma _{\mathbb {P}}(D)\not \subseteq \textrm{EU}_p(D)\bigr \}. \end{aligned}$$

Suppose further that there is some measurable statistic $r:{\mathcal {D}}\rightarrow [0,1)$ such that for any $p\in {\mathcal {P}}$ and any open interval *I⊂ [0,1)*,

$$\begin{aligned} \mu ^*\bigl (\,D\in U_p\vert r(\Gamma _{\mathbb {P}}(D))\in I \bigr )>0. \end{aligned}$$

Then there is ${\mathsf {s}}$ which picks for $\Gamma _{\mathbb {P}}$ but which is not in $\Gamma _{\mathbb {B}}({\mathcal {S}})$.

Likewise, Proposition 4.15 yields:

##### Corollary 4.20

Suppose ${\mathcal {N}}$ is $\textrm{EU}$-complete. Suppose that ${\mathbb {B}}$ has the form $\{p\times \mu ^*\vert p\in {\mathbb {P}}\}$ and $\mu ^*$ requires almost everywhere decisiveness.

 If $\mu ^*$ is atomless and for all $p\in {\mathcal {P}}$, $\mu ^*\{D\vert \Gamma _{\mathbb {P}}(D)\subseteq \textrm{EU}_p(D)\}<1$, then there is ${\mathsf {s}}$ which picks for $\Gamma _{\mathbb {P}}$ but which is not in $\Gamma _{\mathbb {B}}({\mathcal {S}})$.

Even if a strategy $\mu ^*$ surely picks for some $\textrm{EU}_p$, it may nonetheless be impermissible according to *Γ *-Maximin, as we saw, for example, in the Ellsberg case (Section 3.1) where the only strategy compatible with *Γ *-Maximin was judged impermissible by the theory itself.

#### Uncertainty about decisions

For both these Maximality and *Γ *-Maximin results, we have had to assume something particular about your uncertainty concerning the decision you’ll face: we assume you have precise probabilities over the possible decision problems, and those probabilities are broad enough to ensure that they require almost everywhere decisiveness. It is, however, quite general, applying in a much broader range of cases than particular uncertainty generated from specific cases like the Ellsberg or Allais cases (see Section 1.2.3).

It seems troubling enough that, should you acquire sufficient evidence to become uncertain about the decision problems you’ll face in a way that is represented by precise probabilities, you would have to abandon the decision theory or the picking strategy you’re using.

### Alternatives

#### Imprecise picking strategies

So far, we’ve used decision theories to judge deterministic picking strategies, ${\mathsf {s}}$, and probabilistic picking strategies, ${\mathsf {n}}$. But perhaps the proponent of imprecise probabilities thinks the way you pick is better represented by imprecise probabilities, indeed, a set of probabilistic picking strategies.

Since E-Admissibility requires coordinating, and rejects all strategies except for those equivalent to $\textrm{EU}_p$ strategies, perhaps it is the set of all these $\textrm{EU}_p$ strategies that E-Admissibility recommends. Can we apply E-Admissibility itself to judge this proposal?

To do this, we might extend E-Admissibility so that it judges what we might call imprecise acts, where we represent an imprecise act as a set of acts. So the imprecise acts available in decision problem *D* is any ${\mathbb {A}}\subseteq D$. For instance, the set of all the $\textrm{EU}_p$ strategies is such an imprecise act in the decision problem containing all the possible strategies.

For precise acts, E-Admissibility rejects an act when, for every *p* in ${\mathbb {P}}$, there is some alternative act *a'* that *p* expects to do better. When extending E-Admissibility to judge imprecise acts, we have to ask what it means for *p* to expect an imprecise act ${\mathbb {A}}'$ to do better than ${\mathbb {A}}$.

A first suggestion is to say that *p* expects ${\mathbb {A}}'$ to do better than ${\mathbb {A}}$ when, for every $a\in {\mathbb {A}}$ and $a'\in {\mathbb {A}}'$, $\mathsf{Exp}_p[{\mathfrak {U}}(a')]>\mathsf{Exp}_p[{\mathfrak {U}}(a)]$. This is a very hard condition to meet, so very few imprecise acts will be ruled out as impermissible on this basis. This can already rule as impermissible any imprecise picking strategy each of whose members is a regular picking strategy for $\textrm{EAd}_{\mathbb {P}}$, but it does not deem impermissible the imprecise act consisting of all picking strategies or all $\textrm{EU}_p$ strategies, our motivating idea for imprecise picking strategies.

Alternatively, one might say that *p* expects ${\mathbb {A}}'$ to do better than ${\mathbb {A}}$ when, for every $a\in {\mathbb {A}}$ and $a'\in {\mathbb {A}}'$, $\mathsf{Exp}_p[{\mathfrak {U}}(a')]\geqslant \mathsf{Exp}_p[{\mathfrak {U}}(a)]$, and there is some $a\in {\mathbb {A}}$ such that for all $a'\in {\mathbb {A}}'$, $\mathsf{Exp}_p[{\mathfrak {U}}(a')]>\mathsf{Exp}_p[{\mathfrak {U}}(a)]$.^20^ This condition generates a version of E-Admissibility for imprecise acts which rules as impermissible the set of all picking strategies for $\textrm{EAd}_{\mathbb {P}}$.      

This criterion rules out the imprecise picking strategy that consists of the set of all $\textrm{EU}_p$ strategies, unless they all *μ *-surely pick for $\textrm{EU}_p$ for a single $p \times \mu \in {\mathbb {B}}$. Suppose ${\mathbb {N}}$ is a set of picking strategies, and there is no $p \times \mu$ in ${\mathbb {B}}$ such that all strategies in ${\mathbb {N}}$ *μ *-surely pick for $\textrm{EU}_p$; then each probability $b=p\times \mu$ evaluates the precise picking strategy $\{{\mathsf {n}}^p\}$, where ${\mathsf {n}}^p$ picks for $\textrm{EU}_p$, to be better, in this sense: for every ${\mathsf {n}}\in {\mathbb {N}}$, $\mathsf{Exp}_p[{\mathfrak {U}}({\mathsf {n}})]\leqslant \mathsf {Exp}_p[{\mathfrak {U}}({\mathsf {n}}^p)]$, and there is some ${\mathsf {n}}\in {\mathbb {N}}$ with $\mathsf{Exp}_p[{\mathfrak {U}}({\mathsf {n}})]<\mathsf{Exp}_p[{\mathfrak {U}}({\mathsf {n}}^p)]$.

We should not expect to obtain similar results for Maximality. Consider an imprecise picking strategy consisting of the set of all compatible picking strategies, or even all those which are coordinated and pick for $\textrm{EU}_p$ for some *p* in one’s credal set ${\mathbb {P}}$. Each probability in ${\mathbb {P}}$ agrees that there is a precise picking strategy that is better, but they disagree about which this precise picking strategy is: for which *p* should it pick for $\textrm{EU}_p$? Maximality thus avoids the charge of evaluating the imprecise picking strategy as impermissible. There can be imprecise picking strategies that are Maximal and which do not all pick for a single $\textrm{EU}_p$, unlike for E-Admissibility.

So, whilst this approach is a challenge for E-Admissibility, it is an option for Maximality. If picking strategies may be imprecise acts, represented by sets of precise probabilistic picking strategies, then Maximality sometimes evades the charge of being self-undermining. However, it is hard to see how to implement an imprecise picking strategy. Perhaps one could choose by tossing a coin about whose bias you have imprecise probabilities, or perhaps one should have imprecise probabilistic uncertainty about how one will pick. But to maintain this response, one had better not gain additional information sufficient to make one precise.

#### Utility of a choice function not given by a picking strategy

Perhaps we should specify the utility of a choice function in an alternative way. For example, we might say that ${\mathfrak {U}}({\mathsf {c}},D,\omega )=\sup \{{\mathfrak {U}}(a,\omega )\vert a\in {\mathsf {c}}(D)\}$. If we do this, our results clearly do not hold. In fact, if we measure the utility of a choice function in this way, one should be maximally imprecise in every decision problem, that is, one should set ${\mathsf {c}}(D)=D$. And so, even if we require that ${\mathfrak {U}}({\mathsf {c}},D,\omega )$ is a mixture of ${\mathfrak {U}}(a,\omega )$ for $a\in {\mathsf {c}}(D)$, there are ways to define ${\mathfrak {U}}$ such that our results do not follow.

However, measuring the utility of a choice function in this way would need more justification. Why should it be evaluated this way? Why would this be the right judgement of what utility I will get if I adopt the choice function ${\mathsf {c}}$?

Or we could say that, when there’s an imprecise decision set, one should do something else to make the decision, such as consulting an expert or gathering more evidence (De Bock & De Cooman, 2014). But then why is the possibility of consulting an expert or gathering more evidence not represented as an option in the original decision problem?

## Conclusion

We have asked what happens when we use a decision theory to judge itself, or to judge strategies compatible with its recommendations, and we’ve found significant challenges for a host of theories that diverge from expected utility theory.

In Section 1, we showed that risk-weighted expected utility theory, and other theories that accommodate the Allais preferences, are self-undermining in a particular way: for any such theory, there are particular ways of being uncertain about which decisions you’ll face and a single deterministic picking strategy that chooses in line with the recommendations the decision theory would make were you to face each possible decision you might face, and that strategy is not itself acceptable according to the decision theory. These decision theories undermine their own recommendations; they recommend that you should choose in each decision problem in a way that the theory itself rejects when you’re uncertain which decision problem you’ll face. We generated these examples on the basis of the Allais preferences—and indeed any failure of the Independence Axiom would do. We then noted that we see the same phenomenon if we know we’ll face a binary decision defined over two possible states of the world, and we place a uniform distribution over these different possible decisions; and similarly for a number of beta distributions we might place over them. And so the extent of the self-undermining is reasonably broad, but we don’t have a precise general result that shows how broad it is.

In Sect. 2, we showed that traditional Savage-style expected utility theory does not have the same flaw: it always recommends its own picking strategies.

We then turned to decision theories that accommodate ambiguity and imprecision. In Sect. 3.1, we saw that *Γ *-maximin is self-undermining in the way the Allais-permitting theories were, and we generated the witness to this using the Ellsberg preferences. That is, they can rule out their (only) picking strategy as impermissible.

In Sect. 3.2, we observed that E-Admissibility and Maximility aren’t vulnerable to the same challenge, since they judge some of their picking strategies to be permissible. In the case of E-Admissibility, we noted that the decision theory judges a picking strategy acceptable only if there is some probability function such that the picking strategy is certain to pick an option that maximizes expected utility from the point of view of that probability function; and so E-Admissibility requires picking strategies that coordinate across decisions.

In Sect. 4, we turned from deterministic picking strategies, which select a single option from each decision problem, to probabilistic picking strategies, which place a probability distribution over the options in each decision problem. And we asked the same questions: when are decision theories self-undermining? A probabilistic picking strategy might represent a randomisation process; or it might represent your uncertainty about how you’ll pick when that is governed by a precise probability.

We noted that all our previous results generalise to this setting and, moreover, the situation is worse for the imprecise decision theories: E-Admissibility now judges any regular probabilistic picking strategy that picks for it to be impermissible, since such a strategy no longer looks like an expected utility strategy, at least given some mild assumptions on the set of probabilities that represents your uncertainty. For Maximality, we were able to show something similar, although in this case we need a much stronger assumption: that our decision-maker’s uncertainty over which decision she’ll face is governed by a precise probability, along with a further assumption about how likely it is you’ll face a decision that has more than one option that maximizes expected utility. These considerations highlight that one should not in general pick by randomisation amongst the non-rejected options, or have uncertainty over how you’ll pick in a way that amounts to randomisation. Imprecise decision theories see value in coordination.

We also considered extending the theories so that they might judge imprecise picking strategies, represented as sets of picking strategies, such as the set of all expected utility picking strategies. For instance, we suggested that, since each probability function considers a picking strategy that always picks options that maximize expected utility relative to it to be at least as good as all other picking strategies and better than some, we might say that it prefers its own strategy to the set of all picking strategies, and so E-Admissibility might then rule out as impermissible the set of all picking strategies, since each probability function considers something else better. Thus, under this way of applying E-Admissibility to judge imprecise picking strategies, the only strategies that are not judged impermissible are those which correspond to expected utility for a particular probability.

However, similar considerations do not show that Maximality is self-undermining when we consider imprecise picking strategies.

We also noted that our results would not go through were we to measure the utility of an imprecise choice set in some alternative way. For instance, if we say that the utility of a choice function faced with a decision problem at a state of the world is the supremum of the utilities, at that world, of the options in the decision problem that the choice function doesn’t rule out, then of course one should have a maximally imprecise choice function. But motivating and justifying any such analysis remains an important task, and in any case, the consequence in this case is not desirable.

To summarise: we have found challenges for any of the decision theories we’ve considered that depart from expected utility theory. When we ask a whole range of decision theories how they think one should pick, they pretty systematically recommend picking in accordance with expected utility theory. For some of the theories we considered (REU, *Γ *-maximin), this undermines their own recommendations whenever they don’t collapse into the recommendations of expected utility theory. For others (E-Admissibility, Maximality), the situation is less clear, as picking in accordance with expected utility theory is compatible with the theory and choice-worthy according to the theory, however other picking strategies are deemed impermissible, and all regular probabilistic picking strategies are deemed impermissible. These theories thus have to accept a deep-seated value for coordinating how to resolve incomparability across possible decision problems. What *is* clear is that decision theorists must face the question of how to pick head on. 

## Supplementary Information

Below is the link to the electronic supplementary material.

Supplementary file1 (NB 41 KB)

## A Measure theoretic considerations

Throughout the paper, we ignored the question of measurability. We have considered various probability measures: *p* a probability on *Ω *, *μ * a probability on ${\mathcal {D}}$, and *b* a probability on $\Omega \times {\mathcal {D}}$. To make this precise, we should fix the *σ *-algebras on each of these spaces.

Since *Ω * is finite, we can equip it with the discrete *σ *-algebra, ${\mathcal {F}}=\wp (\Omega )$, so that every subset of *Ω * is measurable.

${\mathcal {D}}$ is defined as finite subsets of the set of acts ${\mathcal {A}}$, with ${\mathcal {A}}$ simply being an arbitrary non-empty set. The *σ *-algebra we generate will be defined relative to a utility function ${\mathfrak {U}}:{\mathcal {A}}\rightarrow {\mathbb {R}}^\Omega \cong {\mathbb {R}}^n$. Recall that ${\mathfrak {U}}$ is bounded, so in fact ${\mathfrak {U}}:{\mathcal {A}}\rightarrow [l,h]^\Omega \cong [l,h]^n$.

We define a metric on ${\mathcal {A}}$ induced by ${\mathbb {R}}^n$, that is $d(a,a'):=d({\mathfrak {U}}(a),{\mathfrak {U}}(a'))$, with the Euclidean metric. This then defines the topology on ${\mathcal {A}}$ and the associated Borel *σ *-algebra.

In fact, if there are some *a,a'* that have the same utility profile, ${\mathfrak {U}}(a)={\mathfrak {U}}(a')$, then *d(a,a')=0*, so it is actually a pseudo-metric. Any measurable notions will treat such acts equivalently and it is Borel isomorphic to the structure which identifies any two such acts.

We can then use the Hausdorff metric on ${\mathcal {D}}$ to obtain our topology and the associated Borel *σ *-algebra.

$$\begin{aligned} d(D, D') = \max \Bigl \{ \sup _{a \in D}\inf _{a' \in D'}d(a,a'), \;\sup _{a' \in D'}\inf _{a \in D}d(a,a') \Bigr \}. \end{aligned}$$

We will throughout additionally assume that ${\mathfrak {U}}({\mathcal {A}})$ is a Borel subset of ${\mathbb {R}}^n$, then both ${\mathcal {A}}$ and ${\mathcal {D}}$ are standard Borel spaces.

We then require *p* a probability on $(\Omega ,{\mathcal {F}})$, *μ * a probability on $({\mathcal {D}},\Sigma )$, and *b* a probability on $(\Omega \times {\mathcal {D}},{\mathcal {F}}\otimes \Sigma )$.

All the results in this paper are restricted to *measurable* picking strategies, either of the deterministic form, ${\mathsf {s}}:{\mathcal {D}}\rightarrow {\mathcal {A}}$, or measurable probabilistic picking strategies ${\mathsf {n}}:{\mathcal {D}}\rightarrow \Delta ({\mathcal {A}})$. This ensures that the induced payoffs, ${\mathfrak {U}}({\mathsf {s}}),{\mathfrak {U}}({\mathsf {n}}):\Omega \times {\mathcal {D}}\rightarrow {\mathbb {R}}$, are measurable random variables with respect to $(\Omega \times {\mathcal {D}},{\mathcal {F}}\otimes \Sigma )$ (recalling that ${\mathfrak {U}}({\mathsf {n}})(\omega ,D):=\mathsf {Exp}_{{\mathsf {n}}_D}[{\mathfrak {U}}(\cdot )(\omega )]$).

## B Decision-State Dependence

When we have decision-state dependence, we made use of the idea of conditional probabilities. For *b*, a probability measure on $(\Omega \times {\mathcal {D}},{\mathcal {F}}\otimes \Sigma )$, we assumed we had appropriate conditional probability measures, *b(· | D)*, on *Ω *, defined for $b_{\mathcal {D}}$-almost every *D*. Since $(\Omega ,{\mathcal {F}})$ is standard Borel, this follows immediately from the Conditional Distribution Disintegration Theorem (Kallenberg, 2021, Theorem 8.5), which guarantees that *b(· |D)* is defined and unique for $b_{\mathcal {D}}$-almost every *D*, and that for every $X\in L^2(b)$ and $b_{\mathcal {D}}$-almost every *D*:

$$\begin{aligned}&\mathsf{Exp}_{b}[X | D] =\int _\Omega X(\omega ,D)b(\!\textrm{d}\omega | D)=\sum _{\omega \in \Omega } X(\omega ,D)b(\omega |D). \end{aligned}$$

where *σ (D)* is the *σ *-field generated by *D*, the conditional expectation, $\mathsf{Exp}_{b}[X | \sigma (D)]$, is defined as the orthogonal projection of *X* onto the linear subspace of *σ (D)*-measurable random variables, and $\mathsf{Exp}_{b}[X | D]$ is the value of this random variable at *D*. (This extends by continuity to $X\in L^1(b)$.)

By the tower property of conditional expectation (Kallenberg, 2021, Theorem 8.1) we have $\mathsf{Exp}_b[X]=\mathsf{Exp}_{b}[\mathsf{Exp}_{b}[X | D]]$, which implies

$$\begin{aligned} \mathsf{Exp}_{b}[\mathsf{Exp}_{b}[X | D]]&=\int _{\Omega \times {\mathcal {D}}} \mathsf{Exp}_{b}[X | D] b(\!\textrm{d}\omega , \!\textrm{d}D)\\&=\int _{{\mathcal {D}}} \mathsf{Exp}_{b}[X | D] b_{\mathcal {D}}(\!\textrm{d}D)\\&=\int _{{\mathcal {D}}} \left[ \sum _{\omega \in \Omega } X(\omega ,D)b(\omega |D)\right] b_{\mathcal {D}}(\!\textrm{d}D)\\&=\mathsf{Exp}_{b_{\mathcal {D}}}[\mathsf{Exp}_{b(\cdot |D)}[X(\cdot , D)]] \end{aligned}$$

Henceforth we write $\mathsf{Exp}_{b_{\mathcal {D}}}[\mathsf{Exp}_{b(\cdot |D)}[X]]$ as shorthand for $\mathsf{Exp}_{b_{\mathcal {D}}}[\mathsf{Exp}_{b(\cdot |D)}[X(\cdot , D)]]$. So $\mathsf{Exp}_b[X]=\mathsf{Exp}_{b_{\mathcal {D}}}[\mathsf{Exp}_{b(\cdot |D)}[X]]$.

When $b=p\times \mu$, then $b_{\mathcal {D}}=\mu$ and *b(· |D)=p* for *μ *-almost every *D*. So $\mathsf{Exp}_{p\times \mu }[X]=\mathsf{Exp}_{\mu }[\mathsf{Exp}_{p}[X]]$.

### B.1 $\textrm{EU}$-completeness and measurability

With the clarification that we restrict to *measurable* selection functions, ${\mathcal {N}}$ being $\textrm{EU}$-complete is taken to mean that for every probability *p* over *Ω * there is some ${\mathsf {n}}\in {\mathcal {N}}$ which picks for $\textrm{EU}_p$.

#### Lemma B.1

For every probability *p* there is a measurable ${\mathsf {s}}\in {\mathcal {S}}$ which picks for $\textrm{EU}_p$.

#### Proof

We will apply the Measurable Maximum Theorem of Aliprantis and Border (2006, Theorem 18.19).

Define *φ * a measurable correspondence from ${\mathcal {D}}$ to ${\mathcal {A}}$ by *φ (D)=D*. It is weakly measurable, i.e., for *U* open $\subseteq {\mathcal {A}}$, $\{D\vert U\cap D\ne \varnothing \}$ is measurable (in fact, open) in ${\mathcal {D}}$ (Aliprantis & Border, 2006, 3.91). It also takes non-empty compact values by specification of ${\mathcal {D}}$.

$\mathsf{Exp}_p[{\mathfrak {U}}(a)]=\sum _{\omega \in \Omega }p(\omega )\,{\mathfrak {U}}(a)(\omega )$ is a continuous function of *a*. Conceiving of it as a function from ${\mathcal {A}}\times {\mathcal {D}}\rightarrow {\mathbb {R}}$ which doesn’t depend on *D*, it is trivially measurable as a function of *D*. It is thus a Carathéodory function and we obtain a measurable selector for $\textrm{EU}_p$, as required. *□ *

Similarly for being conditional $\textrm{EU}$-complete: it requires the existence of *measurable* picking strategies for $\textrm{EU}_{b(\cdot |-)}$.

#### Lemma B.2

For every probability *b* over $\Omega \times {\mathcal {D}}$, there is some measurable ${\mathsf {s}}\in {\mathcal {S}}$ which picks for $\textrm{EU}_{b(\cdot |-)}$.

#### Proof

We will apply the Measurable Maximum Theorem of Aliprantis and Border (2006, Theorem 18.19).

As in Lemma B.1, *φ * is a weakly measurable correspondence from ${\mathcal {D}}$ to ${\mathcal {A}}$ which takes non-empty compact values.

Consider $\mathsf{Exp}_{b(\cdot |D)}[{\mathfrak {U}}(a)]$, a function from ${\mathcal {D}}\times {\mathcal {A}}\rightarrow {\mathbb {R}}$.

For fixed *D*, this is $=\sum _{\omega \in \Omega }b(\cdot |D)(\omega ){\mathfrak {U}}(a)(\omega )$, which is linear and continuous in *a*.

We need to show that it is measurable in *D*, for fixed *a*. This holds because $D\mapsto b(\cdot |D)$ is measurable.

It is thus a Carathéodory function and we obtain a measurable selector for $\textrm{EU}_{b(\cdot |-)}$, as required. *□ *

Proposition 4.4 thus follows from these.

## C EU and E-admissibility

### C.1 EU (Propositions 2.3, 2.5, 2.7 and 4.5)

Our first series of results rely on the fact that a picking strategy has maximal expected utility iff it *μ *-surely picks for expected utility theory combined with a particular probability. This core result, stated in Propositiona 2.3 and 2.5, is then extended to cover cases of decision-state dependence (Proposition 2.7) and probabilistic picking strategies (Proposition 4.5).

We will prove the result in generality to cover all these cases.

#### Theorem C.1

(i) If ${\mathsf {n}}$ $b_{\mathcal {D}}$-surely picks for $\textrm{EU}_{b(\cdot |-)}$ then, for any ${\mathsf {n}}'$, $\mathsf{Exp}_b[{\mathfrak {U}}({\mathsf {n}})]\geqslant \mathsf{Exp}_b[{\mathfrak {U}}({\mathsf {n}}')]$  
(ii) If ${\mathsf {n}}$ $b_{\mathcal {D}}$-surely picks for $\textrm{EU}_{b(\cdot |-)}$ and ${\mathsf {n}}'$ does not, then $\mathsf{Exp}_b[{\mathfrak {U}}({\mathsf {n}})]>\mathsf{Exp}_b[{\mathfrak {U}}({\mathsf {n}}')]$.

#### Proof

As in Appendix B, we have $b(\cdot \vert D)$ defined for $b_{\mathcal {D}}$-almost every *D* such that, for every bounded measurable random variable $X:\Omega \times {\mathcal {D}}\rightarrow {\mathbb {R}}$, the law of total expectation holds: $\mathsf{Exp}_b[X]=\mathsf{Exp}_{b_{\mathcal {D}}}[\mathsf{Exp}_{b(\cdot |D)}[X]]$.

${\mathfrak {U}}({\mathsf {n}})$ is a measurable random variable on $\Omega \times {\mathcal {D}}$; and, since we have assumed that ${\mathfrak {U}}$ is bounded above and below, ${\mathfrak {U}}({\mathsf {n}})$ is bounded. And so, $\mathsf{Exp}_{b}[{\mathfrak {U}}({\mathsf {n}})]=\mathsf{Exp}_{b_{\mathcal {D}}}[\mathsf{Exp}_{b(\cdot | D)}[{\mathfrak {U}}({\mathsf {n}})]]$.

Consider any $D^*\in {\mathcal {D}}$ for which $b(\cdot \vert D^*)$ is well defined. Then^21^

$$\begin{aligned} \mathsf{Exp}_{b(\cdot | D^*)}[{\mathfrak {U}}({\mathsf {n}})]=\mathsf{Exp}_{b(\cdot | D)}[\mathsf{Exp}_{{\mathsf {n}}_{D^*}}[{\mathfrak {U}}]]=\mathsf{Exp}_{{\mathsf {n}}_{D^*}}[\mathsf{Exp}_{b(\cdot | D)}[{\mathfrak {U}}]]. \end{aligned}$$

$\mathsf {Exp}_{b(\cdot | D^*)}[{\mathfrak {U}}(a)]$ is maximised, by definition, at any $a\in \textrm{EU}_{b(\cdot |-)}(D^*)$.

Thus, $\mathsf{Exp}_{b(\cdot | D^*)}[{\mathfrak {U}}({\mathsf {n}})]=\mathsf{Exp}_{{\mathsf {n}}_{D^*}}[\mathsf{Exp}_{b(\cdot | D^*)}[{\mathfrak {U}}]]$ is maximised when ${\mathsf {n}}_{D^*}(\textrm{EU}_{b(\cdot |-)}(D^*))=1$.

And so $\mathsf{Exp}_{b}[{\mathfrak {U}}({\mathsf {n}})]=\mathsf{Exp}_{b_{\mathcal {D}}}[\mathsf{Exp}_{b(\cdot | D)}[{\mathfrak {U}}({\mathsf {n}})]]$ is maximised when ${\mathsf {n}}_{D^*}(\textrm{EU}_{b(\cdot |-)}(D))=1$ for $b_{\mathcal {D}}$-almost every *D*. i.e., when ${\mathsf {n}}$ $b_{\mathcal {D}}$-surely picks for $\textrm{EU}_{b(\cdot |-)}$. Our claims follow from this. *□ *

#### Corollary C.2

(i) If ${\mathsf {n}}$ $b_{\mathcal {D}}$-surely picks for $\textrm{EU}_{b(\cdot |-)}$ then ${\mathsf {n}}\in \textrm{EU}_b({\mathcal {N}})$.
(ii) Suppose ${\mathcal {N}}$ contains some ${\mathsf {n}}^b$ that picks for $\textrm{EU}_{b(\cdot |-)}$. Then ${\mathsf {n}}\in \textrm{EU}_b({\mathcal {N}})$ iff ${\mathsf {n}}$ $b_{\mathcal {D}}$-surely picks for $\textrm{EU}_{b(\cdot |-)}$.

#### Proof

(i) is immediate from Theorem C.1.

For (ii), if ${\mathsf {n}}$ does not $b_{\mathcal {D}}$-surely picks for $\textrm{EU}_{b(\cdot |-)}$, then Theorem C.1 implies that $\mathsf{Exp}_b[{\mathfrak {U}}({\mathsf {n}}^b)]>\mathsf{Exp}_b[{\mathfrak {U}}({\mathsf {n}})]$, so ${\mathsf {n}}\notin \textrm{EU}_b({\mathcal {N}})$. *□ *

This suffices to prove Propositions 2.3, 2.5, 2.7 and 4.5.

### C.2 E-Admissibility and EU equivalences (Propositions 3.4, 3.5, 3.9, 3.10 and 4.6)

The results just stated imply many of the results about E-Admissible strategies.

We will again state the results for probabilistic picking strategies, ${\mathsf {n}}$. The results concerning deterministic picking strategies, ${\mathsf {s}}$, are then special cases, recalling that, when ${\mathcal {N}}={\mathcal {S}}$, the same definitions apply.

#### Lemma C.3

(i) ${\mathsf {n}}\in \textrm{EAd}_{\mathbb {B}}({\mathcal {N}})$ iff there is some $b\in {\mathbb {B}}$ such that ${\mathsf {n}}\in \textrm{EU}_b({\mathcal {N}})$.
(ii) If $p\times \mu \in {\mathbb {B}}$ and ${\mathsf {n}}\in \textrm{EU}_{p\times \mu }({\mathcal {N}})$, then ${\mathsf {n}}\in \textrm{EAd}_{\mathbb {B}}({\mathcal {N}})$
(iii) If ${\mathbb {B}}$ makes *Ω * and ${\mathcal {D}}$ completely independent, then ${\mathsf {n}}\in \textrm{EAd}_{\mathbb {B}}({\mathcal {N}})$ iff there is some $p\times \mu \in {\mathbb {B}}$ such that ${\mathsf {n}}\in \textrm{EU}_{p\times \mu }({\mathcal {N}})$.

#### Proof

Immediate from definitions. *□ *

We introduce a new definition that encompasses the notions of $\textrm{EU}$-completeness and conditional-$\textrm{EU}$-completeness given in Definition 4.3, by specifying the set for which the set of picking strategies is $\textrm{EU}$-complete:

#### Definition C.4

${\mathcal {N}}$ is $\textrm{EU}$-complete for ${\mathbb {B}}$ iff for every $b\in {\mathbb {B}}$, there is some ${\mathsf {n}}^b\in {\mathcal {N}}$ such that ${\mathsf {n}}^b$ picks for $\textrm{EU}_{b(\cdot |-)}$.

Note that, if ${\mathcal {N}}$ is deterministically full, i.e., ${\mathcal {N}}\supseteq {\mathcal {S}}$, then it is $\textrm{EU}$-complete for any ${\mathbb {B}}$.

#### Corollary C.5

Suppose ${\mathcal {N}}$ is $\textrm{EU}$-complete for ${\mathbb {B}}$.

(i) ${\mathsf {n}}\in \textrm{EAd}_{\mathbb {B}}({\mathcal {N}})$ iff there is some $b\in {\mathbb {B}}$ such that ${\mathsf {n}}$ $b_{\mathcal {D}}$-picks for $\textrm{EU}_{b(\cdot |-)}$.
(ii) If $p\times \mu \in {\mathbb {B}}$ and ${\mathsf {n}}$ *μ *-surely picks for $\textrm{EU}_p$, then ${\mathsf {n}}\in \textrm{EAd}_{\mathbb {B}}({\mathcal {N}})$.
(iii) If ${\mathbb {B}}$ makes *Ω * and ${\mathcal {D}}$ completely independent, then ${\mathsf {n}}\in \textrm{EAd}_{\mathbb {B}}({\mathcal {N}})$ iff there is some $p\times \mu \in {\mathbb {B}}$ such that ${\mathsf {n}}$ *μ *-surely picks for $\textrm{EU}_p$.

#### Proof

Immediate from Lemma C.3 and Theorem C.1. *□ *

This gives Propositions 3.5, 3.9 and 4.6. For Propositions 3.4 and 3.10, we will use a further lemma.

#### Lemma C.6

(i) If $p\in {\mathbb {P}}$ and ${\mathsf {n}}$ picks for $\textrm{EU}_p$, then ${\mathsf {n}}$ picks for $\textrm{EAd}_{\mathbb {P}}$.
(ii) If $b\in {\mathbb {B}}$ and ${\mathsf {n}}$ picks for $\textrm{EU}_{b(\cdot |-)}$, then ${\mathsf {n}}$ picks for $\textrm{EAd}_{{\mathbb {B}}(\cdot |-)}$.

#### Proof

It follows from the definition of $\textrm{EAd}_{\mathbb {P}}$ that, for all *D* and $p \in {\mathbb {P}}$, $\textrm{EU}_p(D)\subseteq \textrm{EAd}_{\mathbb {P}}(D)$.

If ${\mathsf {n}}$ picks for $\textrm{EU}_p$, then for all $D\in {\mathcal {D}}$, ${\mathsf {n}}_D(\textrm{EU}_p(D))=1$. And so, since $\textrm{EU}_p(D)\subseteq \textrm{EAd}_{\mathbb {P}}(D)$, ${\mathsf {n}}_D(\textrm{EAd}_{\mathbb {P}}(D))=1$, i.e. ${\mathsf {n}}$ picks for $\textrm{EAd}_{\mathbb {P}}$.

An analogous argument gives (ii), as, for all *D*, $\textrm{EU}_{b(\cdot |-)}(D)\subseteq \textrm{EAd}_{{\mathbb {B}}(\cdot |-)}(D)$. *□ *

#### Corollary C.7

(i) Suppose $p\in {\mathbb {P}}$, $p\times \mu \in {\mathbb {B}}$ and ${\mathsf {n}}$ picks for $\textrm{EU}_p$. Then ${\mathsf {n}}$ picks for $\textrm{EAd}_{\mathbb {P}}$ and is in $\textrm{EAd}_{\mathbb {B}}({\mathcal {N}})$.
(ii) Suppose $b\in {\mathbb {B}}$ and ${\mathsf {n}}$ picks for $\textrm{EU}_{b(\cdot |-)}$. Then ${\mathsf {n}}$ picks for $\textrm{EAd}_{{\mathbb {B}}(\cdot |-)}$ and is in $\textrm{EAd}_{\mathbb {B}}({\mathcal {N}})$.

#### Proof

(i): From Lemma C.6, ${\mathsf {n}}$ picks for $\textrm{EAd}_{\mathbb {P}}$. By Corollary C.5, ${\mathsf {n}}\in \textrm{EAd}_{\mathbb {B}}({\mathcal {N}})$.

(ii): From Lemma C.6, ${\mathsf {n}}$ picks for $\textrm{EAd}_{{\mathbb {B}}(\cdot |-)}$. By Corollary C.5, ${\mathsf {n}}\in \textrm{EAd}_{\mathbb {B}}({\mathcal {N}})$. *□ *

Propositions 3.4 and 3.10 follow from this.

We have thus proved Propositions 3.4, 3.5, 3.9, 3.10 and 4.6.

### C.3 Maximality (Propositions 3.7, 3.15 and 4.8)

As Maximality is a more permissive theory than E-Admissibility, Corollaries C.7 and C.5 also imply our results that the relevant expected utility strategies both pick for Maximality and are themselves judged as Maximal (Propositions 3.7, 3.15 and 4.8); so that Maximality is not undermining in the way that we’ve seen the risk-sensitive decision theories or *Γ *-maximin are.

### C.4 Underminingness of E-Admissibility (Propositions 3.6, 3.11 and 4.7)

We now move to Propositions 3.6, 3.11 and 4.7. For these results, we need to give conditions under which there exists a strategy that picks for E-Admissibility and does not look like an expected utility strategy from the point of view of our measure *μ * over the decision problems.

For regular picking strategies, this is simple: E-Admissibility must disagree with each $\textrm{EU}_p$, at least *μ *-surely.

#### Proposition C.8

(Proposition 4.7) Suppose that, for every $b\in {\mathbb {B}}$, $b_{\mathcal {D}}\{D\vert \textrm{EAd}_{{\mathbb {B}}(\cdot |-)}(D)\subseteq \textrm{EU}_{b(\cdot |-)}(D)\}<1$.

Then, if ${\mathsf {n}}$ is a regular picking strategy for $\textrm{EAd}_{{\mathbb {B}}(\cdot |-)}$, then ${\mathsf {n}}\notin \textrm{EAd}_{\mathbb {B}}({\mathcal {N}})$.

#### Proof

Suppose ${\mathsf {n}}$ is a regular picking strategy for $\textrm{EAd}_{{\mathbb {B}}(\cdot |-)}$ and the conditions of the theorem hold.

Take any $b\in {\mathbb {B}}$. If $\textrm{EAd}_{{\mathbb {B}}(\cdot |-)}(D)\not \subseteq \textrm{EU}_{b(\cdot |-)}(D)$, then, since ${\mathsf {n}}_D$ is a regular probability function, ${\mathsf {n}}_D(\textrm{EU}_{b(\cdot |-)}(D))< 1$. Since $b_{\mathcal {D}}\{D\vert \textrm{EAd}_{{\mathbb {B}}(\cdot |-)}(D)\not \subseteq \textrm{EU}_{b(\cdot |-)(D)}\}>0$, we have $\mu \{D\vert {\mathsf {n}}(\textrm{EU}_{b(\cdot |-)}(D))<1\}>0$. So ${\mathsf {n}}$ does not $b_{\mathcal {D}}$-surely pick for $\textrm{EU}_{b(\cdot |-)}$.

By Corollary C.5, it follows that ${\mathsf {n}}\notin \textrm{EAd}_{\mathbb {B}}({\mathcal {N}})$. *□ *

To ensure the existence of *deterministic* picking strategies that are not $\textrm{EU}_p$ strategies, this assumption does not suffice, as the following example demonstrates.

#### Example C.9

If ${\mathbb {P}}=\{p_1,p_2\}$ and $p_1$ expects $a_1$ to be better than $a_2$ and $p_2$ expects $a_2$ to be better than $a_1$. Suppose *μ * is sure you’ll face $D^*=\{a_1,a_2\}$, i.e., $\mu (\{D^*\}) = 1$. Then picking strategies are determined by their selection on this single decision problem, picking either $a_1$ or $a_2$. So any strategy is *μ *-surely an $\textrm{EU}_p$ strategy for some $p \in {\mathbb {P}}$, even though, for each $p \in {\mathbb {P}}$, $\mu \{D\vert \textrm{EAd}_{\mathbb {P}}(D)\subseteq \textrm{EU}_p(D)\}< 1$.

Instead, the requirement of coordination needs to be visible from the point of view of the measure *μ *. There need to be distinct decisions where the probabilities in ${\mathbb {P}}$ require coordination across the decisions, but E-Admissibility does not. This is what happens in the Ellsberg case or the Coordination cases discussed in the main text.

In these cases, we have distinct decisions and probabilities such that every probability function disagrees with the recommendations of $\textrm{EU}_{q^*_i}$ on $D_i$, and the $D_i$ have positive measure according to every *μ *. More generally:

#### Proposition C.10

(Proposition 3.6) Suppose ${\mathbb {B}}$ makes *Ω * and ${\mathcal {D}}$ completely independent (so every $b\in {\mathbb {B}}$ has the form $p\times \mu$.)

Suppose there is a selection of measurable, pairwise disjoint events $E_q\subseteq {\mathcal {D}}$, one for each $q\in {\mathbb {P}}$ (some of which may be empty), such that for all $p\times \mu \in {\mathbb {B}}$,

$$\begin{aligned} \mu \left( \bigcup _{q\in {\mathbb {P}}}\{D\in E_q\vert \textrm{EU}_p(D)\cap \textrm{EU}_q(D)=\varnothing \}\right) >0 \end{aligned}$$

Then there is ${\mathsf {s}}$ that picks for $\textrm{EAd}_{\mathbb {P}}$ but which is not in $\textrm{EAd}_{\mathbb {B}}({\mathcal {S}})$.

#### Proof

Define ${\mathsf {s}}$ as follows:

- for $D \in E_q$, ${\mathsf {s}}(D)$ is any member of $\textrm{EU}_q(D)$;
- for $D\notin \bigcup _{q\in {\mathbb {P}}}E_q$, ${\mathsf {s}}(D)$ is any member of $\textrm{EAd}_{\mathbb {B}}(D)$.

As they are disjoint, this is well-defined.

Moreover, such an ${\mathsf {s}}$ can be chosen to be measurable. For each *q* there is a measurable ${\mathsf {s}}_q$ (Lemma B.1) and so we can specify ${\mathsf {s}}(D)={\mathsf {s}}_q(D)$ for $D\in E_q$ and ${\mathsf {s}}(D)={\mathsf {s}}_{q_0}(D)$ for $D\notin \bigcup _{q\in {\mathbb {P}}}E_q$ where $q_0\in {\mathbb {P}}$. As $E_q$ are assumed to be measurable, this will be a measurable function.

Then consider any $p \times \mu \in {\mathbb {B}}$. If ${\mathsf {s}}(D)\in \textrm{EU}_q(D)$ and $\textrm{EU}_p(D)\cap \textrm{EU}_q(D)=\varnothing$, then ${\mathsf {s}}(D)\notin \textrm{EU}_p(D)$. So, for each $q\in {\mathbb {P}}$, $\{D\in E_q\vert \textrm{EU}_p(D)\cap \textrm{EU}_q(D)=\varnothing \}\subseteq \{D\vert {\mathsf {s}}(D)\notin \textrm{EU}_p(D)\}$. Thus,

$$\begin{aligned} \{D\vert {\mathsf {s}}(D)\notin \textrm{EU}_p(D)\}\supseteq \bigcup _{q\in {\mathbb {P}}}\{D\in E_q\vert \textrm{EU}_p(D)\cap \textrm{EU}_q(D)=\varnothing \}. \end{aligned}$$

By our assumption on *μ *, it follows that $\mu (\{D\vert {\mathsf {s}}(D)\notin \textrm{EU}_p(D)\})>0$. So ${\mathsf {s}}$ does not *μ *-surely pick for $\textrm{EU}_p$. *□ *

#### Proposition C.11

(Proposition 3.11) Suppose there is a selection of measurable, pairwise disjoint events $E_{b'}\subseteq {\mathcal {D}}$, one for each $b'\in {\mathbb {B}}$ (some of which may be empty), such that for all $b\in {\mathbb {B}}$,

$$\begin{aligned} b_{\mathcal {D}}\left( \bigcup _{b'\in {\mathbb {B}}}\{D\in E_{b'}\vert \textrm{EU}_{b(\cdot |-)}(D)\cap \textrm{EU}_{b'(\cdot |-)}(D)=\varnothing \}\right) >0. \end{aligned}$$

Then there is ${\mathsf {s}}$ which picks for $\textrm{EAd}_{{\mathbb {B}}(\cdot |-)}$ but where there is no $b\in {\mathbb {B}}$ such that ${\mathsf {s}}$ $b_{\mathcal {D}}$-surely picks for $\textrm{EU}_{b(\cdot |-)}$.

#### Proof

As above, except ensure ${\mathsf {s}}(D)$ some member of $\textrm{EU}_{b(\cdot |D)}(D)$, for $D\in E_b$; and ${\mathsf {s}}(D)$ some member of $\textrm{EAd}_{{\mathbb {B}}(\cdot |-)}(D)$, for $D \notin \bigcup _{b\in {\mathbb {B}}}E_b$. *□ *

We can find cases with such events when we make additional assumptions that each *μ * is sufficiently spread.

#### Proposition C.12

Suppose that ${\mathbb {B}}$ makes *Ω * and ${\mathcal {D}}$ completely independent.

Assume ${\mathfrak {U}}({\mathcal {A}})=[l,h]^\Omega$ with *l<0<h*. In particular, for every $X\in [l,h]^\Omega$ there is some $a\in {\mathcal {A}}$ with ${\mathfrak {U}}(a)=X$.

Suppose ${\mathbb {P}}$ is non-singleton and for every $p \times \mu \in {\mathbb {B}}$, for every open non-empty subset $U \subseteq {\mathcal {A}}$, $\mu \{\{a,0\}\vert a\in U\}>0$.

Then there is ${\mathsf {s}}$ that picks for $\textrm{EAd}_{\mathbb {P}}$ but for which there is no $p\times \mu \in {\mathbb {B}}$ such that ${\mathsf {s}}$ *μ *-surely pick for $\textrm{EU}_p$.

***Proof.***

#### Sublemma C.12.1

Suppose *V* is a non-empty open subset of $[l,h]^\Omega$ such that for each $X\in {\mathbb {R}}^\Omega$ there is some *λ >0* such that $\lambda X\in V$.

Then, for any *p≠ q*, $\{X\in V\vert \mathsf {Exp}_p[X]>0>\mathsf {Exp}_q[X]\}$ is non-empty and open.

#### Proof

It is non-empty: First, observe that there is some $X\in {\mathbb {R}}^\Omega$ such that $\mathsf{Exp}_p[X]>0>\mathsf{Exp}_q[X]$. For example, with *A* such that *p(A)≠ q(A)*, we can put $X={\textbf{1}}_A-\frac{p(A)-q(A)}{2}{\textbf{1}}$; or the negative of this, if required. By our assumption concerning *V*, there is some *λ >0* with $\lambda X\in V$. And so $\mathsf{Exp}_p[\lambda X]>0>\mathsf{Exp}_{q}[\lambda X]$.

It is open by continuity of expectation. *□ *

Thus, for any $p\times \mu \in {\mathbb {B}}$ and any *q≠ p*,

$$\begin{aligned} \mu (\{\{a,0\}\vert {\mathfrak {U}}(a)\in V\text { and }\mathsf{Exp}_p[{\mathfrak {U}}(a)]>0>\mathsf{Exp}_q[{\mathfrak {U}}(a)] \})>0. \end{aligned}$$

Note also that for $D=\{a,0\}$ with $\mathsf{Exp}_p[{\mathfrak {U}}(a)]>0>\mathsf{Exp}_q[{\mathfrak {U}}(a)]$, then $\textrm{EU}_{p}(D)=\{a\}$ and $\textrm{EU}_q(D)=\{0\}$, so they are disjoint. For $E_V:=\{\{a,0\}\vert a\in V\}$, then $\mu (\{D\in E_V\vert \textrm{EU}_p(D)\cap \textrm{EU}_q(D)=\varnothing \})>0$.

To construct our disjoint $E_q$ satisfying the conditions of Proposition 3.6, first note that we can find two *disjoint* $V_1$ and $V_2$ that are non-empty open subsets of $[l,h]^\Omega$ such that for each $i\in \{1,2\}$ and each $X\in {\mathbb {R}}^\Omega$ there is some *λ >0* such that $\lambda X\in V_i$. For example, we could put $V_1:=\{X\in [l,h]^n\vert \Vert X\Vert <0.5\}$ and $V_2:=\{X\in [l,h]^n\vert \Vert X\Vert >0.5\}$ (assuming that *l<-0.5<0.5<h*).

Then take any distinct $q^*_1,q^*_2\in {\mathbb {P}}$.

Put

$$\begin{aligned} E_q:={\left\{ \begin{array}{ll} E_{V_1}=\{\{a,0\}\vert a\in V_1\}& q=q^*_1\\ E_{V_2}=\{\{a,0\}\vert a\in V_2\}& q=q^*_2\\ \varnothing & \text {otherwise} \end{array}\right. } \end{aligned}$$

and observe that the conditions of Proposition 3.6 are then satisfied. *□ *

#### Proposition C.13

Suppose that ${\mathbb {B}}$ makes *Ω * and ${\mathcal {D}}$ completely independent.

Assume ${\mathfrak {U}}({\mathcal {A}})=[l,h]^\Omega$. In particular, for every $X\in [l,h]^\Omega$ there is some $a\in {\mathcal {A}}$ with ${\mathfrak {U}}(a)=X$.

Suppose ${\mathbb {P}}$ is non-singleton and for every $p \times \mu \in {\mathbb {B}}$, *μ * has full support on ${\mathcal {D}}$ (assigning strictly positive measure to every non-empty open subset of ${\mathcal {D}}$).

Then there is ${\mathsf {s}}$ that picks for $\textrm{EAd}_{\mathbb {P}}$ but for which there is no $p\times \mu \in {\mathbb {B}}$ such that ${\mathsf {s}}$ *μ *-surely pick for $\textrm{EU}_p$.

***Proof.***

#### Sublemma C.13.1

Let $E = \{D\in {\mathcal {D}}\vert D\subseteq V\}$ for some open subset $V \subseteq [l,h]^\Omega$.

For any *p≠ q*, $\{D\in E\vert \textrm{EU}_{p}(D)\cap \textrm{EU}_{q}(D)=\varnothing \}$ is open and non-empty $\subseteq {\mathcal {D}}$.

#### Proof

**It is nonempty:**

Take any $D_0\in E$. If $\textrm{EU}_{p}(D_0)\cap \textrm{EU}_{q}(D_0)=\varnothing$ this suffices.

Otherwise, there is some $a_0\in \textrm{EU}_{p}(D_0)\cap \textrm{EU}_{q}(D_0)$.

As in Proposition 3.6, we can find some $X^*$ such that $\mathsf{Exp}_{p}[X^*]>0>\mathsf{Exp}_{q}[X^*]$. And, moreover, we can choose a scalar *λ * small enough so that ${\mathfrak {U}}(a_0)+\lambda X^*\in [l,h]^n$ and thus that $a_0+\lambda X^*\in {\mathfrak {U}}({\mathcal {A}})$.

So $$\mathsf{Exp}_{p}[{\mathfrak {U}}(a_0+\lambda X^*)]>\mathsf{Exp}_{p}[{\mathfrak {U}}(a_0)]$$ and $$\mathsf{Exp}_{q}[{\mathfrak {U}}(a_0+\lambda X^*)]<\mathsf{Exp}_{q}[{\mathfrak {U}}(a_0)]$$.

Consider $D^*=D_0\cup \{a_0+\lambda X^*\}$, recalling that $a_0\in D_0$.

Observe that $\textrm{EU}_{p}(D^*)=\{a_0+\lambda X^*\}$ but that $a_0+\lambda X^*\notin \textrm{EU}_{q}(D^*)$, since *q* finds $a_0$ preferable. Thus $\textrm{EU}_{p}(D^*)\cap \textrm{EU}_{q}(D^*)=\varnothing$.

Since ${\mathfrak {U}}(a_0)+\lambda X^*\in V$, by choice of *λ *, $D^*\subseteq V$, and so $D^*\in E$, as required.

**It is open:**

Take any $D_0\in E$ with $\textrm{EU}_{p}(D_0)\cap \textrm{EU}_{q}(D_0)=\varnothing$.

Let $$t_p=\max _{a\in D_0}\mathsf{Exp}_{p}[{\mathfrak {U}}(a)]$$ and $$t_q=\max _{a\in D_0}\mathsf{Exp}_{q}[{\mathfrak {U}}(a)]$$.

As $\textrm{EU}_{p}(D_0)\cap \textrm{EU}_{p}(D_0)=\varnothing$, for all $a\in D_0$, either $t_p>\mathsf{Exp}_{p}[\mathfrak{U}(a)]$ or $t_q>\mathsf{Exp}_{q}[\mathfrak{U}(a)]$. So, define $f(a):=\max \{t_p-\mathsf{Exp}_{p}[{\mathfrak {U}}(a)],t_q-\mathsf{Exp}_{q}[{\mathfrak {U}}(a)]\}>0$, for all $a\in D_0$. Since $D_0$ is finite, let $\delta =\min _{a\in D_0}f(a)>0$. So that for all $a\in D_0$, either $\mathsf{Exp}_{p}[{\mathfrak {U}}(a)]\leqslant t_p-\delta$ or $\mathsf{Exp}_{q}[{\mathfrak {U}}(a)]\leqslant t_q-\delta$  

Let $\epsilon =\delta/2$. Suppose $d(D_0,D)<\epsilon$. That is, for all $a\in D_0$, there is some *c∈ D* such that *d(a,c)<ε * and, for all *c∈ D*, there is some $a\in D_0$ such that *d(a,c)<ε *.

Take any $a_p\in \textrm{EU}_{p}(D_0)$, so $\mathsf{Exp}_{p}[{\mathfrak {U}}(a_p)]=t_p$. There is some $c_p\in D$ such that $d(a_p,c_p)<\epsilon$, and thus $\mathsf{Exp}_{p}[{\mathfrak {U}}(c_p)]>t_p-\epsilon$. Thus, $\max _{c\in D}\mathsf{Exp}_{p}[{\mathfrak {U}}(c)]>t_p-\epsilon$, and so if $c\in \textrm{EU}_{p}(D)$, then $\mathsf{Exp}_{p}[{\mathfrak {U}}(c)]>t_p-\epsilon$.

By an analogous argument, if $c\in \textrm{EU}_{p}(D)$ then $\mathsf{Exp}_{q}[{\mathfrak {U}}(c)]>t_q-\epsilon$.

For any *c∈ D*, there is some $a_c\in D_0$ such that $d(c,a_c)<\epsilon$. If, also, $c\in \textrm{EU}_{p}(D)\cap \textrm{EU}_{p}(D)$ then

$$\begin{aligned}&\mathsf{Exp}_{p}[{\mathfrak {U}}(a_c)]>\mathsf{Exp}_{p}[{\mathfrak {U}}(c)]-\epsilon>t_p-2\epsilon \\ \text {and }&\mathsf{Exp}_{q}[{\mathfrak {U}}(a_c)]>\mathsf{Exp}_{q}[{\mathfrak {U}}(c)]-\epsilon >t_q-2\epsilon \end{aligned}$$

By choice of $\epsilon =\delta/2$ there is no such $a_c\in D_0$.

*□ *

Let $V_1,V_2$ be any disjoint non-empty open subsets of $[l,h]^\Omega$.

Then take any distinct $q^*_1,q^*_2\in {\mathbb {P}}$.

Let

$$\begin{aligned} E_q:={\left\{ \begin{array}{ll} \{D\in {\mathcal {D}}\vert D\subseteq V_1\}& q=q^*_1\\ \{D\in {\mathcal {D}}\vert D\subseteq V_2\}& q=q^*_2\\ \varnothing & \text {otherwise} \end{array}\right. } \end{aligned}$$

and observe that the conditions of Proposition 3.6 are then satisfied. *□ *

To prove Proposition 3.12, let ${\mathsf {c}}$ be any choice function. Let $r:{\mathcal {D}}\rightarrow [0,1)$ be any measurable statistic, e.g., $r(D)=\Bigl (\sum _{x\in D}\Vert x\Vert ^{2}\Bigr )\bmod 1$.

#### Proposition C.14

(Proposition 3.12) Suppose that for any $b\in {\mathbb {B}}$, $b_{\mathcal {D}}(U_b)>0$ where

$$\begin{aligned} U_b:=\bigl \{D\in {\mathcal {D}}\vert {\mathsf {c}}(D)\not \subseteq \textrm{EU}_{b(\cdot |-)}(D)\bigr \}. \end{aligned}$$

Suppose further that for any $b\in {\mathbb {B}}$ and any open interval *I⊂ [0,1)*,

$$\begin{aligned} b_{\mathcal {D}}\bigl (\,D\in U_b\vert r({\mathsf {c}}(D))\in I \bigr )>0. \end{aligned}$$

Then there exists a measurable function ${\mathsf {s}}:{\mathcal {D}}\rightarrow \mathbb {\mathcal {A}}$ which picks for ${\mathsf {c}}$ but for no $b\in {\mathbb {B}}$ does it $b_{\mathcal {D}}$-surely pick for $\textrm{EU}_{b(\cdot |-)}$.

#### Proof

For any $D\in {\mathcal {D}}$, enumerate the choice set in lexicographic order ${\mathsf {c}}(D)=\{x_{1}(D),\dots ,x_{n_D}(D)\},$ where $n_D=|{\mathsf {c}}(D)|$. Partition [0, 1) into $n_D$ intervals $I_{j}(D)=\bigl [\tfrac{j-1}{n_D},\tfrac{j}{n_D}\bigr )$. Define

$$\begin{aligned} \alpha (D):=\min \bigl \{j\vert r({\mathsf {c}}(D))\in I_{j}(D)\bigr \}, \qquad s(D):=x_{\alpha (D)}(D). \end{aligned}$$

Measurability follows immediately from the measurability of *r* and *\min *, and $s(D)\in {\mathsf {c}}(D)$ by construction. So ${\mathsf {s}}$ picks for ${\mathsf {c}}$.

Choose $$b\in {\mathbb {B}}$$.

For each $D\in U_b$, choose $x_{\beta (D)}(D)\in {\mathsf {c}}(D){\setminus }\textrm{EU}_{b(\cdot |-)}(D)$. If $r({\mathsf {c}}(D))\in I_{\beta (D)}(D)$ then $s(D)=x_{\beta (D)}(D)\notin \textrm{EU}_{b(\cdot |-)}(D)$.

Define $V_b:=\{D\in U_b\vert r({\mathsf {c}}(D))\in I_{\beta (D)}(D)\}.$ Because $I_{\beta (D)}(D)$ has positive length $1/n_D$ (and hence contains an open interval), we must have $b_{\mathcal {D}}(V_b)>0$.

For all $D\in V_b$, $s(D)\notin \textrm{EU}_{b(\cdot |-)}(D)$, hence

$$\begin{aligned} b_{\mathcal {D}}\bigl (\,D\vert s(D)\notin \textrm{EU}_{b(\cdot |-)}(D)\bigr )\ge b_{\mathcal {D}}(V_b)>0. \end{aligned}$$

Therefore $b_{\mathcal {D}}\bigl (\,D\vert s(D)\in \textrm{EU}_{b(\cdot |-)}(D)\bigr )<1.$ So ${\mathsf {s}}$ does not $b_{\mathcal {D}}$-surely pick for $\textrm{EU}_{b(\cdot |-)}$.

*□ *

So we have proved Propositions  2.3, 2.5, 2.7, 3.4 to 3.7, 3.9, 3.10, 3.12, 3.15 and 4.5 to 4.8

Proposition 4.10, 4.12, 4.15, 4.17 and 4.18 require a different approach.

## D Maximality (Propositions 4.10, 4.12, 4.15, 4.17 and 4.18)

To prove Propositions 4.10, 4.12, 4.15, 4.17 and 4.18 we turn to a version of Abraham Wald’s (1947) Complete Class Theorem, which we prove as Theorem D.7. We state it in a general setting and then explain how they apply to our case. Along the way, we prove a more standard version of Wald’s theorem.

### D.1 Wald theorem

To state them, we need some definitions.

- *Ω * is a finite set of states.
- A probability function over *Ω * is a normalised function $p: \Omega \rightarrow [0, 1]$, i.e., $\sum _{\omega \in \Omega } p(w) = 1$. Given a random variable (or vector) $X\in {\mathbb {R}}^\Omega$, we write *p*(*X*) for the expectation of *X*, i.e., $p(X)=\mathsf{Exp}_p[X] = \sum _{\omega \in \Omega }p(\omega )X(\omega )$.
- ${\mathcal {O}}$ is a set of “options”. In our application it will be ${\mathcal {N}}$, containing (probabilistic) picking strategies, ${\mathsf {n}}$.
- ${\mathcal {U}}:{\mathcal {O}}\times \Omega \rightarrow [l,u]$ is a bounded “utility function”. In our application, it will be ${\mathcal {U}}({\mathsf {n}},\omega )=\mathsf{Exp}_{\mu ^*}[{\mathfrak {U}}({\mathsf {n}},\omega )]$.^22^
- Given an option *o*, the utility profile of *o* is the random variable (or vector) ${\mathcal {U}}(o) \in {\mathbb {R}}^\Omega$ with ${\mathcal {U}}(o)(\omega )={\mathcal {U}}(o,\omega )$.
- So, given an option *o* and a probability function *p* over *Ω *, $p({\mathcal {U}}(o)) = \mathsf{Exp}_p[{\mathcal {U}}(o)] = \sum _{\omega \in \Omega } p(\omega ) {\mathcal {U}}(o)(\omega ) = \sum _{\omega \in \Omega } p(\omega ) {\mathcal {U}}(o, \omega )$.

#### Definition D.1

Relative to $${\mathcal {U}}$$:

*o* is *strictly dominated in* ${\mathcal {O}}$ iff there is $o'\in {\mathcal {O}}$ with ${\mathcal {U}}(o',\omega )>{\mathcal {U}}(o,\omega )$ for all *ω *. *o* is called *admissible* if it is not strictly dominated.

*o* is *weakly dominated in* ${\mathcal {O}}$ iff there is $o'\in {\mathcal {O}}$ with ${\mathcal {U}}(o',\omega )\geqslant {\mathcal {U}}(o,\omega )$ for all *ω * and ${\mathcal {U}}(o',\omega )>{\mathcal {U}}(o,\omega )$ for some *ω *.

*o* is *Bayes for* *p* *in* ${\mathcal {O}}$ iff, for all $o'\in {\mathcal {O}}$, $p({\mathcal {U}}(o))\geqslant p({\mathcal {U}}(o'))$.

*o* is *Bayes in* ${\mathcal {O}}$ iff there is some probability *p* such that *o* is Bayes for *p*.

#### Lemma D.2

If *o* is Bayes in ${\mathcal {O}}$, then it is not strictly dominated in ${\mathcal {O}}$.

#### Proof

Suppose it is strictly dominated. Then there is *o'* such that ${\mathcal {U}}(o,\omega )<{\mathcal {U}}(o',\omega )$, for all *ω *. So every probability function *p* over *Ω *, we have $p( {\mathcal {U}}(o))<p({\mathcal {U}}(o'))$. Thus it is not Bayes.

*□ *

From now on, we’ll think directly in terms of vectors. We let ${\mathcal {U}}({\mathcal {O}}) := \lbrace {{\mathcal {U}}(o)\vert o\in {\mathcal {O}}} \rbrace \subseteq {\mathbb {R}}^\Omega$, and we write $\mathsf{ConvHull}({\mathcal {U}}({\mathcal {O}}))$ for the convex hull of this set of real-valued vectors. We write $\textrm{cl}(\mathsf{ConvHull}({\mathcal {U}}({\mathcal {O}})))$ for the closure of $\mathsf{ConvHull}({\mathcal {U}}({\mathcal {O}}))$ in the product topology. This can also be characterised by limits of sequences, or more generally of nets: if a sequence (or net) of members of $\mathsf{ConvHull}({\mathcal {U}}({\mathcal {O}}))$ is such that, for each coordinate, *ω *, $X_\alpha (\omega )\longrightarrow X^*(\omega )$, then $X^*\in \textrm{cl}(\mathsf{ConvHull}({\mathcal {U}}({\mathcal {O}})))$.

The definitions of strict and weak dominance, and the definitions of being Bayes for *p* and being Bayes simpliciter carry over straightforwardly to vectors. For instance, given two vectors $X, Y \in {\mathbb {R}}^\Omega$, we say *X* strictly dominates *Y* if $X(\omega ) > Y(\omega )$, for all *ω *; and we say that *X* is Bayes for *p* in a given set of vectors if *p(X) ⩾ p(Y)*, for all *Y* in that set; and so on.

Our first lemma says that if a vector is not Bayes in ${\mathcal {U}}({\mathcal {O}})$, then it is strictly dominated in the convex hull of ${\mathcal {U}}({\mathcal {O}})$.

#### Lemma D.3

If $X\in {\mathbb {R}}^\Omega$ and there is no probability *p* with $p(X)\geqslant p({\mathcal {U}}(o))$, for all $o\in {\mathcal {O}}$, then there is $Y\in \mathsf{ConvHull}({\mathcal {U}}(O))$ such that $Y(\omega )>X(\omega )$, for all *ω *.

#### Proof

Suppose *X* is not strictly dominated in $\mathsf{ConvHull}({\mathcal {U}}({\mathcal {O}}))$. Let $\mathsf{Dom}_X$ be the set of strict dominators of *X*, i.e.,

$$\begin{aligned} \mathsf{Dom}_X:=\{Y\in {\mathbb {R}}^\Omega \vert \text {for all } \omega , Y(\omega )>X(\omega )\}. \end{aligned}$$

Then, by assumption, $\mathsf{Dom}_X$ and $\mathsf {ConvHull}({\mathcal {U}}({\mathcal {O}}))$ are disjoint. They are also both convex. And so, by the Separating Hyperplane Theorem, they can be separated by a non-zero linear functional (Boyd & Vandenberghe, 2004, Section 2.5.1). That is, there is a linear functional, $f:{\mathbb {R}}^\Omega \rightarrow {\mathbb {R}}$, with *f≠ 0* and a constant *c* such that $f(Y)\geqslant c \geqslant f(Z)$ for any $Y\in \mathsf {Dom}_X$ and $Z\in \mathsf{ConvHull}({\mathcal {U}}({\mathcal {O}}))$.

We now show that *f* is non-negative. Let *Z⩾ 0* and suppose *f(Z)<0*. Then take any $Y\in \mathsf{Dom}_X$ and any *k > 0*, and note that $Y+kZ \in \mathsf{Dom}_X$, and so *f(Y + kZ) ⩾ c*. Then, since *f(Z) < 0*, we can make *f(Y+kZ)=f(Y)+kf(Z)* arbitrarily small by making *k* arbitrarily large. And, in particular, we can make *f(Y+kZ)=f(Y)+kf(Z)* less than *c*. This gives a contradiction.

Also, since *f ≠ 0*, there is *ω * such that $f(\omega )\ne 0$. After all, by linearity, $f(X) = \sum _{\omega \in \Omega }f(\omega )X(\omega )$, and so, if *f(ω ) = 0*, for all *ω *, *f(X) = 0*, for all $X \in {\mathbb {R}}^\Omega$, i.e., *f = 0*, which gives a contradiction.

We can thus normalise *f* to obtain our probability function *p* over *Ω * with $p(Y)\geqslant c' \geqslant p(Z)$, for any $Y\in \mathsf{Dom}_X$ and $Z\in \mathsf{ConvHull}({\mathcal {U}}({\mathcal {O}}))$.

Now, $X\in \textrm{cl}(\mathsf{Dom}_X )$, and so *p(X)⩾ c'*, and so *p(X) ⩾ p(Z)*, for all $Z\in {\mathcal {U}}({\mathcal {O}})\subseteq \mathsf{ConvHull}({\mathcal {U}}({\mathcal {O}}))$, as required. *□ *

This now gives us a standard version of Wald’s Complete Class Theorem, which says: if ${\mathcal {U}}({\mathcal {O}})$ is convex, then *o* is Bayes in ${\mathcal {O}}$ iff *o* is not strictly dominated in ${\mathcal {O}}$.^23^ But this is not the version we apply to obtain our results. So we continue.

This result doesn’t show that, if *o* is not Bayes, it is strictly dominated by something that is itself not dominated, and thus is Bayes. We get that from the next result.

#### Lemma D.4

Suppose *X* is strictly dominated in $\mathsf{ConvHull}({\mathcal {U}}({\mathcal {O}}))$. Then we can find $Z\in \textrm{cl}(\mathsf{ConvHull}({\mathcal {U}}({\mathcal {O}})))$ that strictly dominates *X* and is itself not even weakly dominated in $\mathsf{ConvHull}({\mathcal {U}}({\mathcal {O}}))$.

In the usual setting for Wald’s Complete Class Theorem, the vector *Z* in $\textrm{cl}(\mathsf{ConvHull}({\mathcal {U}}({\mathcal {O}})))$ that dominates *X* and is not weakly dominated is called the ‘lower boundary’ of the set; in our case, it would be ‘upper boundary’, since we are working with positive utility rather than risk or disutility. This then says that, if a vector is dominated, it is dominated by something in the lower (upper) boundary. The result depends essentially on the fact that ${\mathcal {U}}({\mathcal {O}})$ is bounded from above.

#### Proof

Take any $Y\in \mathsf{ConvHull}({\mathcal {U}}({\mathcal {O}}))$ that dominates *X*. Consider $A:=\{Z\in \textrm{cl}(\mathsf{ConvHull}({\mathcal {U}}({\mathcal {O}})))\vert Z(\omega )\geqslant Y(\omega )\text { for all }\omega \}$. Observe that this is closed (as it is the intersection of two closed sets) and bounded (as we assumed that utilities were bounded above), and thus compact. Let $f(Z):=\sum _\omega Z(\omega )$. Since *f* is a continuous function, the Extreme Value Theorem ensure it obtains its maximum somewhere in *A*. This maximum point will be as required. *□ *

This result can also be proved by applying Zorn’s lemma. That argument also works when *Ω * is infinite.^24^

This will not apply to our general case yet. For that, we need further assumptions on the relationship between ${\mathcal {U}}$ and ${\mathcal {O}}$.

#### Definition D.5

- ${\mathcal {O}}$ is *Bayes-existing* (relative to ${\mathcal {U}}$) iff, for every probability *p*, there is some $o\in {\mathcal {O}}$ that is Bayes for *p*.
- ${\mathcal {U}}$ is *Bayes-continuous* on ${\mathcal {O}}$ iff, for all $o\in {\mathcal {O}}$, if *o* is Bayes for *p* and $p_1,p_2,\ldots$ are probabilities converging to *p* then there is some sequence of options $o_1,o_2,\ldots$, with $o_n$ Bayes for $p_n$, whose utility profiles converge to those of *o*.

#### Lemma D.6

Suppose ${\mathcal {U}}$ is Bayes-continuous on ${\mathcal {O}}$ and ${\mathcal {O}}$ is Bayes-existing. Then, if $Z\in \textrm{cl}(\mathsf{ConvHull}({\mathcal {U}}({\mathcal {O}})))$ is not weakly dominated in $\mathsf{ConvHull}({\mathcal {U}}({\mathcal {O}}))$, then $Z\in {\mathcal {U}}({\mathcal {O}})$ and *Z* is Bayes in ${\mathcal {U}}({\mathcal {O}})$.

#### Proof

Since *Z* is not weakly dominated, it is also not strictly dominated. So, by Lemma D.3, there is some probability function $p^*$ on *Ω * with $p^*(Z)\geqslant p^*({\mathcal {U}}(o))$, for all *o*. Since ${\mathcal {O}}$ is Bayes-existing, there is some $o_{p^*}$ that is Bayes for $p^*$ in ${\mathcal {U}}({\mathcal {O}})$.

We will now show that ${\mathcal {U}}(o_{p^*})(\omega )\geqslant Z(\omega )$ for every *ω *. It will follow from this that ${\mathcal {U}}(o_{p^*})(\omega )=Z(\omega )$, for every *ω *, since we know that *Z* is not weakly dominated, and so ${\mathcal {U}}(o_{p^*})=Z$.

Hold fixed a single *ω *, which we call $\omega ^*$. Let $\pi _{\omega ^*}$ be the projection function for the ${\omega ^*}$ that we are considering, i.e., $\pi _{\omega ^*}(Y):=Y({\omega ^*})$. Then define $p_n$ by:

$$\begin{aligned} p_n=(1-{1}/{n})p^*+({1}/{n})\pi _{{\omega ^*}} \end{aligned}$$

Note that this depends on the $\omega ^*$ under consideration. Observe that $p_n$ is a probability function on *Ω *. And so, since ${\mathcal {O}}$ is Bayes-existing, for each *n*, there is some $o_{p_n}$ that is Bayes optimal for $p_n$, i.e., $p_n({\mathcal {U}}(o_{p_n}))\geqslant p_n({\mathcal {U}}(o))$, for all *o*. Since we have assumed $Z\in \textrm{cl}(\mathsf{ConvHull}({\mathcal {U}}({\mathcal {O}})))$, also $p_n( {\mathcal {U}}(o_{p_n}))\geqslant p_n(Z)$.^25^

We also know that $p^*(Z)\geqslant p^*({\mathcal {U}}(o_{p_n}))$. So, since $p_n$ is a mixture of $p^*$ and $\pi _{\omega ^*}$, to get that $p_n( {\mathcal {U}}(o_{p_n}))\geqslant p_n(Z)$ we must in fact have that $\pi _{{\omega ^*}}({\mathcal {U}}(o_{p_n}))\geqslant \pi _{\omega ^*}(Z)$. That is, we can conclude that ${\mathcal {U}}(o_{p_n})(\omega ^*)\geqslant Z(\omega ^*)$.

Observe that $p_n\longrightarrow p^*$. So, since ${\mathcal {U}}$ is Bayes-continuous on ${\mathcal {O}}$, ${\mathcal {U}}(o_{p_n})\longrightarrow {\mathcal {U}}(o_{p^*})$, so ${\mathcal {U}}(o_{p_n})({\omega })\longrightarrow {\mathcal {U}}(o_{p^*})({\omega })$ for each *ω *. Thus, since ${\mathcal {U}}(o_{p_n})({\omega ^*})\geqslant Z({\omega ^*})$ for all *n*, also ${\mathcal {U}}(o_{p^*})(\omega ^*)\geqslant Z(\omega ^*)$.

Now, this worked for any *ω *. That is, for any *ω * we can construct the relevant sequence and apply this argument. So, we have in fact shown that ${\mathcal {U}}(o_{p^*})(\omega )\geqslant Z(\omega )$ for all *ω *. And so, as noted above, it follows that ${\mathcal {U}}(o_{p^*})=Z$, for if ${\mathcal {U}}(o_{p^*})(\omega ) > Z(\omega )$ for any *ω *, ${\mathcal {U}}(o_{p^*})$ would weakly dominate *Z*, and, by assumption, this isn’t the case. *□ *

This proof in fact shows that the assumptions on ${\mathcal {O}}$ and ${\mathcal {U}}$ are very strong. It shows that for every probability there is a unique member of $\textrm{cl}(\mathsf{ConvHull}({\mathcal {U}}({\mathcal {O}})))$ that is not weakly dominated; thus also that for every regular probability, there is a unique Bayes option.

#### Theorem D.7

Suppose ${\mathcal {O}}$ is Bayes-existing and ${\mathcal {U}}$ is Bayes-continuous on ${\mathcal {O}}$. Then, if *o* is not Bayes then there is *o'* that strictly dominates it; moreover, it is strictly dominated by an option that is itself Bayes and not even weakly dominated.

#### Proof

If *o* is not Bayes in ${\mathcal {U}}({\mathcal {O}})$, then, by Lemma D.3, ${\mathcal {U}}(o)$ is strictly dominated in $\mathsf{ConvHull}({\mathcal {U}}({\mathcal {O}}))$.

By Lemma D.4 it is thus strictly dominated by some $Z\in \textrm{cl}(\mathsf{ConvHull}({\mathcal {U}}({\mathcal {O}})))$ which is itself not weakly dominated in $\mathsf{ConvHull}({\mathcal {U}}({\mathcal {O}}))$. By Lemma D.6, in fact $Z = {\mathcal {U}}(o_Z)$, for some $o_Z$ in ${\mathcal {O}}$, and moreover, $o_Z$ is Bayes, as required. *□ *

We now have two versions of Wald’s Complete Class Theorem: the first we proved along the way to proving the second. First: if ${\mathcal {U}}({\mathcal {O}})$ is convex then *o* is Bayes iff *o* is not strictly dominated. Second: if ${\mathcal {U}}$ is Bayes-continuous over ${\mathcal {O}}$ and ${\mathcal {O}}$ is Bayes-existing, then again *o* is Bayes iff *o* is not strictly dominated.

### D.2 Applying Wald’s Complete Class Theorem to probabilistic picking strategies for imprecise decision theories

- Fix $\mu ^*$ a measure over ${\mathcal {D}}$.
- The options, ${\mathcal {O}}$, are a specified collection of probabilistic picking strategies, ${\mathcal {N}}$.
- The utility of a probabilistic picking strategy ${\mathsf {n}}$ at *ω * is the expectation of the utility you’ll obtain at *ω * by picking in accordance with ${\mathsf {n}}$.
  $${\mathcal {U}}:{\mathcal {N}}\times \Omega \rightarrow [l,u]$$ defined by $${\mathcal {U}}({\mathsf {n}})(\omega ):=\mathsf{Exp}_{\mu ^*}[{\mathfrak {U}}({\mathsf {n}})(\omega )]$$.
  That is, $${\mathcal {U}}({\mathsf {n}})(\omega )=\mathsf{Exp}_{D\sim \mu ^*}[{\mathfrak {U}}({\mathsf {n}})(\omega ,D)]=\mathsf{Exp}_{D\sim \mu ^*}[\mathsf{Exp}_{a\sim {\mathsf {n}}_D}[{\mathfrak {U}}(a)(\omega )]]$$.

Recall Definition 4.3, which says when ${\mathcal {N}}$ is $\textrm{EU}$-complete.

#### Lemma D.8

Suppose ${\mathcal {N}}$ is $\textrm{EU}$-complete.

Then ${\mathsf {n}}$ is Bayes for *p* in ${\mathcal {O}}$, relative to ${\mathcal {U}}$, iff ${\mathsf {n}}$ $\mu ^*$-surely picks for $\textrm{EU}_p$.

Also ${\mathcal {N}}$ is Bayes-existing, relative to ${\mathcal {U}}$.

#### Proof

${\mathsf {n}}$ is Bayes for *p* in ${\mathcal {N}}$, relative to ${\mathcal {U}}$, iff for all ${\mathsf {n}}'\in {\mathcal {N}}$, $p({\mathcal {U}}({\mathsf {n}}))\geqslant p({\mathcal {U}}({\mathsf {n}}'))$. That is, $\mathsf{Exp}_p[\mathsf{Exp}_{\mu ^*}[{\mathfrak {U}}({\mathsf {n}})]]\geqslant \mathsf{Exp}_p[\mathsf{Exp}_{\mu ^*}[{\mathfrak {U}}({\mathsf {n}}')]]$. This is when ${\mathsf {n}}\in \textrm{EU}_{p\times \mu ^*}({\mathcal {N}})$.

By Corollary C.2, using the $\textrm{EU}$-completeness of ${\mathcal {N}}$, this is just when ${\mathsf {n}}$ $\mu ^*$-surely picks for $\textrm{EU}_p$.

By $\textrm{EU}$-completeness, for any *p* there is some such ${\mathsf {n}}\in {\mathcal {N}}$. So ${\mathcal {N}}$ is Bayes-existing, relative to ${\mathcal {U}}$. *□ *

Recall Definition 4.9, which says when a measure requires almost everywhere decisiveness.

#### Lemma D.9

Suppose ${\mathcal {N}}$ is $\textrm{EU}$-complete.

If ${\mu ^*}$ requires almost everywhere decisiveness, then ${\mathcal {U}}$ is Bayes continuous on ${\mathcal {N}}$.

#### Proof

Suppose $p^*$ is a probability function over *Ω *. And suppose $p_1,p_2\ldots$ is a sequence of probability functions over *Ω * that converges to $p^*$.

Take ${\mathsf {n}}^*\in {\mathcal {N}}$ Bayes for $p^*$ and ${\mathsf {n}}^1,{\mathsf {n}}^2,\ldots$ in ${\mathcal {N}}$ with ${\mathsf {n}}^k$ Bayes for $p_k$. This is possible by Lemma D.8, and also by that Lemma, ${\mathsf {n}}^*$ $\mu ^*$-surely picks for $\textrm{EU}_{p^*}$ and each ${\mathsf {n}}^k$ $\mu ^*$-surely picks for $\textrm{EU}_{p_k}$. That is, $\mu ^*(\lbrace {D \vert {\mathsf {n}}^*_{\;D}(\textrm{EU}_{p^*}(D)) = 1} \rbrace )=1$, and $\mu ^*(\lbrace {D \vert {\mathsf {n}}^k_{\;D}(\textrm{EU}_{p_k}(D)) = 1} \rbrace )=1$ for each *k*.

Let

$$\begin{aligned} E:=\lbrace {D\vert {\mathsf {n}}^*_{\;D}(\textrm{EU}_{p^*}(D)) = 1\text { and for all }k, {\mathsf {n}}^k_{\;D}(\textrm{EU}_{p_k}(D)) = 1} \rbrace . \end{aligned}$$

By countable additivity, $\mu ^*(E)=1$.

Let

$$\begin{aligned} S:=\{D\vert \textrm{EU}_{p^*}(D)\text { is a singleton}\}. \end{aligned}$$

As $\mu ^*$ requires almost everywhere decisiveness, $\mu ^*(S)=1$.

Take any $\omega ^*\in \Omega$ and $D^*\in E\cap S$.

$\textrm{EU}_{p^*}(D^*)$ is a singleton, so let $\textrm{EU}_{p^*}(D^*)=\{a^*\}$. As ${\mathsf {n}}^*_{\;D^*}(\{a^*\})=1$, observe that ${\mathfrak {U}}({\mathsf {n}}^*)(\omega ^*,D^*)={\mathfrak {U}}(a^*)(\omega ^*)$.

Expected utility is continuous as a function of probabilities; i.e., for all $a\in {\mathcal {A}}$, $\mathsf{Exp}_{p_k}[{\mathfrak {U}}(a)]\longrightarrow \mathsf {Exp}_{p^*}[{\mathfrak {U}}(a)]$. Thus, as $D^*$ is finite, there is some $N\in {\mathbb {N}}\setminus \{0\}$ such that for all *k>N*, $\textrm{EU}_{p_k}(D^*)=\{a^*\}$.^26^

And so, for each *k>N*, ${\mathsf {n}}^k_{\;D^*}(\{a^*\})=1$ and so ${\mathfrak {U}}({\mathsf {n}}^k)(\omega ^*,D^*)={\mathfrak {U}}(a^*)(\omega ^*)={\mathfrak {U}}({\mathsf {n}}^*)(\omega ^*,D^*)$.

This gives us that ${\mathfrak {U}}({\mathsf {n}}^k)(\omega ^*,D^*)\longrightarrow {\mathfrak {U}}({\mathsf {n}}^*)(\omega ^*,D^*)$.

So we have that for any $D\in E\cap S$, ${\mathfrak {U}}({\mathsf {n}}^k)(\omega ^*,D)\longrightarrow {\mathfrak {U}}({\mathsf {n}}^*)(\omega ^*,D)$. As we know that $\mu ^*(E\cap S)=1$, we can use a Dominated Convergence Theorem (as utilities are bounded) to obtain $\mathsf{Exp}_{\mu ^*}[{\mathfrak {U}}({\mathsf {n}}^k)(\omega ^*)]\longrightarrow \mathsf{Exp}_{\mu ^*}[{\mathfrak {U}}({\mathsf {n}}^*)(\omega ^*)]$. That is, ${\mathcal {U}}({\mathsf {n}}^k)(\omega ^*)\longrightarrow {\mathcal {U}}({\mathsf {n}}^*)(\omega ^*)$.

As this holds for any $\omega \in \Omega$, we have that ${\mathcal {U}}$ is Bayes continuous on ${\mathcal {N}}$. *□ *

#### Corollary D.10

Suppose that ${\mathcal {N}}$ is EU-complete, and suppose $\mu ^*$ requires almost everywhere decisiveness.

If there is no probability *p* over *Ω * for which ${\mathsf {n}}$ maximises $\mathsf{Exp}_{p}[\mathsf{Exp}_{\mu ^*}[{\mathfrak {U}}({\mathsf {n}})]]$; then there is some ${\mathsf {n}}'$ such that $\mathsf{Exp}_{\mu ^*}[{\mathfrak {U}}({\mathsf {n}}')(\omega )]>\mathsf{Exp}_{\mu ^*}[{\mathfrak {U}}({\mathsf {n}})(\omega )]$ for all $\omega \in \Omega$.

#### Proof

This is immediate from Theorem D.7 and Lemmas D.8 and D.9. *□ *

#### Corollary D.11

Suppose that ${\mathcal {N}}$ is EU-complete, $\mu ^*$ requires almost everywhere decisiveness.

If ${\mathsf {n}}$ does not $\mu ^*$-surely pick for $\textrm{EU}_p$ for any probability *p*, then there is some ${\mathsf {n}}'$ such that for all probabilities *p*, $\mathsf{Exp}_{p\times \mu ^*}[{\mathfrak {U}}({\mathsf {n}}')]>\mathsf{Exp}_{p\times \mu ^*}[{\mathfrak {U}}({\mathsf {n}})]$.

#### Proof

By Theorem C.1, if ${\mathsf {n}}$ does not $\mu ^*$-surely pick for any probability *p*, ${\mathsf {n}}$ does not maximise $\mathsf{Exp}_{p\times \mu ^*}[{\mathfrak {U}}({\mathsf {n}})]$ for any *p*. Observe, also that $\mathsf{Exp}_p[\mathsf{Exp}_{\mu ^*}[{\mathfrak {U}}({\mathsf {n}})]]=\mathsf{Exp}_{p\times \mu ^*}[{\mathfrak {U}}({\mathsf {n}})]$. So by Corollary D.10, there is ${\mathsf {n}}'$ with $\mathsf{Exp}_{\mu ^*}[{\mathfrak {U}}({\mathsf {n}}')(\omega )]>\mathsf{Exp}_{\mu ^*}[{\mathfrak {U}}({\mathsf {n}})(\omega )]$ for all *ω *; and thus, $\mathsf{Exp}_{p}[\mathsf{Exp}_{\mu ^*}[{\mathfrak {U}}({\mathsf {n}}')]]\,>\mathsf{Exp}_p[\mathsf{Exp}_{\mu ^*}[{\mathfrak {U}}({\mathsf {n}})]]$ for all probabilities *p*; which gives us the claim. *□ *

Proposition 4.10 follows from this. Proposition 4.12 follows from this together with Corollary 4.11, since Maximality is more permissive than E-Admissibility.

We now prove Proposition 4.15. Let ${\mathsf {c}}$ be any choice function.

#### Proposition D.12

(Proposition 4.15) For any choice function ${\mathsf {c}}$, if $\mu ^*$ is atomless and for all $p\in {\mathcal {P}}$, $\mu ^*\{D\vert {\mathsf {c}}(D)\subseteq \textrm{EU}_p(D)\}<1$, then there is ${\mathsf {s}}$ which picks for ${\mathsf {c}}$ but for no $p\in {\mathcal {P}}$ does it $\mu ^*$-surely pick for $\textrm{EU}_p$.

#### Proof

Choose $$q\in {\mathcal {P}}$$.

Let $$t_q(D):=\sup _{a \in D} \mathsf{Exp}_q[{\mathfrak {U}}(a)]$$.

Let $$G(q,D,\epsilon ):=\{b\in D\vert t_q(D)>\mathsf{Exp}_q[{\mathfrak {U}}(b)]+\epsilon \}$$.

#### Sublemma D.12.1

There is some $\epsilon _q>0$ s.t. $\mu ^*\{D\vert G(q,D,\epsilon _q)\cap {\mathsf {c}}(D)\ne \varnothing \}>0$.

#### Proof

We have assumed that $\mu ^*\{D\vert {\mathsf {c}}(D)\not \subseteq \textrm{EU}_q(D)\}>0$, for all $q\in {\mathcal {P}}$.

Observe

$$\begin{aligned} \{D\vert {\mathsf {c}}(D)\not \subseteq \textrm{EU}_q(D)\}&=\{D\vert \exists b\in {\mathsf {c}}(D)\text { and }t_q(D)>\mathsf{Exp}_q[{\mathfrak {U}}(b)] \} \\&=\bigcup _{k\in {\mathbb {N}}}\{D\vert \exists b\in {\mathsf {c}}(D)\text { and }t_q(D)>\mathsf{Exp}_q[{\mathfrak {U}}(b)]+{1}/{k} \} \\&=\bigcup _{k\in {\mathbb {N}}}\{D\vert G(q,D,{1}/{k})\cap c(D)\ne \varnothing \} \end{aligned}$$

so it follows from countable additivity that there is some *k* such that $\mu ^*\{D\vert G(q,D,{1}/{k})\cap {\mathsf{c}}(D)\ne \varnothing \}>0$. This gives us our $\epsilon _q$ as ${1}/{k}$ for this *k*. *□ *

We have assumed that utility is bounded. Put *M>0* such that $\Vert {\mathfrak {U}}(a)\Vert _\infty =\sup \{|{\mathfrak {U}}(a)(\omega )|\vert \omega \in \Omega \}\leqslant M$ for all $a\in {\mathcal {A}}$, that is $M\geqslant \max \{|l|,|h|\}$ if ${\mathfrak {U}}:{\mathcal {A}}\rightarrow [l,h]^\Omega$. Let

$$\begin{aligned} U_q:=\{p\in {\mathcal {P}}\vert \Vert q-p\Vert _1<\frac{\epsilon _q}{4M}\}. \end{aligned}$$

These are open, and cover ${\mathcal {P}}$ as $p\in U_p$. So by compactness, there is a finite sub-cover, $U_{q_1},\ldots , U_{q_n}$.

Let

$$\begin{aligned} C_i:=\{D\vert G(q_i,D,\epsilon _{q_i})\cap {\mathsf {c}}(D)\ne \varnothing \}. \end{aligned}$$

and note that we have $\mu ^*(C_i)>0$ by choice of $\epsilon _{q_i}$.

#### Sublemma D.12.2

We can choose $E_i\subseteq C_i$ pairwise disjoint and with $\mu ^*(E_i)>0$

#### Proof

This uses the atomlessness of $\mu ^*$.

Put $$\alpha =\min \{\mu ^*(C_1),\ldots ,\mu ^*(C_k)\}$$.

Choose $E_1\subseteq C_1$ with $0<\mu ^*(E_1)\leqslant \frac{\alpha }{k}$ (possible since $\mu ^*$ is atomless).

Then $\mu ^*(C_2{\setminus } E_1)\geqslant \alpha -\frac{\alpha }{k}\geqslant \frac{\alpha }{k}$ so we can choose $E_2\subseteq C_2\setminus E_1$ with $0<\mu ^*(E_2)\leqslant \frac{\alpha }{k}$.

Then $\mu ^*(C_3{\setminus }(E_1\cup E_2))\geqslant \alpha -2\frac{\alpha }{k}\geqslant \frac{\alpha }{k}$ so we can choose $E_3\subseteq C_3\setminus (E_1\cup E_2)$ with $0<\mu ^*(E_3)\leqslant \frac{\alpha }{k}$.

And so on, with the final stage ensuring $\mu ^*(C_k{\setminus }(E_1\cup \ldots \cup E_{k-1}))\geqslant \alpha -(k-1)\frac{\alpha }{k}=\frac{\alpha }{k}$ so we can choose $E_k\subseteq C_k\setminus (E_1\cup \ldots \cup E_{k-1})$ with $0<\mu ^*(E_k)\leqslant \frac{\alpha }{k}$. *□ *

Then put ${\mathsf {s}}(D)\in G(q_i,D,\epsilon _{q_i})\cap {\mathsf {c}}(D)$ for $D\in E_i\subseteq C_i$, and ${\mathsf {s}}(D)\in {\mathsf {c}}(D)$ for $D\notin \bigcup _{i=1,\ldots ,k} E_i$.

Observe that ${\mathsf {s}}(D)$ picks for ${\mathsf {c}}$ by definition.

Take any $p\in {\mathcal {P}}$. As $U_{q_1},\ldots , U_{q_n}$ cover ${\mathcal {P}}$, there is some *i* such that $p\in U_{q_i}$.

We will show that for any $D\in E_i$, ${\mathsf {s}}(D)\notin \textrm{EU}_p(D)$. This will use the following sublemma:

#### Sublemma D.12.3

Suppose $p\in U_q$. If $b\in G(q,D,\epsilon _q)$ then $b\notin \textrm{EU}_p(D)$.

#### Proof

As $b\in G(q,D,\epsilon _q)$, $t_q(D)>\mathsf{Exp}_q[{\mathfrak {U}}(b)]+\epsilon _q$, where $t_q(D)=\sup _{a \in D}\mathsf{Exp}_q[{\mathfrak {U}}(a)]$. Take $a^*\in \textrm{EU}_q(D)$ so that $\mathsf{Exp}_q[{\mathfrak {U}}(a^*)]=t_q(D)$ which implies $\mathsf{Exp}_q[{\mathfrak {U}}(a^*)]-\mathsf{Exp}_q[{\mathfrak {U}}(b)]\geqslant \epsilon _q$.

As $p\in U_q$, we have $\Vert q-p\Vert _1<\frac{\epsilon _q}{4M}$.

So

$$\begin{aligned} \mathsf{Exp}_p[{\mathfrak {U}}(a^*)]-\mathsf{Exp}_p[{\mathfrak {U}}(b)]&=\left( \mathsf{Exp}_q[{\mathfrak {U}}(a^*)]-\mathsf{Exp}_q[{\mathfrak {U}}(b)]\right) +(p-q)\cdot ({\mathfrak {U}}(a^*)-{\mathfrak {U}}(b))\\&\geqslant \epsilon _q -|(p-q)\cdot ({\mathfrak {U}}(a^*)-{\mathfrak {U}}(b))|\\&\geqslant \epsilon _q -\Vert p-q\Vert _1\Vert {\mathfrak {U}}(a^*)-{\mathfrak {U}}(b)\Vert _\infty \ \text {(H}{\ddot{{\textrm{o}}}}\text {lder's inequality)}\\&\geqslant \epsilon _q -\frac{\epsilon _q}{4M}\times 2M=\epsilon _q -{\epsilon _q}/{2}={\epsilon _q}/{2}>0 \end{aligned}$$

This shows that $b\notin \textrm{EU}_p(D)$. *□ *

Since we have chosen ${\mathsf {s}}(D)\in G(q_i,D,\epsilon _{q_i})$ for $D\in E_i$, we have $\{D\vert {\mathsf {s}}(D)\notin \textrm{EU}_p(D)\}\supseteq E_i$, and thus, $\mu ^*\{D\vert {\mathsf {s}}(D)\notin \textrm{EU}_p(D)\}>0$. *□ *

#### Proposition D.13

(Proposition 4.17) Suppose ${\mathcal {N}}$ is $\textrm{EU}$-complete. Suppose that ${\mathbb {B}}$ has the form $\{p\times \mu ^*\vert p\in {\mathbb {P}}\}$ and $\mu ^*$ requires almost everywhere decisiveness. Suppose that, for every probability *p*, $\mu ^*\{D\vert \textrm{Max}_{\mathbb {P}}(D)\subseteq \textrm{EU}_p(D)\}< 1$. Then, if ${\mathsf {n}}$ is a regular picking strategy for $\textrm{Max}_{{\mathbb {B}}(\cdot |-)}$ then ${\mathsf {n}}\notin \textrm{Max}_{\mathbb {B}}({\mathcal {N}})$.

#### Proof

Suppose ${\mathsf {n}}$ is a regular picking strategy for $\textrm{Max}_{{\mathbb {B}}(\cdot |-)}$ and the conditions of the theorem hold.

Take any $p\in {\mathbb {P}}$. If $\textrm{Max}_{{\mathbb {B}}(\cdot |-)}(D)\not \subseteq \textrm{EU}_p(D)$, then, since ${\mathsf {n}}_D$ is a regular probability function, ${\mathsf {n}}_D(\textrm{EU}_p(D))< 1$. Since $b_{\mathcal {D}}\{D\vert \textrm{Max}_{{\mathbb {B}}(\cdot |-)}(D)\not \subseteq \textrm{EU}_p(D)\}>0$, we have $\mu ^*\{D\vert {\mathsf {n}}(\textrm{EU}_p(D))<1\}>0$. So ${\mathsf {n}}$ does not $\mu ^*$-surely pick for $\textrm{EU}_p$. Since this holds for any $p \in {\mathbb {P}}$, Corollary D.11 tells us that ${\mathsf {n}}\notin \textrm{Max}_{\mathbb {B}}({\mathcal {N}})$. *□ *

#### Proposition D.14

(Proposition 4.18) Suppose ${\mathcal {N}}$ is $\textrm{EU}$-complete. Suppose that ${\mathbb {B}}$ has the form $\{p\times \mu ^*\vert p\in {\mathbb {P}}\}$ and $\mu ^*$ requires almost everywhere decisiveness. Then if ${\mathsf {n}}\in \Gamma _{\mathbb {B}}({\mathcal {N}})$, there is some probability $p\in {\mathcal {P}}$ where ${\mathsf {n}}$ $\mu ^*$-surely picks for $\textrm{EU}_p$.

Suppose further that for every probability $p\in {\mathcal {P}}$, $\mu ^*\{D\vert \Gamma _{\mathbb {P}}(D)\subseteq \textrm{EU}_p(D)\}<1$. Then if ${\mathsf {n}}$ is regular for $\Gamma _{\mathbb {P}}$ then ${\mathsf {n}}\notin \Gamma _{\mathbb {B}}({\mathcal {N}})$.

#### Proof

Suppose that ${\mathsf {n}}\in \Gamma _{\mathbb {B}}({\mathcal {N}})\subseteq \textrm{Max}_{\mathbb {B}}({\mathcal {N}})$. By Proposition 4.10, there is some probability $p\in {\mathcal {P}}$ where ${\mathsf {n}}$ $\mu ^*$-surely picks for $\textrm{EU}_p$.

Suppose ${\mathsf {n}}$ is a regular picking strategy for $\Gamma _{\mathbb {P}}$ and the conditions above hold.

Take any $p\in {\mathcal {P}}$. If $\Gamma _{{\mathbb {P}}}(D)\not \subseteq \textrm{EU}_p(D)$, then, since ${\mathsf {n}}_D$ is a regular probability function, ${\mathsf {n}}_D(\textrm{EU}_p(D))< 1$. Since $\mu ^*\{D\vert \Gamma _{\mathbb {P}}(D)\not \subseteq \textrm{EU}_p(D)\}>0$, we have $\mu ^*\{D\vert {\mathsf {n}}(\textrm{EU}_p(D))<1\}>0$. So ${\mathsf {n}}$ does not $\mu ^*$-surely pick for $\textrm{EU}_p$. Since this holds for any $p \in {\mathcal {P}}$, we must have ${\mathsf {n}}\not \in \Gamma _{\mathbb {B}}({\mathcal {N}})$. *□ *

## Footnotes

1 This is an Archimedeanicity principle.

2 In the case where we have $a_1\succ a_2$ and $\alpha a_1 +(1-\alpha )b\prec \alpha a_2 + (1-\alpha )b$, this is immediate. If the reversal is merely weak, so that $a_1\succ a_2$ and $\alpha a_1 +(1-\alpha )b\preceq \alpha a_2 + (1-\alpha )b$, we appeal to an Ordering principle, to give $\alpha a_1 +(1-\alpha )b\sim \alpha a_2 + (1-\alpha )b$, and an Archimedeanicity principle to produce $a^+_2$ such that $a_1\succ a^+_2$ and $\alpha a_1 +(1-\alpha )b\prec \alpha a^+_2 + (1-\alpha )b$.

3 Cf. (Armendt, 1993; Mahtani, 2015).

4 This is not Buchak’s favoured formulation; instead it’s closer to the usual formulation of rank dependent expected utility theory; see, for example (Wakker, Chapter 6 2010) See also sec.6.9 for the continuous case, although note that Buchak’s theory does not make use of a distinction between gains and losses (see Buchak, 2013, p59).

5 See the Mathematica notebook here for the tools to carry out these calculations (see supplementary material).

6 We will also assume that ${\mathfrak {U}}({\mathcal {A}})$ is a Borel set in ${\mathbb {R}}^\Omega \cong {\mathbb {R}}^n$.

7 In fact, we restrict attention to the picking strategies that are measurable in the sense defined in Appendix A.

8 The version we present here is of the sort described by Savage (1954), in which it is assumed that the acts are independent of the states of the world. This assumption is dropped in the evidential decision theory of Jeffrey (1965) and the causal decision theory of Stalnaker (1972); Gibbard and Harper (1978); Joyce (1999).

9 The details of this are developed in Appendix B.

10 It is standard in the imprecise probability literature to reserve the term “credal set” for convex sets of probability measures. We do not assume convexity here.

11 In fact, we have added a small constant to the usual versions of 1F and 2E, reflecting the fact that people strictly prefer the usual version of 1E over the usual version of 1F, and so are willing to pay a penalty for making that choice; we’ve taken that penalty to be $\text {\pounds1}$, but our point remains however small you make it.

12 This is because, to have $\mathsf{Exp}_p[{\mathfrak {U}}(\text {1E)}]\geqslant \mathsf{Exp}_p[{\mathfrak {U}}(\text {1F)}]$, it must be that $x\leqslant {7}/{30}$; and, to have $\mathsf{Exp}_p[{\mathfrak {U}}(\text {2F)}]\geqslant \mathsf{Exp}_p[{\mathfrak {U}}(\text {2E)}]$, it must be that $x\geqslant {13}/{30}$; and these are jointly incompatible.

13 For any $A\subseteq \Omega$, $\omega \in A:=\left\{ \left<\omega ,D\right>\in \Omega \times {\mathcal {D}}\vert \omega \in A\right\}$. Likewise, for any $E\subseteq {\mathcal {D}}$, $D\in E:=\left\{ \left<\omega ,D\right>\in \Omega \times {\mathcal {D}}\vert D\in E\right\}$.

14 A short survey of independence notions for imprecise probability: complete independence for sets of probabilities (Seidenfeld (2007); Cozman (2012)); independence in selection for lower previsions (de Campos and Moral (1995)); strong independence for lower previsions and sets of desirable gambles (de Cooman and Miranda (2012b)); epistemic independence (value and subset) for sets of desirable gambles (Moral (2005)); epistemic h-independence for lower previsions and credal sets (De Bock (2015)); S-independence for choice functions (De Bock and de Cooman (2021)).

15 See Footnote 12.

16 The example using the Ellsberg case does not have this property, which requires many decision problems to be possible. If, for example, we had selected $\mu ^*(D^{\textrm{Ellsberg}}_1)=0.1$, then one can check that no strategies are ruled out by Maximality.

17 These examples are avoided when ${\mathbb {P}}$ is convex as then there will be a probability $p^*\in {\mathbb {P}}$ which is indifferent between the two actions, and thus, ${\mathsf {n}}\in \textrm{EU}_{p^*}({\mathcal {N}})$.

18 The assumptions on $\mu ^*$ are not required if one instead assumes that ${\mathcal {N}}$ is convex, which is motivated when one considers randomisations as available options. If *D* was allowed to be infinite, one should also ensure that ${\mathcal {N}}$ is closed, for which one needs to allow merely finitely additive randomisations (Schervish et al., 2020).

19 Equivalently, that for every probability *p* there is a non-negligible set of decision problems for which there is some *a∈ D* which is not an $\textrm{EU}_p$ act but which is Maximal: $\mu ^*\{D\vert \exists a\in D\left[ (\exists b{\in } D \, \mathsf{Exp}_{p}[\mathfrak{U}(a)]<\mathsf{Exp}_{p}[\mathfrak{U}(b)])\text { and }\forall b{\in } D\, \exists p'{\in }{\mathbb {P}}\,\mathsf{Exp}_{p'}[\mathfrak{U}(b)]\leqslant \mathsf{Exp}_{p'}[\mathfrak{U}(a)]\right] \}>0$  

20 Or perhaps this second disjunct should say that there is some $a\in {\mathbb {A}}$ and $a'\in {\mathbb {A}}'$ with $\mathsf{Exp}_p[{\mathfrak {U}}(a')]>\mathsf{Exp}_p[{\mathfrak {U}}(a)]$, but since our application of interest satisfies the slightly stronger property, we merely impose that.

## References

[CR1] Aliprantis, C. D., & Border, K. C. (2006). *Infinite dimensional analysis: a hitchhiker’s guide*. Springer Science & Bsiness Media.

[CR2] Allais, M. (1953). Le comportement de l’homme rationnel devant le risque: critique des postulats et axiomes de l’école Américaine. *Econometrica,**21*(4), 503–546.

[CR3] Armendt, B. (1993). Dutch Books, Additivity, and Utility Theory. *Philosophical Topics,**21*(1).

[CR4] Boyd, S., & Vandenberghe, L. (2004). *Convex Optimization*. Cambridge, UK: Cambridge University Press.

[CR5] Bradley, S. (2016). Imprecise Probabilities. In E. N. Zalta (Ed.), *Stanford Encyclopedia of Philosophy. *Metaphysics Research Lab: Stanford University.

[CR6] Brown, P. M. (1976). Conditionalization and expected utility. *Philosophy of Science,**43*(3), 415–419.

[CR7] Buchak, L. (2013). *Risk and Rationality*. Oxford, UK: Oxford University Press.

[CR8] Campbell-Moore, C., & Salow, B. (2020). Avoiding Risk and Avoiding Evidence. *Australasian Journal of Philosophy,**98*(3), 495–515.

[CR9] Campbell-Moore, C., & Salow, B. (2022). Accurate Updating for the Risk-Sensitive. *British Journal for the Philosophy of Science,**73*(3), 751–776.

[CR10] Cozman, F. G. (2012). Sets of probability distributions, independence, and convexity. *Synthese,**186*, 177–200.

[CR11] De Bock, J. (2015). *Credal Networks under Epistemic Irrelevance: Theory and algorithms*. Ph.D. thesis, Ghent University.

[CR12] Bock, J. (2019). Independent natural extension for infinite spaces. *International Journal of Approximate Reasoning,**104*, 84–107.

[CR13] Bock, J., & Cooman, G. (2014). An efficient algorithm for estimating state sequences in imprecise hidden markov models. *Journal of Artificial Intelligence Research,**50*, 189–233.

[CR14] De Bock, J., & de Cooman, G. (2021). On a notion of independence proposed by Teddy Seidenfeld. arxiv:2102.10342

[CR15] Campos, L. M., & Moral, S. (1995). Independence concepts for convex sets of probabilities. In P. Besnard & S. Hanks (Eds.), *Eleventh Conference on Uncertainty in Artificial Intelligence* (pp. 108–115). CA: San Francisco.

[CR16] Cooman, G., & Miranda, E. (2012). Irrelevant and independent natural extensionfor sets of desirable gambles. *Journal of Artificial Intelligence Research,**45*, 601–640.

[CR17] Cooman, G., & Miranda, E. (2012). Irrelevant and independent natural extension for sets of desirable gambles. *Journal of Artificial Intelligence Research,**45*, 601–640.

[CR18] Elga, A. (2010). Subjective Probabilities Should Be Sharp. *Philosophers’ Imprint,**10*(5), 1–11.

[CR19] Ellsberg, D. (1961). Risk, Ambiguity, and the Savage Axioms. *Quarterly Journal of Economics,**75*(4), 643–69.

[CR20] Gibbard, A., & Harper, W. (1978). Counterfactuals and Two Kinds of Expected Utility. In E. F. M. Clifford Alan Hooker, James L. Leach (Ed.) *Foundations and Applications of Decision Theory*, vol. 13a of *University of Western Ontario Series in Philosophy of Science*, (pp. 125–162). D. Reidel.

[CR21] Hammond, P. (1988). Consequentialist Foundations for Expected Utility. *Theory and Decision,**25*, 25–78.

[CR22] Jeffrey, R. (1965). *The Logic of Decision*. New York: McGraw-Hill.

[CR23] Joyce, J. M. (1999). *The Foundations of Causal Decision Theory*. Cambridge Studies in Probability, Induction, and Decision Theory. Cambridge: Cambridge University Press.

[CR24] Kahneman, D., & Tversky, A. (1979). Prospect Theory: An Analysis of Decision under Risk. *Econometrica,**47*(2), 263.

[CR25] Kallenberg, O. (2021). *Foundations of modern probability*, vol. 99 of *Probability Theory and Stochastic Modelling*. Switzerland: Springer, 3 ed.

[CR26] Levi, I. (1999). Imprecise and indeterminate probabilities. *Proceedings of ISIPTA’99*, (pp. 258–265).

[CR27] Levinstein, B. A. (2017). A Pragmatist’s Guide to Epistemic Utility. *Philosophy of Science,**84*(4), 613–638.

[CR28] Lewis, D. (1971). Immodest Inductive Methods. *Philosophy of Science,**38*(1), 54–63.

[CR29] Machina, M. J. (1982). Expected Utility. *Analysis without the Independence Axiom. Econometrica,**50*(2), 277–323.

[CR30] Machina, M. J. (1989). Dynamic consistency and non-expected utility models of choice under uncertainty. *Journal of Economic Literature,**27*, 1622–1668.

[CR31] Mahtani, A. (2015). Dutch Books, Coherence, and Logical Consistency. *Noûs,**49*(3), 522–537.

[CR32] Marschak, J. (1950). Rational Behavior, Uncertain Prospects, and Measurable Utility. *Econometrica,**18*(2), 111–141.

[CR33] Moral, S. (2005). Epistemic irrelevance on sets of desirable gambles. *Annals of Mathematics and Artificial Intelligence,**45*, 197–214.

[CR34] Moss, S. (2015). Time-Slice Epistemology and Action under Indeterminacy. In T. S. Gendler & J. Hawthorne (Eds.), *Oxford Studies in Epistemology* (pp. 172–94). Oxford, UK: Oxford University Press.

[CR35] Pettigrew, R. (2020). *Dutch book arguments*. Cambridge University Press.

[CR36] Quiggin, J. (1982). A Theory of Anticipated Utility. *Journal of Economic Behavior and Organization,**3*, 323–43.

[CR37] Quiggin, J. (1993). *Generalized Expected Utility Theory: The Rank-Dependent Model*. Kluwer Academic Publishers.

[CR38] Savage, L. J. (1954). *The Foundations of Statistics*. John Wiley & Sons.

[CR39] Schervish, M. J. (1989). A general method for comparing probability assessors. *The Annals of Statistics,**17*, 1856–1879.

[CR40] Schervish, M. J., Seidenfeld, T., & Kadane, J. B. (2009). Proper Scoring Rules, Dominated Forecasts, and Coherence. *Decision Analysis,**6*(4), 202–221.

[CR41] Schervish, M. J., Seidenfeld, T., Stern, R. B., & Kadane, J. B. (2020). What finite-additivity can add to decision theory. *Statistical Methods & Applications,**29*(2), 237–263.

[CR42] Seidenfeld, T. (2004). A contrast between two decision rules for use with (convex) sets of probabilities: -maximin versus e-admissibility. *Synthese,**140*(1/2), 69–88.

[CR43] Seidenfeld, T. (2007). *Conditional independence, imprecise probabilities, null-events and graph-theoretic models*. Seimour Geisser Distinguished Lecture: School of Statistics, University of Minnesota, Minneapolis, MN.

[CR44] Stalnaker, R. C. (1972 [1981]). Letter to David Lewis. In W. L. Harper, R. C. Stalnaker, & G. Pearce (Eds.) *Ifs: Conditionals, Belief, Decision, Chance and Time*, University of Western Ontario Series in Philosophy of Science, (pp. 151–2). Dordrecht: Reidel.

[CR45] Wakker, P. P. (2010). *Prospect theory: For risk and ambiguity*. Cambridge University Press.

[CR46] Wald, A. (1947). An essentially complete class of admissible decision functions. *Annals of Mathematical Statistics,**18*(4), 549–555.

[CR47] Walley, P. (1991). *Statistical Reasoning with Imprecise Probabilities*, vol. 42 of *Monographs on Statistics and Applied Probability*. London: Chapman and Hall

